# Striking essential oil: tapping into a largely unexplored source for drug discovery

**DOI:** 10.1038/s41598-020-59332-5

**Published:** 2020-02-18

**Authors:** Adam F. Feyaerts, Walter Luyten, Patrick Van Dijck

**Affiliations:** 1 VIB Center for Microbiology, KU Leuven, 3001 Leuven, Belgium; 2 Laboratory of Molecular Cell Biology, KU Leuven, 3001 Leuven, Belgium; 3 Department of Biology, KU Leuven, 3000 Leuven, Belgium

**Keywords:** Drug discovery, Microbiology, Molecular biology, Plant sciences, Infectious diseases, Chemical genetics, Chemical libraries

## Abstract

Essential oils (EOs) have been used therapeutically for centuries. In recent decades, randomized controlled (clinical) trials have supported efficacy in specific therapeutic indications for a few of them. Some EOs, their components or derivatives thereof have been approved as drugs. Nevertheless, they are still considered products that are mainly used in complementary and alternative medicine. EO components occupy a special niche in chemical space, that offers unique opportunities based on their unusual physicochemical properties, because they are typically volatile and hydrophobic. Here we evaluate selected physicochemical parameters, used in conventional drug discovery, of EO components present in a range of commercially available EOs. We show that, contrary to generally held belief, most EO components meet current-day requirements of medicinal chemistry for good drug candidates. Moreover, they also offer attractive opportunities for lead optimization or even fragment-based drug discovery. Because their therapeutic potential is still under-scrutinized, we propose that this be explored more vigorously with present-day methods.

## Introduction

Over the past few decades, drug discovery has shifted mainly to high-throughput screening of large chemical libraries while research on natural products has diminished^1,2^. Reasons for this are among others: (i) the legitimate concerns about the United Nations Convention on Biological Diversity and the Nagoya protocol which regulates the sovereign rights of genetic resources, resulting in many years of legal uncertainty regarding derived rights^3,4^. (ii) The difficulty of patent protection for natural products, especially since the publication of the “Guidance for Determining Subject Matter Eligibility of Claims Reciting or Involving Laws of Nature, Natural Phenomena, & Natural Products”^5,6^. (iii) The belief that natural products are somehow incompatible with high-throughput screening, and (iv) that they are difficult to isolate or synthesize^7^. At the same time, some compound collections are designed to mimic natural products, because these also offer clear advantages; for example, they exhibit a wide range of pharmacophores, show a high degree of stereochemistry and are metabolite-like^2,4,8–13^. However, recent technical developments and increased legal certainty have led to a renewed interest in natural product drug discovery^1,2,4,7,12,14–16^. This is further strengthened by the 2015 Nobel Prize in Physiology or Medicine for natural product research on artemisinin; a sesquiterpene lactone with anti-malarial properties from the plant *Artemisia annua*, and on avermectins; macrocyclic lactones with potent anthelmintic and insecticidal properties derived from the soil bacterium *Streptomyces avermitilis*^7,17^.

Companies have made great efforts to increase the chemical diversity of their compound collections used for drug discovery, but at the same time to reduce especially late-stage attrition; as a result the composition of these collections is increasingly shaped, or even dictated, by requirements for lead- and drug-likeness^4,18–20^. To evaluate the lead- and drug-likeness of candidate molecules *in silico*, drug discovery filters (DDFs) are used^21–23^. DDFs are essentially sets of simple rules, determining whether a molecule meets criteria for one or more specific (derived) physicochemical parameters, further referred to as drug discovery parameters (DDPs). To qualify, a molecule must meet all or most criteria of the DDPs included in a specific DDF^21,23^. These DDFs were initially derived from collections of marketed medicines, but later more specific DDFs have been developed for specific applications e.g. fragment-based drug discovery^24,25^. The use of DDFs has led to lower attrition rates, i.e. fewer false positives, in drug development, although recent results suggest that further control of physicochemical properties is unlikely to affect attrition rates significantly^26,27^. DDFs, however, render a large area of chemical space “off-limits” for medicinal chemists, so that otherwise interesting molecules are not even considered^4,28^. For instance, many natural products would be excluded by Lipinski’s well-known Rule of Five (Ro5). This rule of thumb, consisting of a subset of the studied DDPs with specific criteria (Table 1), is used to evaluate whether a molecule is likely to be orally bioavailable, but without predicting whether the molecule is pharmacologically active^21,29^. Lipinski himself excluded natural products from his Ro5 when establishing the criteria^30^. Nonetheless, a substantial number of natural products meet the Ro5 DDP criteria^31^. Moreover, some natural products that do not meet the Ro5 DDP criteria, nonetheless exhibit good oral bioavailability, probably through co-evolution^32,33^.

**Table 1:** Drug Discovery Parameter criteria used in Drug Discovery Filters available in JChem^21–23,56–58^. #: number of; —: not applicable.

Drug Discovery Parameter	Drug Discovery Filter					
	Ro5 ≥ 3 criteria	Lead Likeness	Ghose	Muegge	Veber	Bio-availability ≥ 6 criteria
molecular mass (Da)	≤500	≤450	≥160 ≤480	≥200 ≤600	—	≤500
log P^&^	≤5.0	>−3.5 <4.5^£^	≥−0.4 ≤5.6	≥−2.0 ≤5.0	—	≤5.0
H-bond donors (#)	≤5	≤5	—	≤5	sum ≤12^§^	≤10
H-bond acceptors (#)	≤10	≤8	—	≤10		≤10
log D (pH 7.4)^&^	—	≥−4.0 ≤4.0^£^	—	—	—	—
molecular rings (#)	—	≤4	—	≤7	—	—
rotatable bonds (#)	—	≤10	—	≤15	≤10	≤10
atoms (#)	—	—	≥20 ≤70	—	—	—
molar refractivity^&^ (m^3^ mol^−1^)	—	—	≥40 ≤130	—	—	—
C-atoms (#)	—	—	—	≥5	—	—
Muegge’s atoms (#)^&^	—	—	—	≥2	—	—
(topological) polar surface area^&^ (10^−10^ m)	—	—	—	≤150	≤140^§^	≤200
fused aromatic rings (#)	—	—	—	—	—	≤5

^&^For more details on the Drug Discovery Parameters, we refer to the materials and methods section. ^£,§^The Lead Likeness and Veber Drug Discovery Filters can alternatively use one or the other criterion^22,57^: For the Lead Likeness and Veber Drug Discovery Filters, log D (pH 7.4) and polar surface, respectively, are considered the standard Drug Discovery Parameters.

Natural product-based drug discovery is considered intrinsically complex and requires a highly integrated interdisciplinary approach^1,2,4,7,34,35^. Essential oils (EOs) are plant-based natural products that have been used therapeutically during millennia for a broad range of biological activities^36–38^. Nowadays, EOs, their components and derivatives thereof, are used in a wide variety of commercial applications. They are found in numerous products, including regular medicines^39–43^; and for some of these, randomized controlled (clinical) trials have been performed^44–47^. An EO is defined by the International Organization for Standardization as a product obtained from raw plant material by water, steam or dry distillation, or by mechanical processing of the epicarp of citrus fruits, after possible separation of the aqueous phase by physical processes. In addition, an EO may undergo physical treatments, e.g. filtration, decantation, centrifugation, provided this does not significantly change its composition^48^. Although other EO definitions are used, this study adheres to the International Organization for Standardization definition, because it permits a clear distinction between EOs and EO-like products, such as (supercritical fluid) extracts^36,48–50^. Plant species, - chemotype, - part(s) and extraction method have a major influence on the composition of an EO. EOs are typically composed of many essential oil components (EOCs), most of which are synthesized via the methylerythritol phosphate -, mevalonic acid -, or shikimate pathway^36,51^.

Natural products such as EOs are often avoided in drug discovery, partly because of some undesirable characteristics^1,4^. EOs are complex mixtures of relatively hydrophobic, volatile EOCs, which can cause interference during screening^52–54^. However, can the exclusion of EO(C)s in the search for new drugs be justified because of these properties or because they are (derivatives of) natural products? To answer this question, DDPs of EOCs obtained from a set of commercially available EOs were calculated, analyzed and summarized. Then the lead- and drug-likeness of these EOCs were evaluated using all the DDFs available in JChem (for Office) from ChemAxon, a widely accepted cheminformatics software package in drug discovery (Table 1). Additionally, the Rule of Three (Ro3) DDF was used to test if some EOCs would be potential candidates for fragment-based drug discovery^24,25,55^. Finally, the results of the DDFs obtained from (i) the EOCs in our EO set and (ii) the approved drugs in DrugBank were compared.

## Results

### Selection of an EO set

A sample of 188 chemotypically defined EOs, representing a cross-section of what is currently commercially available, were obtained from Pranarôm International S.A. (Belgium). Eliminating duplicates led to our final set of 175 EO (SI 1), on which all further analyses were performed^54,56^. These EOs were produced by distillation (93.7%) or by mechanical pressing (6.3%)^48^.

### Analysis of EOCs: introducing the (unique) Core Molecular Constitution of EOCs: (u-)cmcEOCs

EO analysis by Gas Chromatography with Mass Spectrometry and Flame-Ionization Detection (GC-MS-FID) identified a total of 6,142 EOCs (≥0.10%; n_EO_ = 175), at least at the level of their Core Molecular Constitution (CMC; Box 1); they are further referred to as cmcEOCs and were retained for further analysis. A total of 764 EOCs (≥0.10%; n_EO_ = 175) could not be identified at least at their CMC level, resulting in incomplete or no data on the DDPs being studied; therefore, they were not included in further analyses.

**Figure 1:**
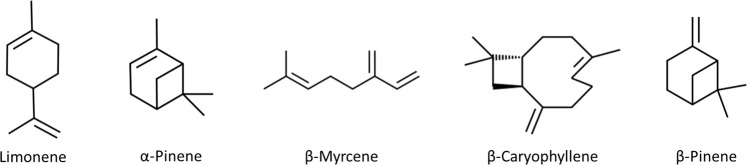
Chemical structures of the five most frequently found u-cmcEOCs in our EO set (in descending order of frequency from left to right).

Because there is an overlap in composition between EOs, many of the 6,142 cmcEOCs are identical, and the whole set can be described with only 627 different InChIKeys-14; these are further referred to as unique-cmcEOCs (u-cmcEOCs; Box 2; SI 2). Approximately 35% (n = 218) of the u-cmcEOCs appear only in one EO. This is in strong contrast with the five most frequently found u-cmcEOCs in our EO set (Fig. 1), i.e. limonene (identified in 153 EOs: n_EO_ = 153; see also SI 2 no 551), alpha-pinene (n_EO_ = 149; SI 2 no 137), beta-myrcene (n_EO_ = 141; SI 2 no 457), beta-caryophyllene (n_EO_ = 139; SI 2 no 319) and beta-pinene (n_EO_ = 133; SI 2 no 523). Together, these results largely correspond with previous findings^57^. As a robustness check, the EOC composition of three subsets (A-C) of our EO set were analyzed, i.e. (A) EOs of conventional cultivation (n = 101), (B) EOs of certified-organic cultivation (n = 74), and (C) all EOs of conventional cultivation, complemented with those EOs of certified-organic cultivation that originated from other plant species, the same plant species but from other plant parts, or the same plant species but a different chemotype (n = 141; SI 1). No differences in rank order were found for the five most frequent u-cmcEOCs for the defined EO (sub)sets (SI 3). This indicates that a typical EO consists of a combination of common and rare EOCs, and these findings suggest that the most common EOCs are present in a majority of EOs.

Box 1 The **Core Molecular Constitution (CMC)** of a molecule describes *i.a.* its chemical formula, connectivity and hydrogen positions^83^. It is encoded by the first block of 14 (out of 27 total) characters of an InChIKey, subsequently referred to as **InChIKey-14**.

For example, EOCs trans-α-bisabolene (InChIKey-14 = YHBUQBJHSRGZNF), α-bisabolene (InChIKey-14 = YHBUQBJHSRGZNF), β-bisabolene (InChIKey-14 = XZRVRYFILCSYSP) and (-bisabolene (InChIKey-14 = XBGUIVFBMBVUEG) are all identified, at least at their CMC. The InChIKey-14 for the first two EOCs are the same, as stereochemical information like geometric isomerism is not part of the CMC. This is because information on isomerism is not encoded in the first -, but in the second block of the InChIKey, and is indeed different for all four aforesaid EOCs (data not shown). In contrast, for an EOC identified at a lower level of granularity, e.g. bisabolene without α-, β- or (-preposition, no InChIKey exists as bisabolene refers to several closely related compounds with a different CMC.

Box 2 **u-cmcEOC** is an essential oil component (EOC) identified at its core molecular constitution (CMC) after deduplication of the EOCs in an EO set based on their InChIKeys-14 (see also Box 1).

By analogy, **u-cmcAD** is an Approved Drug (AD) identified at its CMC after deduplication of the ADs in DrugBank based on their InChIKeys-14.

### Suitability of EOCs for drug discovery and development: DDPs

**Figure 2:**
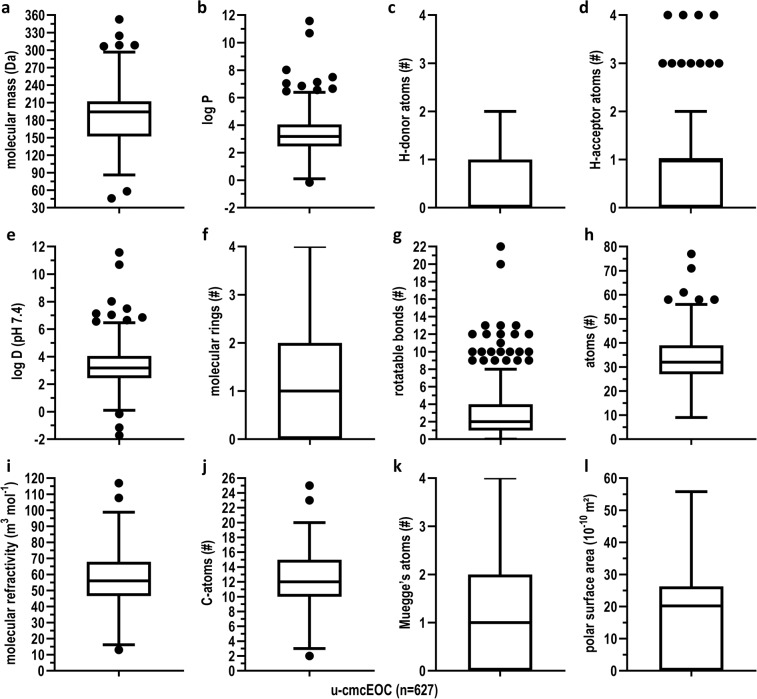
Summary of the values for the u-cmcEOCs (n = 627; Box 2) of the DDPs in the DDFs we used. Tukey boxplots of the DDP values for the u-cmcEOCs [minimum; 25th percentile; median; 75th percentile; maximum] (unit of measurement): (**a**) molecular mass [46.1; 152.2; 194.2; 212.4; 352.7] (Da), (**b**) log P [−0.16; 2.46; 3.18; 4.06; 11.58], (**c**) number of H-donor atoms [0; 0; 0; 1; 2], (**d**) number of H-acceptor atoms [0; 0; 1; 1; 4], (**e**) log D at pH 7.4 [−1.71; 2.44; 3.18; 4.06; 11.58], (**f**) number of molecular rings [0; 0; 1; 2; 4], (**g**) number of rotatable bonds [0; 1; 2; 4; 22], (**h**) number of atoms [9; 27; 32; 39; 77], (**i**) molecular refractivity [13.01; 46.50; 56.01; 67.90; 116.80] (m^3^ mol^−1^), (**j**) number of C-atoms [2; 10; 12; 15; 25], (**k**) Muegge’s atoms [0; 0; 1; 2; 4], (**l**) polar surface area [0.00; 0.00; 20.23; 26.30; 55.76] (10^−10^ m^2^). Values for the DDP fused aromatic rings were not summarized as for all but four u-cmcEOCs the values were zero. For more details on the Drug Discovery Parameters described in b, e, i, k, l, see the materials and methods section.

DDPs are (derivatives of) common physicochemical parameters. DDFs may use the same DDPs, but possibly with different value ranges. To evaluate the potential lead- and drug-likeness of the u-cmcEOCs present in our EO set, we calculated the values of 13 DDPs, and determined how many times these satisfy the criteria for one or more of the six standard DDFs in our study (Fig. 2 and Table 2). We also demonstrate that some DDPs, e.g. log P and log D (pH 7.4) or Muegge’s atoms and polar surface area, are unsurprisingly very highly correlated and may be interchangeable (SI 4).

**Table 2:** Number (percentage) of u-cmcEOCs (Box 1) that pass each criterion separately (Drug Discovery Parameters; top rows). Number (percentage) of u-cmcEOCs and u-cmcADs (Box 2) that pass the combined criteria of a specific Drug Discovery Filter (bottom rows) available in JChem (for Office)^21,22,56–58^. —: not applicable.

Drug Discovery Parameter (n_total_ = 627)	Drug Discovery Filter					
	Ro5 all 4 criteria	Lead Likeness	Ghose	Muegge	Veber	Bio-availability ≥ 6 criteria
molecular mass	n = 627 (100%)	n = 627 (100%)	n = 396 (63.2%)	n = 283 (45.1%)	—	n = 627 (100%)
log P^&^	n = 591 (94.3%)	n = 544 (86.8%)^£^	n = 608 (97.0%)	n = 591 (94.3%)	—	n = 591 (94.3%)
H-bond donors	n = 627 (100%)	n = 627 (100%)	—	n = 627 (100%)	n = 627 (100%)^§^	n = 627 (100%)
H-bond acceptors	n = 627 (100%)	n = 627 (100%)	—	n = 627 (100%)		n = 627 (100%)
log D (pH 7.4)^&^	—	n = 457 (72.9%)^£^	—	—	—	—
molecular rings	—	n = 627 (100%)	—	n = 627 (100%)	—	—
rotatable bonds	—	n = 616 (98.2%)	—	n = 625 (99.9%)	n = 616 (98.2%)	n = 616 (98.2%)
Atoms	—	—	n = 593 (94.6%)	—	—	—
molar refractivity^&^	—	—	n = 581 (92.7%)	—	—	—
C-atoms	—	—	—	n = 624 (99.5%)	—	—
Muegge’s atoms^&^	—	—	—	n = 204 (32.5%)	—	—
polar surface area^&^	—	—	—	n = 627 (100%)	n = 627 (100%)^§^	n = 627 (100%)
fused aromatic rings	—	—	—	—	—	n = 627 (100%)
u-cmcEOCs passing Drug Discovery Filter (n_total_ = 627)	**n = 591 (94.3%)**	**n = 456 (72.7%)**	**n = 377 (60.1%)**	**n = 59 (9.4%)**	**n = 616 (98.2%)**	**n = 616 (98.2%)**
u-cmcADs passing Drug Discovery Filter (n_total_ = 2,359)	**n = 1,744 (73.9%)**	**n = 1,401 (59.4%)**	**n = 1,129 (47.9%)**	**n = 1,226 (52.0%)**	**n = 1,840 (78.0%)**	**n = 1,987 (84.2%)**

^&^For more details on the Drug Discovery Parameters, see the materials and methods section. ^£,§^The Lead Likeness and Veber Drug Discovery Filters can alternatively use one or the other criterion^22,57^: For the Lead Likeness and Veber Drug Discovery Filter, log D (pH 7.4) and polar surface, respectively, are considered the standard Drug Discovery Parameters.

More than 90% of the u-cmcEOCs had values within the criteria limits for at least nine out of 13 DDPs, irrespective of the DDF. For only two DDP criteria of one DDF, i.e. the Muegge filter, more than half of the values of the u-cmcEOCs DDPs were outside the limits for these criteria (Table 2).

### Suitability of EOCs for drug discovery and development: DDFs

All u-cmcEOCs were evaluated with the DDFs available in JChem (for Office), i.e. (i) Ro5, (ii) Lead Likeness, (iii) Ghose, (iv) Muegge, (v) Veber, and (vi) Bioavailability, thus combining all the DDP criteria of each DDF, including three variants (Table 1), and the Ro3 DDF. A u-cmcEOC passes a DDF if the u-cmcEOC DDP values for all criteria of that DDF are within the DDP limits.

#### DDFs for bioavailability

For the Ro5 benchmark DDF, all (n = 627) u-cmcEOCs passed the Ro5 DDF when assumed that an orally active drug usually has no more than one criterion violation^21^, and more than 94% (n = 591) of the u-cmcEOCs passed all four criteria (Tables 1 and 2). According to Lipinski, candidate drugs that meet the Ro5 criteria tend to have a lower attrition rate during clinical development, and therefore have an increased chance of reaching the market^29,58^. Approximately 98% (n = 607) of the u-cmcEOCs passed the Veber DDF, which again implies that u-cmcEOCs generally should have good oral bioavailability assuring good intestinal absorption^22,59^. Veber *et al*.^22^ reported that the DDPs polar surface area and rotatable bonds probably discriminated better than DDP molecular mass between compounds that are orally bioavailable, and those which are not^22^. For our sample, we could not prove that DDPs polar surface area and molecular mass are correlated (ρ = 0.07, p > 0.05). We found that DDPs rotatable bonds and molecular mass are very weakly correlated (ρ = 0.10; p < 0.05) indicating that the Veber DDF captures mostly different information compared to the Ro5 DDF (SI 4). Furthermore, because the DDPs polar surface area and rotatable bonds are only moderately correlated (ρ = 0.46; p < 0.0001), these DDPs capture partially different information (SI 4). Therefore, the DDFs Ro5 and Veber can be considered complementary DDFs, at least for our EO set. Also, approximately 98% (n = 607) of the u-cmcEOCs passed the Bioavailability DDF (Table 2). This is not entirely surprising, because the Bioavailability DDF is essentially the merging of the Ro5 and Veber DDFs, complemented with the fused aromatic rings DDP, and whereby for this filter only any of 6 out of 7 criteria must be met (Table 1).

#### DDF for lead-likeness

DDFs are most often applied to hits from high throughput screens. However, in order to improve affinity and selectivity of a drug candidate, additional chemical groups are usually added, so that molecular mass and lipophilicity often increase during lead optimization. The Lead Likeness DDF, for example, is biased towards lower lipophilicity and molecular mass, so that interesting lead candidates can be further optimized towards drug-like candidates (Table 1). The standard Lead Likeness DDF uses the DDPs Log D at pH 7.4 or alternatively Log P; approximately 73% (Table 2) or 87% (n = 544; not shown in Table 2), respectively, of the u-cmcEOCs pass this DDF.

#### DDF for fragment-based drug discovery

Furthermore, a DDF derived from Ro5 appears useful for efficient lead discovery in a fragment-based drug discovery approach, i.e. the Ro3 DDF^24^. About 32% (n = 202) of the u-cmcEOCs pass all 5 DDP criteria of the Ro3 (for criteria see Materials and Methods section). Most u-cmcEOCs that do not pass this DDF fail because the criteria limit(s) of DDP log P and/or DDP rotatable bonds were exceeded in 55.5% (n = 348) and 32.7% (n = 205) of the cases, respectively.

#### DDFs for drug-likeness

The DDP criteria of the high drug-likeness Ghose DDF are based on an analysis of known drugs from the Comprehensive Medicinal Chemistry database^60^ and approximately 60% (n = 377) of the u-cmcEOCs passed this DDF (Table 2). In contrast, less than 10% (n = 59) of the u-cmcEOs passed the Muegge filter, which tries to differentiate between drug-like and non-drugs based on the observation that non-drugs are often under-functionalized^61^. The reasons for failing the Ghose or Muegge DDFs were the lower limit of DDP molecular mass for both DDFs, combined with the DDP Muegge’s atoms (i.e. the total number of atoms of a molecule minus the total number of carbon and hydrogen atoms) for the latter (Table 1). Only about 32% of the u-cmcEOCs passed Muegge’s atoms criterion (Table 2).

In general, only nine out of 627 u-cmcEOCs (SI 2; nos. 33, 99, 159, 165, 226, 233, 251, 571, 584) did not pass any of the DDFs under study, including variants, with the exception of the DDF Ro5 variant, where only three out of four criteria suffice, so that all u-cmcEOCs pass this latter DDF variant. In contrast, eight u-cmcEOCs (SI 2; nos. 8, 73, 371, 407, 490, 510, 543 and 560) passed all ten DDFs (variants) in our study, implying that these u-cmcEOCs passed the most stringent criteria of each DDP in our study.

Box **Taken together, we can conclude that most u-cmcEOCs are promising lead-like and/or drug-like candidates, and that some EOCs could be candidates for fragment-based drug discovery.**

### Comparing EOCs in our sample with approved drugs in DrugBank

The results of the standard DDF analyses of the u-cmcEOCs in our sample and the u-cmcADs (Box 2) in DrugBank were compared (Tables 2 and 3). Except for the Muegge DDF, proportionally more u-cmcEOCs than u-cmcAD passed the individual standard DDFs (Table 2), indicating that overall, EOCs meet the combined criteria of most DDFs at least as well as the drugs on the market. However, proportionally about six times more approved drugs (35.4%) passed all the DDFs compared to the u-cmcEOCs (6.2%). Nonetheless, about 94% of all u-cmcEOCs passed at least four out of six DDFs compared with only 67.5% of the approved drugs. It should be noted that a relatively large proportion of the approved drugs (13.8%) did not pass any of the DDFs (Table 3).

**Table 3:** Number (percentage) of u-cmcEOCs and u-cmcADs (Box 2) that pass exactly X standard Drug Discovery Filters (with 0 ≤ X ≤ 6).

	pass any X Drug Discovery Filters out of six						
	X = 6	X = 5	X = 4	X = 3	X = 2	X = 1	X = 0
u-cmcEOCs (n_total_ = 627)	n = 39 (6.2%)	n = 208 (33.1%)	n = 342 (54.5%)	n = 17 (2.7%)	n = 10 (1.6%)	n = 2 (0.3%)	n = 9 (1.4%)
u-cmcADs (n_total_ = 2,359)	n = 836 (35.4%)	n = 270 (11.4%)	n = 489 (20.7%)	n = 205 (8.7%)	n = 156 (6.6%)	n = 78 (3.3%)	n = 325 (13.8%)

### Some EO(C)s made it into DrugBank

DrugBank lists few u-cmcEOCs approved as drugs in at least one jurisdiction (SI 5). Eugenol for instance found routine use as a topical antiseptic in dentistry, as a counter-irritant and for pain control; it is the major EOC of the EO *Syzygium aromaticum*, also known as *Eugenia caryophyllus*. Menthol is used as a local anesthetic, has counter-irritant qualities, and relieves minor throat irritation; it is a major EOC of EO *Mentha* x *piperita*^62^. Moreover, none of the EOCs approved as drugs have ever been withdrawn (SI 5), though a limited number of u-cmcADs (n = 138; 5.8%) have been withdrawn to date. However, it is difficult to draw conclusions from this because the sample of EOCs approved as drugs is too small. We can, nevertheless, conclude that, once approved as a drug, EOCs have stood the test of time. In addition, of the 180 approved drugs in DrugBank for which no InChIKey could be defined, e.g. because they were (among others) complex mixtures of components, we identified at least seven (EOs of Eucalyptus, turpentine, sage, tea tree, Pinus mugo (needle), Curcuma aromatica (root), Atractylodes japonica (root).) EOs that are approved as drugs. None of these EOs have been withdrawn to date, although this does not imply their efficacy or lack of toxicity.

## Discussion

A diverse set of 175 commercially available and chemically defined EOs from a multinational company specialized in scientific aromatherapy was selected for analysis. Possible advantages are that (i) they must meet a minimum number of (quality) requirements, and (ii) when they are purchased from the same reliable source, analysis procedures and handling of the EOs have been standardized where possible, hence minimizing variation. A possible disadvantage is selection bias i.e. the exclusion of non-commercial EOs, or bias against some EOs because they are e.g. toxic, insufficiently marketed, locally regulated, not available in sufficient quantities, or considered insufficiently interesting from the company’s viewpoint. The quality control parameters of our set of commercial EOs (SI 6) coincide with previous findings where no distinction was made between commercial and non-commercial EOs^36,49,63–65^. From a drug discovery perspective, however, the investigation of non-commercial, uncommon and toxic EOs merits attention as they are potentially an interesting source of possibly unknown and rare lead- and drug-like EOCs.

We found it useful to define the CMC of an EOC as its InChIKey-14 (Box 1); this permitted e.g. rapid and effective deduplication. This CMC contains in a coded manner essential information about the structure and composition of a molecule, but without data on isomerism (which are encoded by the remainder of the InChIKey). We are not aware of earlier uses of the CMC as we defined it, and believe that it may be useful for the chemoinformatic analysis of other compounds.

In this study, each component with an InChIKey-14 (n = 627) was considered an EOC. Two of these InChIKeys-14, however, belong to molecules that are not considered EOCs, but are commonly found in the EOs due to procedural contamination or fermentation, e.g. acetone (SI 2; no. 58) and ethanol (SI 2; no. 254). However, eliminating these two molecules would not have influenced our conclusions as their number is small (0.32%) compared to the entire sample.

The Muegge DDF has two DDPs that u-cmcEOCs rarely satisfy: the atom criterion and the lower limit on molecular mass. (i) Muegge’s atoms criterion requires that the total number of atoms of a molecule minus the total number of carbon and hydrogen atoms be equal to or greater than 2 (Table 1). Because all u-cmcEOCs except for three (<0.5%) nitrogen-containing ones (SI 2; nos. 139, 455, 481) consist of only carbon, hydrogen and oxygen atoms, u-cmcEOC with no or only one oxygen atom would therefore not meet this Muegge criterion (67.5%; Table 2). This would include all but four (SI 2; nos. 29, 346, 415 and 428) of the above-mentioned EOCs that are approved as drugs (SI 5). (ii) The lower limit of the DDP molecular mass (≥200; Table 1). Almost 55% of the u-cmcEOCs in our sample do not meet this criterion (Table 2), including all but two (SI 2; nos. 403 and 428) of the above-mentioned EOCs that are approved as drugs (SI 5). None of the EOCs in our sample that were ever approved as drugs (n = 12), according to DrugBank, were ever withdrawn, and only one, i.e. benzyl benzoate (SI 2; no. 428), met all criteria of the Muegge DDF^62^. Moreover, the Muegge atoms criterion is based on the observation that drugs have on average more “pharmacophore points” (i.e. functional groups such as amine, amide, alcohol, ketone, sulfone, sulfonamide, carboxylic acid, carbamate, guanidine, amidine, urea, and ester) than non-drugs^61^. But Muegge admits that 30–40% of compounds in different drug databases do not meet this criterion, whereas over one-third of non-drug chemicals do. In isolation, this criterion therefore does not discriminate drugs from non-drugs very well. The requirement for multiple pharmacophore points fits with the idea that these groups confer binding specificity. Although the clinical effects of EO(C)s often suggest sufficient therapeutic specificity, functional groups could be added to lead-like EOCs to increase their specificity for the desired molecular target. As these groups will also increase the molecular mass, this will also help to satisfy the second of Muegge’s DDPs. Since most EOCs tend to be small, they provide ample room for adding functional groups before running foul of other DDP criteria based on molecule size. Therefore, we think that the Muegge DDF should not be a limiting filter for the evaluation of EOCs.

The fact that relatively few EO(C)s made it into approved drugs could be due to their unusual properties. However, when these properties are benchmarked against various measures of drug-likeness, most EOCs pass with flying colors. For example, all u-cmcEOCs passed the Ro5 when only three of the four criteria had to be met. In addition, almost 94% of the EOCs passed at least any four out of six standard DDFs. Because DDFs are based on marketed drugs, it was expected that many approved drugs in DrugBank would pass most, but not necessarily all DDFs. The Lead Likeness and Ro3 DDFs, for example, were developed to search for lead molecules, and therefore not necessarily for marketed drugs that have already passed this development stage. Conversely, it was expected that most u-cmcEOCs would not pass all six DDFs.

One of the major drawbacks, however, in the transition of a natural compound from a hit to a drug is the increased amount of compound required, which often cannot be met by re-isolation from the relevant plant sources^34,66^. EO production and distribution, however, is a mature industry and EOs were the 446^st^ most-traded product in the world in 2017, with a total export value of 5.44 billion $ (SI 7)^67^. Therefore, if necessary, EO production can be relatively easily scaled up, with or without the use of biotechnology^68^, while medicinal chemists^69^ can find a way to synthesize the EOC and derivatives thereof^70^.

In the end, this suggests that EOCs, are promising (sources of) new drugs and deserve more attention in the future. EOCs also have unique properties that might be useful for some therapeutic applications, e.g. for lung or airway diseases^71–74^, for transdermal administration^75,76^ and diseases of the central nervous system^77–79^.

## Materials and Methods

### Essential oils (EOs)

A set of 175 EOs, representing a cross-section of what is currently commercially available, were retained for further data analysis from a sample of 188 chemotypically defined EOs, obtained from Pranarôm International S.A. (Belgium). Chemical composition, quality and origin of the EOs were certified by the company. The reduction from 188 EOs to our final set of 175 is essentially due to deduplication^54^. EOs were considered different when originating from (i) other plant species, (ii) the same plant species but from other plant parts, (iii) the same plant species but a different chemotype, and (iv) certified-organic versus conventional cultivation. Chemical analyses of the EOs were performed by GC-MS-FID using the NF ISO 11024-1/2 standard (Pranarôm International S.A., personal communication). The detection of organophosphorus and organochlorine pesticides residue levels was in compliance with the relevant EU-legislation and maximum permitted levels were never exceeded^56^. The chemical composition (≥10%) and metadata of the EOs used in this study was reported previously; see www.nature.com/articles/s41598–018–22395–6 under the heading electronic supplementary material^54^. More detailed analysis certificates and the methodology used (in French) can be consulted at www.inula-group.com/fr/pranaquality (see also SI 1).

### Data preparation, calculations and visualisation of the EO set

Initially all GC-FID peaks ≥ 0.01% from the EO set (n = 175) were considered. After a preliminary evaluation, only peaks ≥ 0.10% were retained for further analysis because many GC-peaks < 0.10% were not or only partially identifiable. Subsequently, any EOC that was at least identifiable at its core molecular constitution (CMC; see also Box 1) was retained for further analysis, and a standard International Chemical Identifier (InChI) along with the corresponding hashed 27-character counterpart, i.e. InChIKey, was assigned using publicly accessible databases e.g. ChemSpider, PubChem or Chemistry WebBook^80,81^. Only the first 14 characters of the InChIKeys (InChIKeys-14) were retained of each EOC, thereby removing additional layers of information other than the CMC of the EOC (cmcEOC). After deduplication of the cmcEOCs (u-cmcEOCs), the unique InChIkeys-14 of the u-cmcEOCs were retained for further analysis. To display with Marvin the u-cmcEOCs molecular structures (SI 2), a structure-data file was created with ChemMine Tools using the u-cmcEOCs Simplified Molecular-Input Line-Entry System, a.k.a. SMILES, notation. To this end, the unique InChIkeys-14 was first complemented with an information-neutral second InChIKey block, i.e. UHFFFAOYSA, to re-establish a full InChIKey that was then translated with JChem (for Office) into SMILES^81,82^. RapidMinerStudio was used for data preparation and data blending.

### Data preparation of, and calculations on, the DrugBank sample

The CSV-file (n_entries_ = 2,594) ‘approved’ in the ‘drug group’ column was downloaded from DrugBank containing the names of all drugs that were once approved in any jurisdiction at any given time, and the structure information in the form of, e.g. InChI/InChI Key/SMILES for most of them (n_entries_ = 2,414). All approved drugs with no structure information (n_entries_ = 180) were initially not considered and therefore removed from the sample. Subsequently, the second block of the InChIKeys was removed, resulting in an InChIKey-14 for each drug. After deduplication, a total of 2,359 unique InChIKeys-14, corresponding to the unique CMCs (Box 1), of all approved drugs (u-cmcADs) in DrugBank were retained for further analysis.

### Drug Discovery Parameters (DDPs)

JChem (for Office) and Excel were used for (i) chemical database access, (ii) structure-based property calculations (Fig. 2 and Table 2) and (iii) for searching and reporting the chemical structures i.e. u-cmcEOCs (SI 2). Briefly, to estimate the octanol/water partition and distribution coefficients of the EOCs, the consensus model of ChemAxon, based on the Viswanathan *et al*.^83^ and Klopman *et al*.^84^ models, and the PhysProp database^85,86^, were used by JChem (for Office)^87^. For calculating the water/octanol partition coefficient, P, only the un-ionized form was considered, whereas the distribution coefficient, D, also considers, if applicable, all charged forms of the molecule for a given pH; thus we obtained DDPs (i) Log P = log_10_(octanol/water partition coefficient) and (ii) Log D = log_10_(octanol/water distribution coefficient). (iii) The molar refractivity was calculated based on the atomic method described by Viswanadhan *et al*.^83^ and to estimate (iv) the polar surface area of the EOCs, the topological polar surface area method as described by Ertl *et al*.^88^ was used by JChem (for Office)^88^. (v) Muegge’s atoms DDP is equal to total number of atoms of a molecule minus the total number of carbon and hydrogen atoms.

### Drug Discovery Filters (DDFs)

JChem (for Office) and Excel were used for calculating the number of u-cmcEOCs and u-cmcADs that passed the different DDFs. The six DDFs supported by JChem (for Office) are referred to as standard DDFs (Tables 1 and 2). Three of the six standard DDFs each have two variants: (i) for the Ro5 DDF, three or four out of four criteria have to be met to pass this filter, and the latter more conservative variant was considered standard^87^. For the (ii) Lead Likeness and (iii) Veber DDFs, the combinations of DDPs supported by JChem (for Office) were considered standard, whereas the alternative combination of DDPs mentioned in the respective publications were considered non-standard variants (see also Tables 1 and 2)^22,87,89^. We added one DDF not included in JChem (for Office); it is derived from the Ro5 (i.e. the Ro3 DDF with the following DDPs: (i) log P ≤ 3, (ii) molecular mass ≤ 300, (iii) hydrogen bond donors ≤ 3, (iv) hydrogen bond acceptors ≤ 3 and (v) rotatable bonds ≤ 3)^24^. In all, we use 10 DDF(s) (variants) i.e. Ro5 (2 variants), Lead Likeness (2 variants), Ghose, Muegge, Veber (2 variants), Bioavailability, and Ro3.

### Statistical analyses

Statistical analyses were performed using GraphPad Prism. For correlation analyses, the Spearman rank correlation coefficient (ρ) was calculated.

### Software versions & databases

We used GraphPad Prism versions 7.0.5–8.1.2 (www.graphpad.com), JChem for Office versions 17.22–19.14 and Marvin versions 18.16–19.14 (ChemAxon), Office 365 ProPlus (Microsoft), RapidMinerStudio versions 9.0–9.5 (RapidMiner) and ChemMine Tools (Girke Lab)^82^. The databases ChemSpider (Royal Society of Chemistry), PubChem (National Institutes of Health), Chemistry WebBook (National Institute of Standards and Technology) and Drugbank version 5.1.4 (www.drugbank.ca)^62^ were accessed between August 2017 and July 2019.

## Supplementary information

Supplementary information.

## References

[1] Li JW, Vederas JC (2009). Drug discovery and natural products: end of an era or an endless frontier?. Sci..

[2] Rishton GM (2008). Natural products as a robust source of new drugs and drug leads: past successes and present day issues. Am. J. Cardiol..

[3] Secretariat of the Convention on Biological Diversity (SCBD). *The Nagoya protocol on access and benefit-sharing*, https://www.cbd.int/abs/ (2018).

[4] Harvey AL, Edrada-Ebel R, Quinn RJ (2015). The re-emergence of natural products for drug discovery in the genomics era. Nat. reviews. Drug. discovery.

[5] Charlotte H (2014). Patenting natural products just got harder. Nat. Biotechnol..

[6] Kartal M (2007). Intellectual property protection in the natural product drug discovery, traditional herbal medicine and herbal medicinal products. Phytotherapy research: PTR.

[7] Shen B (2015). A new golden age of natural products drug discovery. Cell.

[8] Grabowski K, Baringhaus KH, Schneider G (2008). Scaffold diversity of natural products: inspiration for combinatorial library design. Nat. Prod. Rep..

[9] Drewry DH, Macarron R (2010). Enhancements of screening collections to address areas of unmet medical need: an industry perspective. Curr. Opin. Chem. Biol..

[10] Rosen J, Gottfries J, Muresan S, Backlund A, Oprea TI (2009). Novel chemical space exploration via natural products. J. medicinal Chem..

[11] Bauer RA, Wurst JM, Tan DS (2010). Expanding the range of ‘druggable’ targets with natural product-based libraries: an academic perspective. Curr. Opin. Chem. Biol..

[12] Wetzel S, Bon RS, Kumar K, Waldmann H (2011). Biology-oriented synthesis. Angew. Chem. Int. Ed. Engl..

[13] Lachance H, Wetzel S, Kumar K, Waldmann H (2012). Charting, navigating, and populating natural product chemical space for drug discovery. J. medicinal Chem..

[14] Dias DA, Urban S, Roessner U (2012). A historical overview of natural products in drug discovery. Metabolites.

[15] Newman DJ, Cragg GM (2012). Natural products as sources of new drugs over the 30 years from 1981 to 2010. J. Nat. Prod..

[16] Newman DJ, Cragg GM (2016). Natural products as sources of new drugs from 1981 to 2014. J. Nat. Prod..

[17] Pitterna T (2009). New ventures in the chemistry of avermectins. Bioorganic & medicinal Chem..

[18] Vallianatou T, Giaginis C, Tsantili-Kakoulidou A (2015). The impact of physicochemical and molecular properties in drug design: navigation in the “drug-like” chemical space. Adv. Exp. Med. Biol..

[19] Amirkia V, Heinrich M (2015). Natural products and drug discovery: a survey of stakeholders in industry and academia. Front. pharmacology.

[20] Alteri E, Guizzaro L (2018). Be open about drug failures to speed up research. Nat..

[21] Lipinski CA, Lombardo F, Dominy BW, Feeney PJ (2001). Experimental and computational approaches to estimate solubility and permeability in drug discovery and development settings. Adv. Drug. Delivery Rev..

[22] Veber DF (2002). Molecular properties that influence the oral bioavailability of drug candidates. J. medicinal Chem..

[23] Muegge I (2003). Selection criteria for drug-like compounds. Medicinal Res. Rev..

[24] Congreve M, Carr R, Murray C, Jhoti H (2003). A ‘rule of three’ for fragment-based lead discovery?. Drug. Discov. Today.

[25] Murray CW, Rees DC (2009). The rise of fragment-based drug discovery. Nat. Chem..

[26] Kola I, Landis J (2004). Can the pharmaceutical industry reduce attrition rates?. Nat. Rev. Drug. Discovery.

[27] Waring MJ (2015). An analysis of the attrition of drug candidates from four major pharmaceutical companies. Nat. reviews. Drug. discovery.

[28] Barker A, Kettle JG, Nowak T, Pease JE (2013). Expanding medicinal chemistry space. Drug. Discovery Today.

[29] Lipinski CA (2004). Lead- and drug-like compounds: the rule-of-five revolution. Drug. Discov. Today Technol..

[30] Lipinski CA (2016). Rule of five in 2015 and beyond: Target and ligand structural limitations, ligand chemistry structure and drug discovery project decisions. Adv. Drug. Deliv. Rev..

[31] Gu J (2013). Use of natural products as chemical library for drug discovery and network pharmacology. PLoS one.

[32] Maplestone RA, Stone MJ, Williams DH (1992). The evolutionary role of secondary metabolites–a review. Gene.

[33] Zhang MQ, Wilkinson B (2007). Drug discovery beyond the ‘rule-of-five’. Curr. Opin. Biotechnol..

[34] Atanasov AG (2015). Discovery and resupply of pharmacologically active plant-derived natural products: A review. Biotechnol. Adv..

[35] Moloney MG (2016). Natural products as a source for novel antibiotics. Trends Pharmacol. Sci..

[36] Franchomme, P., Jollois, R., Pénoël, D. & Mars, J. L’aromathérapie exactement: encyclopédie de l’utilisation thérapeutique des huiles essentielles: fondements, démonstration, illustration et applications d’une science médicale naturelle. (R. Jollois, 2001).

[37] Bakkali F, Averbeck S, Averbeck D, Idaomar M (2008). Biological effects of essential oils - a review. Food Chem. toxicology: an Int. J. published Br. Ind. Biol. Res. Assoc..

[38] Brown ME (2014). Survivors over six millennia: essential oils. Pharm. historian.

[39] PDQ® integrative, a., and complementary therapies editorial board. Aromatherapy and essential oils (PDQ®): Health professional version. (National Cancer Institute, Bethesda, MD, US, 2005–2018).

[40] PDQ® integrative, a., and complementary therapies editorial board. Aromatherapy and essential oils (PDQ®): Patient version. (National Cancer Institute, Bethesda, MD, US, 2007–2018).

[41] Van Bortel LM, Petrovic M, De Paepe P (2014). Farmacotherapeutische actualiteit: nieuwe geneesmiddelen. Tijdschr. voor geneeskunde.

[42] Dhifi, W., Bellili, S., Jazi, S., Bahloul, N. & Mnif, W. Essential oils’ chemical characterization and investigation of some biological activities: A critical review. Medicines (Basel, Switzerland) **3**, 10.3390/medicines3040025 (2016). 10.3390/medicines3040025 PMC5456241 28930135

[43] Nagoor Meeran MF, Javed H, Al Taee H, Azimullah S, Ojha SK (2017). Pharmacological properties and molecular mechanisms of thymol: Prospects for Its therapeutic potential and pharmaceutical development. Front. Pharmacol..

[44] Nakayama M, Okizaki A, Takahashi K (2016). A randomized controlled trial for the effectiveness of aromatherapy in decreasing salivary gland damage following radioactive iodine therapy for differentiated thyroid cancer. BioMed. Res. Int..

[45] Hekmatpou D, Pourandish Y, Farahani PV, Parvizrad R (2017). The effect of aromatherapy with the essential oil of orange on pain and vital signs of patients with fractured limbs admitted to the emergency ward: A randomized clinical trial. Indian. J. Palliat. care.

[46] Dimitriou V, Mavridou P, Manataki A, Damigos D (2017). The use of aromatherapy for postoperative pain management: A systematic review of randomized controlled trials. J. PeriAnesthesia Nurs..

[47] Nasiri A, Mahmodi MA (2018). Aromatherapy massage with lavender essential oil and the prevention of disability in ADL in patients with osteoarthritis of the knee: A randomized controlled clinical trial. Complement. Ther. Clin. Pract..

[48] International Organization for Standardization. 9235:2013: Aromatic natural raw materials. (Geneva, Switzerland, 2013).

[49] Baser, K. H. C., Buchbauer, G. & Editors. Handbook of essential oils: science, technology, and applications. 2 edn, (CRC Press, 2016).

[50] Essential oil. (Encyclopædia Britannica, Inc., 2018).

[51] Livermore DM (2011). Discovery research: the scientific challenge of finding new antibiotics. J. antimicrobial chemotherapy.

[52] Novy P (2014). Thymoquinone vapor significantly affects the results of Staphylococcus aureus sensitivity tests using the standard broth microdilution method. Fitoterapia.

[53] Feyaerts AF (2017). Assay and recommendations for the detection of vapour-phase-mediated antimicrobial activities. Flavour. Fragr. J..

[54] Feyaerts AF (2018). Essential oils and their components are a class of antifungals with potent vapour-phase-mediated anti-Candida activity. Sci. Rep..

[55] Jhoti H, Williams G, Rees DC, Murray CW (2013). The ‘rule of three’; for fragment-based drug discovery: where are we now?. Nat. Rev. Drug. Discovery.

[56] Feyaerts AF, Mathé L, Luyten W, Van Dijck P (2018). Comparison between the vapor-phase-mediated anti-Candida activity of conventional and organic essential oils. Nat. Volatiles Essent. Oils.

[57] de Groot AC, Schmidt E (2016). Essential oils, part III: Chemical composition. Dermatitis: contact, atopic, occupational, drug..

[58] Leeson PD, Springthorpe B (2007). The influence of drug-like concepts on decision-making in medicinal chemistry. Nat. reviews. Drug. discovery.

[59] Djilani, A. & Dicko, A. In *The therapeutic benefits of essential oils* (Nutrition, well-being and health) (InTech, 2012).

[60] Ghose AK, Viswanadhan VN, Wendoloski JJ (1999). A knowledge-based approach in designing combinatorial or medicinal chemistry libraries for drug discovery. 1. A qualitative and quantitative characterization of known drug databases. J. Comb. Chem..

[61] Muegge I, Heald SL, Brittelli D (2001). Simple selection criteria for drug-like chemical matter. J. medicinal Chem..

[62] Wishart DS (2018). DrugBank 5.0: a major update to the DrugBank database for 2018. Nucleic acids Res..

[63] Cho S-M (2018). A comparative study on the fuel properties of biodiesel from woody essential oil depending on terpene composition. Fuel.

[64] Flashpoints, https://plushfolly.com/Information/Flash-Points (2018).

[65] Lipkin MR, Martin CC (1946). Equation relating density, refractive index, and molecular weight for paraffins and naphthenes. Ind. Eng. Chem. Anal. Ed..

[66] Newman DJ (2016). Developing natural product drugs: Supply problems and how they have been overcome. Pharmacol. Ther..

[67] Simoes, A. J. G. & C. A., H. The Economic Complexity Observatory: An Analytical Tool for Understanding the Dynamics of Economic Development. Workshops at the Twenty-Fifth AAAI Conference on Artificial Intelligence., https://atlas.media.mit.edu/en/visualize/tree_map/hs92/export/show/all/3301/2016/ (2011).

[68] Soltani Howyzeh M, Sadat Noori SA, Shariati JV, Amiripour M (2018). Comparative transcriptome analysis to identify putative genes involved in thymol biosynthesis pathway in medicinal plant Trachyspermum ammi L. Sci. Rep..

[69] Giltrap, A. Total synthesis of natural products with antimicrobial activity. (Springer, 2018).

[70] Morken JP (2018). Practically simple reactions convert hydrocarbons to precious chemicals. Nat..

[71] Inouye S, Takizawa T, Yamaguchi H (2001). Antibacterial activity of essential oils and their major constituents against respiratory tract pathogens by gaseous contact. J. Antimicrobial Chemotherapy.

[72] Azoulay E (2006). Candida colonization of the respiratory tract and subsequent pseudomonas ventilator-associated pneumonia. Chest.

[73] Horváth G, Ács K (2015). Essential oils in the treatment of respiratory tract diseases highlighting their role in bacterial infections and their anti-inflammatory action: a review. Flavour. Fragr. J..

[74] Acs K (2018). Antibacterial activity evaluation of selected essential oils in liquid and vapor phase on respiratory tract pathogens. BMC complementary alternative Med..

[75] Lim PFC, Liu XY, Chan SY (2009). A review on terpenes as skin penetration enhancers in transdermal drug delivery. J. Essent. Oil Res..

[76] Fox LT, Gerber M, Du Plessis J, Hamman JH (2011). Transdermal drug delivery enhancement by compounds of natural origin. Molecules.

[77] Passos, C. S., Arbo, M. D., Rates, S. & Von Poser, G. In *Rev. Bras. Farmacogn.-Braz. J. Pharmacogn*. Vol. 19 140–149 (2009).

[78] Dobetsberger C, Buchbauer G (2011). Actions of essential oils on the central nervous system: An updated review. Flavour. Fragr. J..

[79] Tomi K, Kitao M, Murakami H, Matsumura Y, Hayashi T (2017). Classification of lavender essential oils: sedative effects of Lavandula oils. J. Essent. Oil Res..

[80] Heller SR, McNaught A, Pletnev I, Stein S, Tchekhovskoi D (2015). InChI, the IUPAC International Chemical Identifier. J. Cheminform.

[81] Bajusz, D., Rácz, A. & Héberger, K. In *Comprehensive Medicinal Chemistry III* (eds Chackalamannil, S., Rotella, D. & Ward, S. E.) 329–378 (Elsevier, 2017).

[82] Backman TW, Cao Y, Girke T (2011). ChemMine tools: an online service for analyzing and clustering small molecules. Nucleic acids Res..

[83] Viswanadhan VN, Ghose AK, Revankar GR, Robins RK (1989). Atomic physicochemical parameters for three dimensional structure directed quantitative structure-activity relationships. 4. Additional parameters for hydrophobic and dispersive interactions and their application for an automated superposition of certain naturally occurring nucleoside antibiotics. J. Chem. Inf. Computer Sci..

[84] Klopman G, Li J-Y, Wang S, Dimayuga M (1994). Computer automated log P calculations based on an extended group contribution approach. J. Chem. Inf. Computer Sci..

[85] Bloch D (1995). Computer software review. Review of PHYSPROP database (version 1.0). J. Chem. Inf. Computer Sci..

[86] Tetko IV, Tanchuk VY, Villa AE (2001). Prediction of n-octanol/water partition coefficients from PHYSPROP database using artificial neural networks and E-state indices. J. Chem. Inf. Comput. Sci..

[87] ChemAxon. Drug Discovery Filtering, https://jchem-for-office-docs.chemaxon.com/jchem4office/userguide/drug_discovery_filtering.html (2018).

[88] Ertl P, Rohde B, Selzer P (2000). Fast calculation of molecular polar surface area as a sum of fragment-based contributions and its application to the prediction of drug transport properties. J. medicinal Chem..

[89] Oprea TI, Davis AM, Teague SJ, Leeson PD (2001). Is there a difference between leads and drugs? A historical perspective. J. Chem. Inf. Comput. Sci..

