# Supplementary material for: Striking essential oil: tapping into a largely unexplored source for drug discovery
Source: Sci Rep. 2020 Feb 18;10:2867. doi: 10.1038/s41598-020-59332-5 (PMC7028914; doi:10.1038/s41598-020-59332-5)
Supplement: Supplementary file 1 — Supplementary information. [file 41598_2020_59332_MOESM1_ESM.pdf]

Supplementary information

SI 1: List of EOs (n = 175) used in this study, indicating the different EO subsets used for the robustness check. The EO-subsets are (**A**) EOs of conventional cultivation (n=101), (**B**) EOs of certified-organic cultivation (n=74), and (**C**) all EOs of conventional cultivation, complemented with those EOs of certified-organic cultivation that originated from other plant species, the same plant species but from other plant parts, or the same plant species but a different chemotype (n=141). AP = aerial parts; AP-S = aerial parts - seeds; B = bark; BB = berry branches; F = fruits; FB = flower buds; FL = flowers; FT = flowering tops; H = herbs; L = leaves; LT = leafy twigs; N = needles; O = oleoresin; P = peels; R = roots; RH = rhizome; T = twigs; T + B = twigs + bark; TF = twig flowers; W = wood. # = organic EO; ct = chemotype; ssp = subspecies; cv = cultivar; var = variety.

Plant species	Part of plant	Origin	Lot number	EO in subset(s)
Abies alba	N	Balkans	OF11341	A and C
Abies balsamea#	N	Canada	OF11276	B and C
Abies sibirica	N	Siberia	OF10586	A and C
Achillea millefolium	FT	Balkans	OF10749	A and C
Ammi visnaga#	S	Morocco	OF8569	B and C
Anethum graveolens	F	Ukraine	OF8420	A and C
Angelica archangelica	R	Slovenia	OF1127	A and C
Apium graveolens	F	India	OF10289	A and C
Artemisia dracunculus	FT	France	OF10105	A and C
Artemisia herba alba	FT	Morocco	OF11480	A and C
Cananga odorata extra	FL	Comoros	OF10390	A and C
Cananga odorata totum#	FL	Madagascar	OF9867	B
Canarium luzonicum	O	Philippines	OF9870	A and C
Carum carvi	F	Hungary	000027	A and C
Cedrelopsis grevei#	W	Madagascar	OF9859	B and C
Cedrus atlantica	W	Morocco	OF10992	A and C
Cedrus atlantica#	W	Morocco	OF10799	B
Cedrus deodara	W	India	OF10214	A and C
Chamaemelum nobile	FL	France	OF10863	A and C
Chamaemelum nobile#	FL	Hungary	OF11255	B
Cinnamomum camphora ct cineole#	L	Madagascar	OF11065	B and C
Cinnamomum camphora ct linalool	W	China	OF10369	A and C
Cinnamomum cassia	T	China	OF10584	A and C
Cinnamomum cassia#	T	Vietnam	OF10588	B
Cinnamomum zeylanicum	B	Madagascar	OF10850	A and C
Cinnamomum zeylanicum	L	Madagascar	OF9780	A and C
Cinnamosma fragrans#	L	Madagascar	OF10651	B and C
Cistus ladaniferus ct pinene#	T	Morocco	OF10502	B and C
Citrus aurantifolia	F	Mexico	OF10400	A and C
Citrus aurantium ssp *amara*	L	Paraguay	OF10467	A and C
Citrus aurantium ssp amara	P	Tunisia	OF10404	A and C
Citrus aurantium ssp amara#	FL	Morocco	OF10993	B and C
Citrus aurantium ssp amara#	L	Paraguay	OF11484	B
Citrus aurantium ssp bergamia	P	Italy	OF10862	A and C
Citrus aurantium ssp bergamia#	P	Italy	OF11052	B
Citrus limon	L	Spain	OF3114	A and C
Citrus limon	P	Spain	OF11188	A and C

Plant species	Part of plant	Origin	Lot number	EO in subset(s)
Citrus limon[#]	P	Italy	OF11178	B
Citrus paradisii	P	Argentina	OF9436	A and C
Citrus paradisii[#]	P	South Africa	OF9722	B
Citrus reticulata	L	Egypt	OF9239	A and C
Citrus reticulata	P	Argentina	OF3457	A and C
Citrus reticulata[#]	P	Brazil	OF10644	B
Citrus sinensis	P	Brazil	OF9238	A and C
Citrus sinensis[#]	P	Mexico	OF11321	B
Copaifera officinalis	O	Brazil	OF10996	A and C
Coriandrum sativum	F	Rusia	OF11183	A and C
Corydothymus capitatus	FT	Spain	OF11481	A and C
Crithmum maritimum[#]	FT	France	OF9373	B and C
Cuminum cyminum	F	India	OF9607	A and C
Cupressus sempervirens var stricta	T	France	OF10218	A and C
Cupressus sempervirens var stricta[#]	T	France	OF10846	B
Curcuma longa[#]	R	Madagascar	OF10876	B and C
Cymbopogon citratus	AP	Guatemala	OF10881	A and C
Cymbopogon flexuosus	H	India	OF9994	A and C
Cymbopogon giganteus[#]	L	Madagascar	OF9770	B and C
Cymbopogon martinii var motia	AP	India	OF10011	A and C
Cymbopogon martinii var motia[#]	AP	India	OF9950	B
Cymbopogon nardus	AP	Sri Lanka	OF2106	A and C
Cymbopogon winterianus	AP	Indonesia	OF10851	A and C
Daucus carota var sativus	F	France	OF11585	A and C
Daucus carota var sativus[#]	AP	France	OF4113	B and C
Elettaria cardamomum	F	Guatemala	OF12267	A and C
Eucalyptus citriodora ct citronellal[#]	L	Madagascar	OF10647	B and C
Eucalyptus dives ct piperitone[#]	L	South Africa	OF10872	B and C
Eucalyptus globulus	L	China	OF10646	A and C
Eucalyptus globulus[#]	L	Portugal	OF11274	B
Eucalyptus polybractea ct cryptone[#]	L	France	OF11142	B and C
Eucalyptus radiata ssp radiata	L	Australia	OF10865	A and C
Eucalyptus radiata ssp radiata[#]	L	Madagascar	OF10720	B
Eucalyptus smithii[#]	L	Australia	OF9370	B and C
Eugenia caryophyllus	FB	Madagascar	OF9948	A and C
Eugenia caryophyllus[#]	FB	Madagascar	OF10583	B
Ferula gummosa	O	Iran	OF12273	A and C
Foeniculum vulgare[#]	AP- S	Egypt	OF10796	B and C
Fokiena hodginsii	BB	Vietnam	OF11589	A and C
Gaultheria fragrantissima[#]	L	Nepal	OF11051	B and C
Gaultheria procumbens	L	China	OF11068	A and C
Helichrysum italicum ssp serotinum	FT	Italy	OF9441	A and C
Helichrysum italicum ssp serotinum[#]	FT	Italy	OF10622	B

Plant species	Part of plant	Origin	Lot number	EO in subset(s)
Hyssopus officinalis var officinalis	FT	France	OF11287	A and C
Illicium verum[#]	F	Vietnam	OF10399	B and C
Inula graveolens[#]	FT	France	OF11143	B and C
Juniperus communis ssp communis[#]	T + B	Bulgaria	OF10654	B and C
Juniperus communis var alpina[#]	T	France	OF4120	B and C
Juniperus oxycedrus	T	France	OF9732	A and C
Juniperus virginiana	W	USA	OF9364	A and C
Laurus nobilis	L	Slovenia	OF11039	A and C
Lavandula angustifolia spp angustifolia	FT	France	OF10864	A and C
Lavandula angustifolia spp angustifolia[#]	FT	France	OF10951	B
Lavandula latifolia	FT	Spain	OF10007	A and C
Lavandula latifolia[#]	FT	Spain	OF9693	B
Lavandula stoechas[#]	FT	France	OF11591	B and C
Lavandula x burnatii clone abrialis[#]	FT	France	OF10059	B and C
Lavandula x burnatii clone grosso	FT	France	OF1743	A and C
Lavandula x burnatii clone grosso[#]	FT	France	OF6689	B
Lavandula x burnatii clone reydovan	FT	France	OF9108	A and C
Lavandula x burnatii clone super	FT	France	OF10869	A and C
Lavandula x burnatii clone super[#]	FT	France	OF10745	B
Ledum groenlandicum[#]	TF	Canada	OF9694	B and C
Leptospermum petersonii	L	Australia	OF10212	A and C
Levisticum officinale	R	France	OF9237	A and C
Lippia citriodora	L	Morocco	OF9771	A and C
Litsea citrata	F	China	OF10225	A and C
Litsea citrata[#]	F	Vietnam	OF11261	B
Matricaria recutita	FL	Slovenia	OF10999	A and C
Melaleuca alternifolia	L	Australia	OF11248	A and C
Melaleuca alternifolia[#]	L	Australia	OF10388	B
Melaleuca cajeputi	L	Vietnam	OF10662	A and C
Melaleuca cajeputi[#]	L	Vietnam	OF11405	B
Melaleuca quinquenervia ct cineol	L	Madagascar	OF10731	A and C
Melaleuca quinquenervia ct cineol[#]	L	Madagascar	OF10956	B
Melissa officinalis	AP	Bulgaria	OF10300	A and C
Mentha arvensis	AP	India	OF10883	A and C
Mentha arvensis[#]	AP	India	OF9728	B
Mentha citrata	AP	India	OF9449	A and C
Mentha pulegium	AP	Morocco	OF9582	A and C
Mentha spicata[#]	AP	Morocco	OF12639	B and C
Mentha x piperita	AP	India	OF10867	A and C
Mentha x piperita[#]	AP	India	OF11594	B
Myristica fragrans	F	Indonesia	OF10590	A and C
Myrtus communis ct cineole[#]	L	France	OF9864	B and C
Myrtus communis ct myrtenyl acetate	L	Morocco	OF9391	A and C

Plant species	Part of plant	Origin	Lot number	EO in subset(s)
Myrtus communis ct myrtenyl acetate[#]	L	Morocco	OF10882	B
Nardostachys jatamansi[#]	R	Nepal	OF11047	B and C
Ocimum basilicum ssp basilicum[#]	FT	India	OF10861	B and C
Ocimum sanctum	L	India	OF11260	A and C
Origanum compactum	FT	Morocco	OF10299	A and C
Origanum compactum[#]	FT	Morocco	OF11283	B
Origanum heracleoticum	FT	France	OF11288	A and C
Origanum majorana	FT	Egypt	OF10217	A and C
Origanum majorana ct thujanol	FT	Egypt	OF10871	A and C
Origanum majorana[#]	FT	Egypt	OF9776	B
Pelargonium x asperum	L	Egypt	OF9858	A and C
Pelargonium x asperum cv Bourbon[#]	L	Congo	OF12053	B and C
Petroselinum crispum	L	Serbia	OF11587	A and C
Picea mariana[#]	N	Canada	OF10298	B and C
Pimenta racemosa	F	U.S. Virgin Islands	OF9871	A and C
Pinus pinaster	O	France	OF11277	A and C
Pinus ponderosa	N	Patagonia	OF11050	A and C
Pinus sylvestris	N	Austria	OF11339	A and C
Pinus sylvestris[#]	N	Bulgaria	OF2115	B
Piper nigrum	F	Madagascar	OF9540	A and C
Pistacia lentiscus[#]	LT	Morocco	OF9359	B and C
Pogostemon cablin	FT	Indonesia	OF10211	A and C
Pogostemon cablin[#]	FT	Indonesia	OF9954	B
Ravensara aromatica	B	Madagascar	OF9044	A and C
Ravensara aromatica[#]	L	Madagascar	OF11431	B and C
Rosmarinus officinalis ct camphor[#]	FT	Spain	OF11044	B and C
Rosmarinus officinalis ct cineole	FT	Morocco	OF10655	A and C
Rosmarinus officinalis ct cineole[#]	FT	Morocco	OF10408	B
Rosmarinus officinalis ct verbenone[#]	FT	France	OF10075	B and C
Salvia lavandulifolia	FT	Spain	OF11046	A and C
Salvia officinalis	FT	France	OF10880	A and C
Salvia officinalis[#]	FT	Albania	OF9241	B
Salvia sclarea[#]	FT	France	OF9454	B and C
Santalum austrocaledonicum var austrocaledonicum	W	New Caledonia	OF11042	A and C
Satureja hortensis	FT	France	OF3340	A and C
Satureja montana	FT	France	OF11247	A and C
Solidago canadensis[#]	AP	Canada	OF10723	B and C
Tanacetum annuum	L	Morocco	OF10287	A and C
Thuya occidentalis	T	France	OF9451	A and C
Thymus mastichina	FT	Spain	OF9049	A and C
Thymus satureioides	FT	Morocco	OF10106	A and C
Thymus satureioides[#]	FT	Morocco	OF10589	B
Thymus serpyllum	FT	Balkans	OF10659	A and C

Plant species	Part of plant	Origin	Lot number	EO in subset(s)
Thymus vulgaris ct geraniol	FT	France	OF9453	A and C
Thymus vulgaris ct linalool#	FT	Spain	OF12998	B and C
Thymus vulgaris ct thymol#	FT	Spain	OF10842	B and C
Thymus zygis	FT	Spain	OF9050	A and C
Trachyspermum ammi	F	India	OF9576	A and C
Tsuga canadensis#	N	Canada	OF10884	B and C
Valeriana officinalis	R	Korea	OF9234	A and C
Vetiveria zizanoïdes	R	Indonesia	OF10233	A and C
Zingiber officinalis#	RH	Madagascar	OF11621	B and C

The chemical composition, including metadata, of the EOs used in this study was reported previously; see www.nature.com/articles/s41598-018-22395-6 under the heading electronic supplementary material [54].

SI 2: Numbered (top) list with chemical structure (center) of the u-cmcEOCs (n=627; Box 2) and their respective InChIKeys-14 (bottom). The InChIKeys-14 (for a definition, see **Box 1**) can be complemented with an information-neutral second InChIKey block, i.e. UHFFFAOYSA, to re-establish a full InChIKey

1	2	3	4
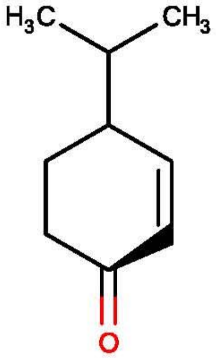	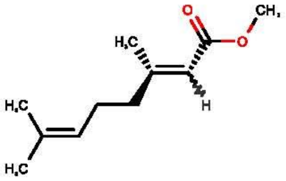	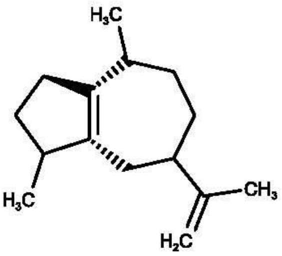	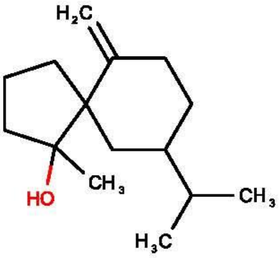
AANMVENRNJYEMK	ACOBBFVLNKYODD	ADIDQIZBYUABQK	AEUBOEQPIBOTGP
**5**	**6**	**7**	**8**
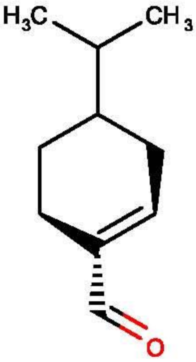	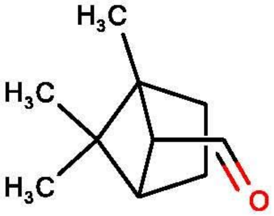	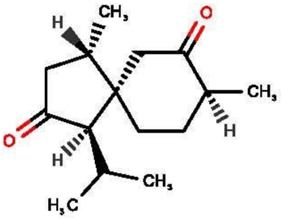	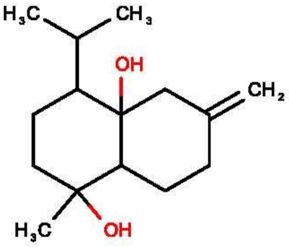
AEVLWICMAHGAMS	AGTSNXAZDKAPHU	AGUISGUERLMHFF	AHNGXHRYFGQWSL
**9**	**10**	**11**	**12**
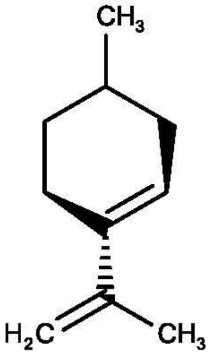	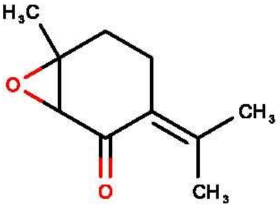	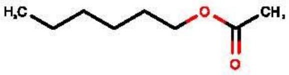	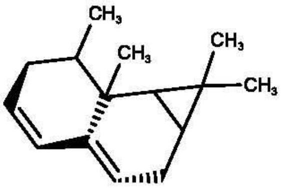
AJSJXSBFZDIRIS	AKASWINDKIEEBO	AOGQPLXWSUTHQB	AOKPBPDKGDLBCJ
**13**	**14**	**15**	**16**
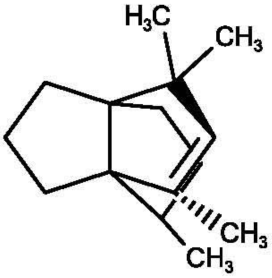	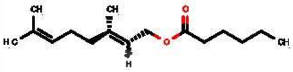	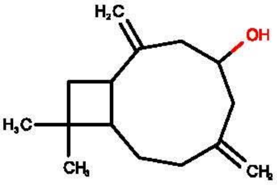	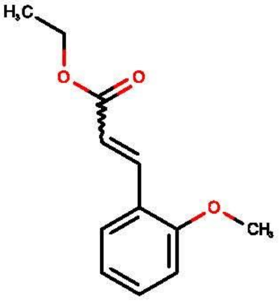
APGXRXFCBZKIAN	ARVSCQUZFFSNKF	ASLIKDGYUZGYOV	ATAFSLBAINHGTN

17	18	19	20
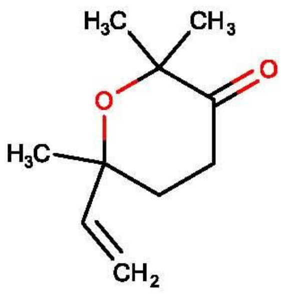	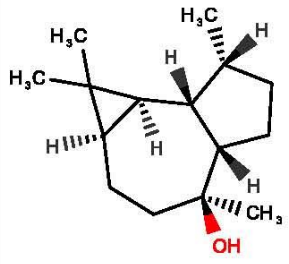	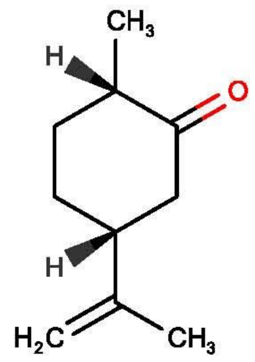	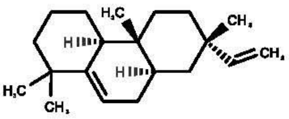
ATQPZCOAQSYTPR	AYXPYQRXGNDJFU	AZOCECCLWFDTAP	BAIWMJSLFJWAQP
**21**	**22**	**23**	**24**
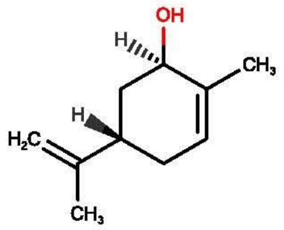	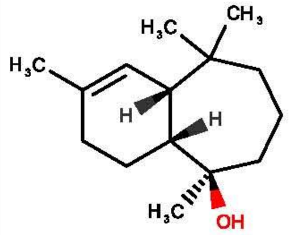	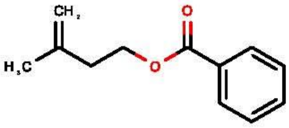	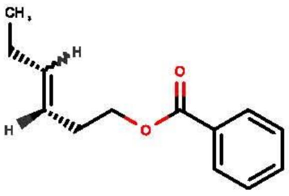
BAVONGHXFVOKBV	BBAMLNIPVMLTSQ	BBTRQSDRTFFNSW	BCOXBEHFBZOJJZ
**25**	**26**	**27**	**28**
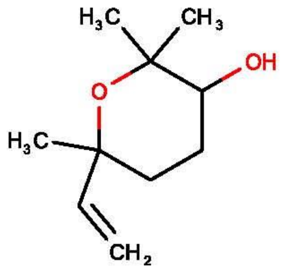	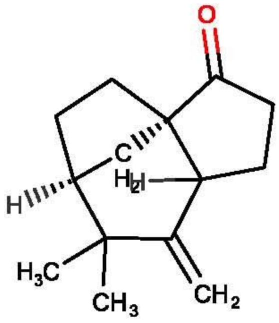	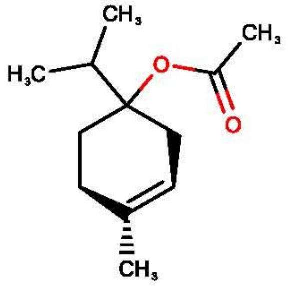	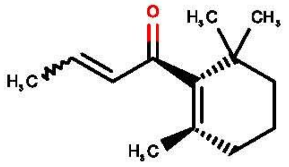
BCTBAGTXFYWYMW	BDHSOIIDXCBNPA	BFCBRSFYYLSTAA	BGTBFNDXYDYBEY
**29**	**30**	**31**	**32**
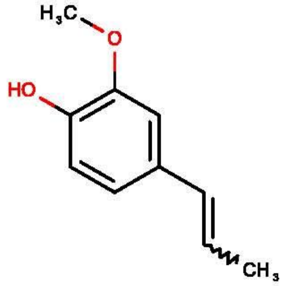	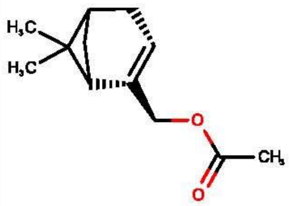	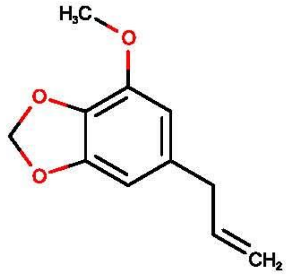	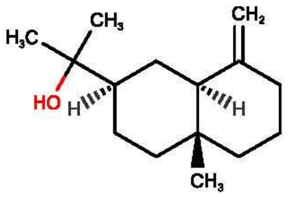
BJIOGJUNALELMI	BKATZVAUANSCKN	BNWJOHGLIBDBOB	BOPIMTNSYWYZOC

33	34	35	36
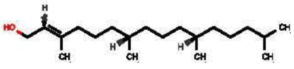	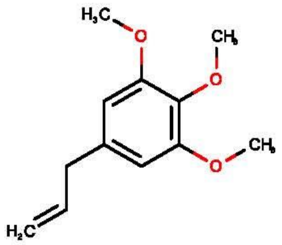	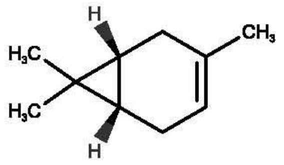	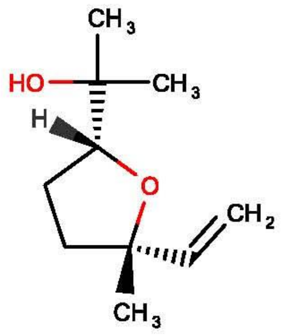
BOTWFXYSPFMFNR	BPLQKQKXWHCZSS	BQOFWKZOCNGFEC	BRHDDEIRQPDPMG
**37**	**38**	**39**	**40**
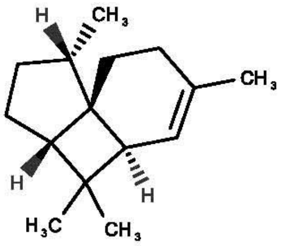	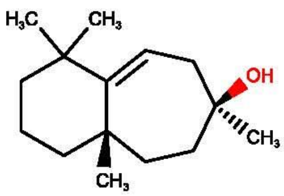	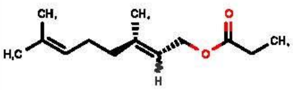	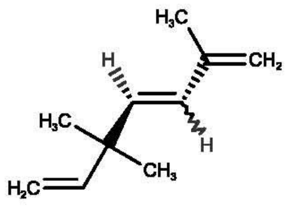
BWAXOYJGXIEEOE	BXGVVQADPFXGHD	BYCHQEILESTMQU	BYLJEQIUXOYKOB
**41**	**42**	**43**	**44**
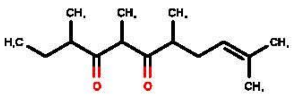	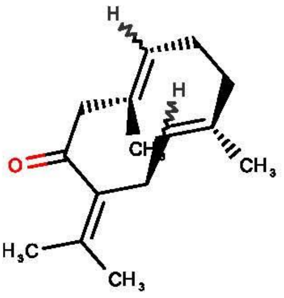	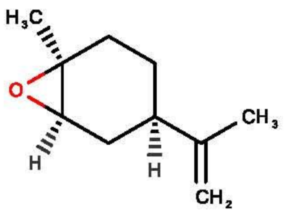	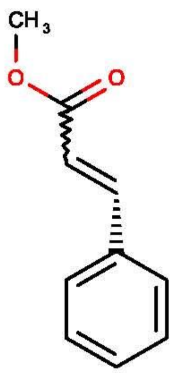
BZBFBQMTXDLSPW	CAULGCQHVOVVRN	CCEFMUBVSUDRLG	CCRCUPLGCSFEDV
**45**	**46**	**47**	**48**
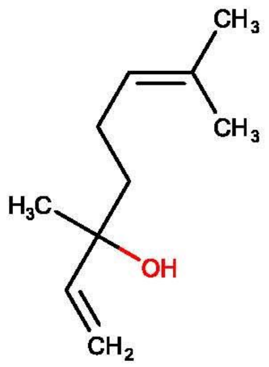	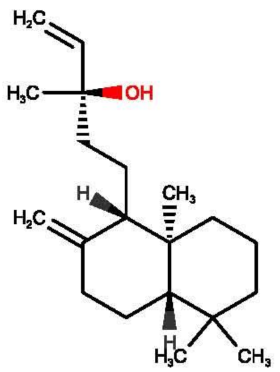	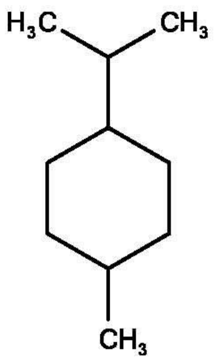	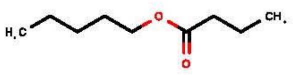
CDOSHBSSFJOMGT	CECREIRZLPLYDM	CFJYNSNXFXLKNS	CFNJLPHOBMVMNS

49	50	51	52
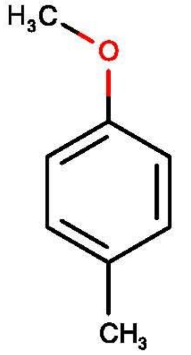	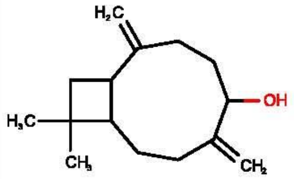	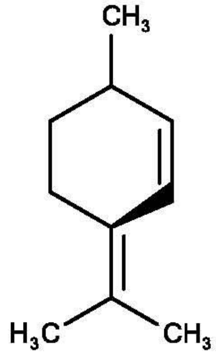	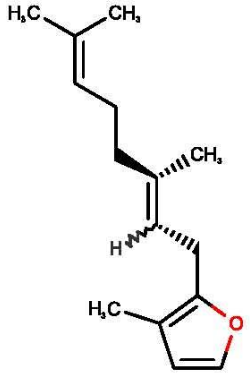
CHLICZRVGGXEOD	CIIYOYPOMGIECX	CIPXOBMYVWRNLL	CKUQXDUZAWSPOV
**53**	**54**	**55**	**56**
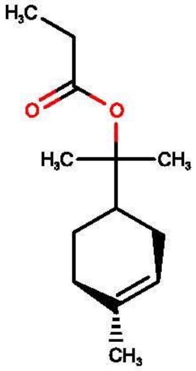	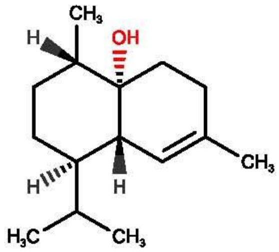	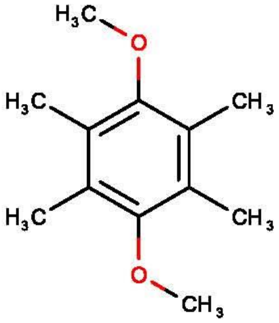	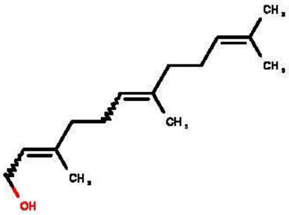
CMKQOKAXUWQAHG	COGPRPSWSKLKTF	CPDNGRVWRPXTGS	CRDAMVZIKSXKFV
**57**	**58**	**59**	**60**
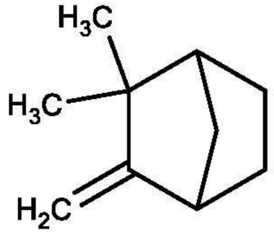	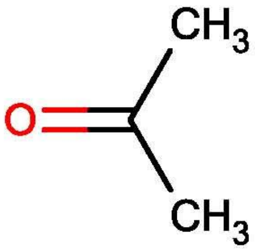	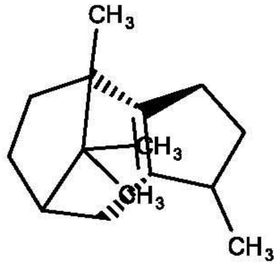	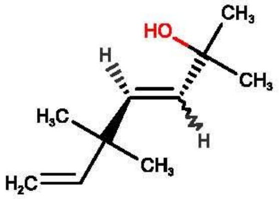
CRPUJAZIXJMDBK	CSCPPACGZOOCGX	CSKINCSXMLCMAR	CSMMFGCGBLZIJE
**61**	**62**	**63**	**64**
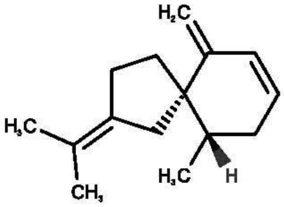	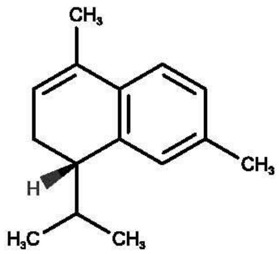	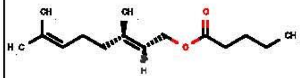	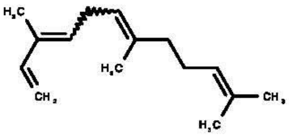
CSRZVTLTICSDRH	CUUMXRBKJIDIAY	CVSWGLSBJFKWMW	CXENHBSYCFFKJS

65

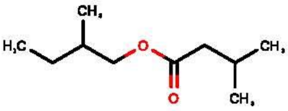

CYGPPWVXOWCHJB

66

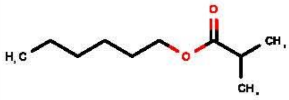

CYHBDKTZDLSRMY

67

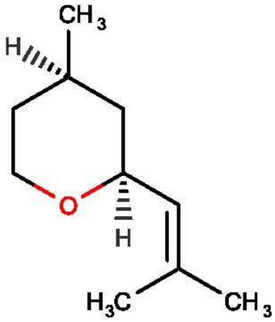

CZCBTSFUTPZVKJ

68

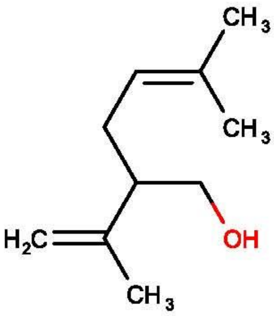

CZVXBFUKBZRMKR

69

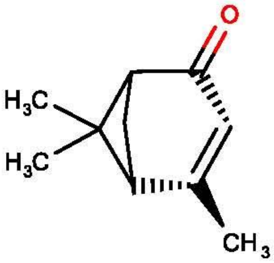

DCSCXTJOXBUFGB

70

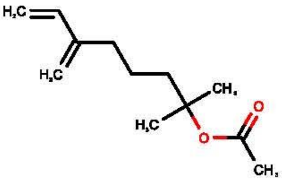

DCXXKSXLKWAZNO

71

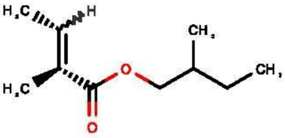

DEJJNOHKWLTTKE

72

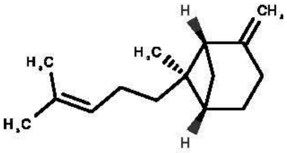

DGZBGCMPRYFWFF

73

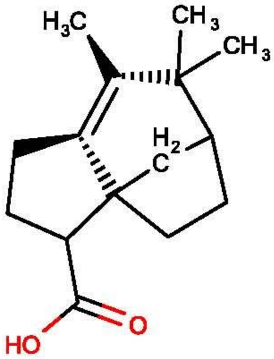

DHPMFKAJSXGYDJ

74

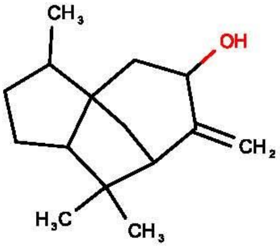

DJYWGTBEZVORGE

75

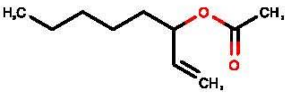

DOJDQRFOTHOBEK

76

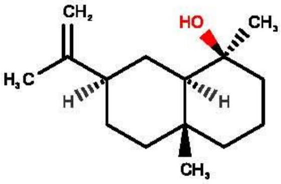

DPQYOKVMVCQHMY

77

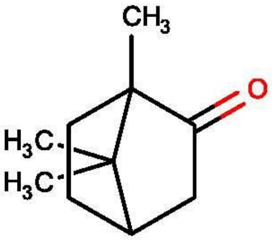

DSSYKIVIOFKYAU

78

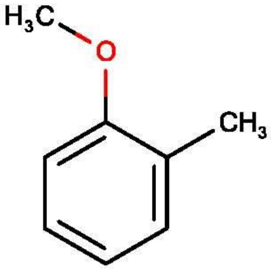

DTFKRVXLBCAIOZ

79

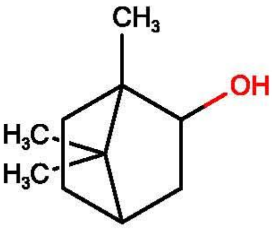

DTGKSKDOIYIVQL

80

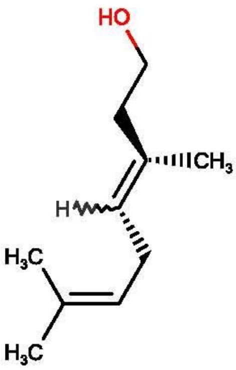

DTHIOPUFUOMHAY

81	82	83	84
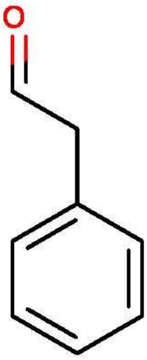	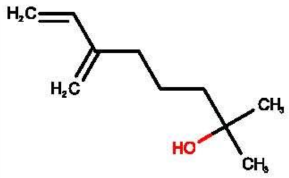	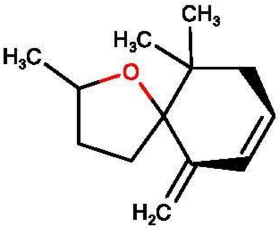	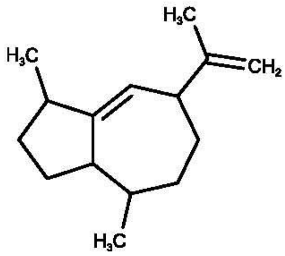
DTUQWGWMVIHBKE	DUNCVNHORHNONW	DUPDJVDPPBFBPL	DUYRYUZIBGFLDD
**85**	**86**	**87**	**88**
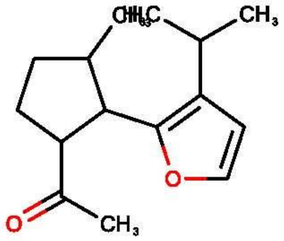	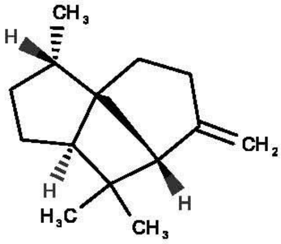	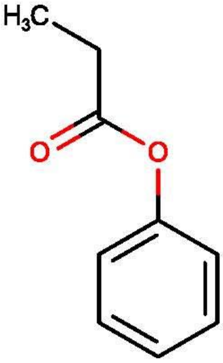	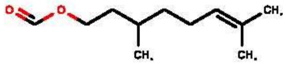
DVIZGXBTTFXQQC	DYLPEFGBWGEFBB	DYUMLJSJISTVPV	DZNVIZQPWLDQHI
**89**	**90**	**91**	**92**
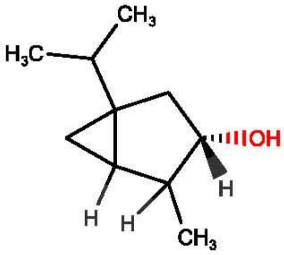	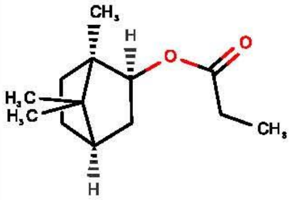	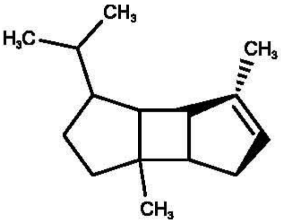	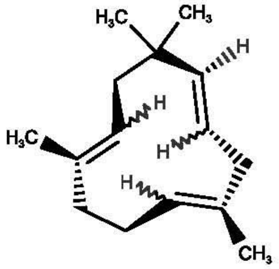
DZVXRFMREAADPP	FAFMZORPAAGQFV	FAIMMSRDTUMTQR	FAMPSKZZVDUYOS
**93**	**94**	**95**	**96**
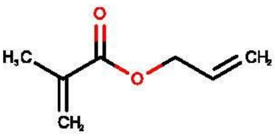	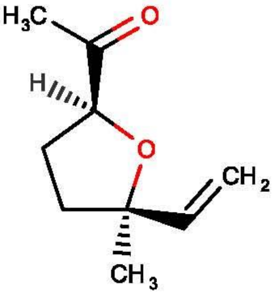	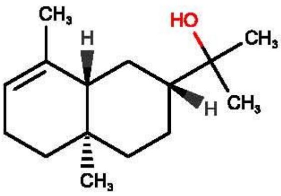	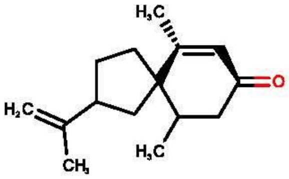
FBCQUCJYYPMKRO	FBFSXARBCWGXJL	FCSRUSQUAVXUKK	FGCUSSRGQNHZRW

97

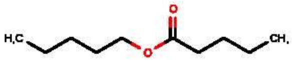

FGPPDYNPZTUNIU

98

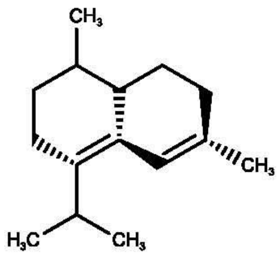

FIAKMTRUEKZMNO

99

FIGVVZUWCLSUEI

100

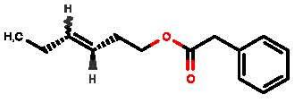

FJKFIIYSBXHBCT

101

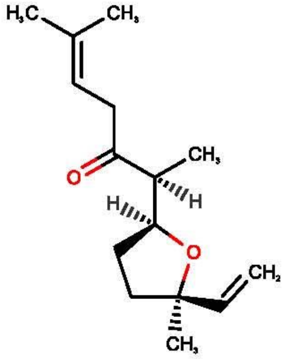

FJKKZNIYYVEYOL

102

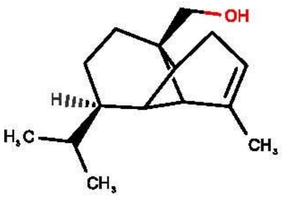

FJQZXWCSOKYVLO

103

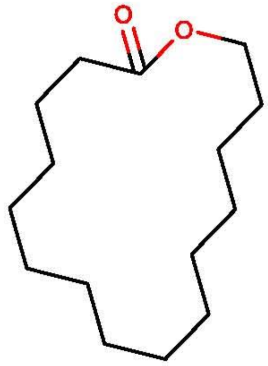

FKUPPRZPSYCDRS

104

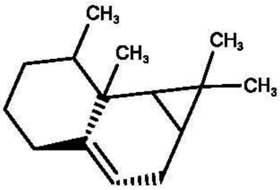

FOBXOZMHEKILEY

105

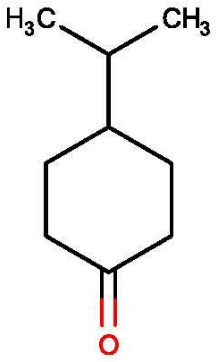

FPKISACHVIIMRA

106

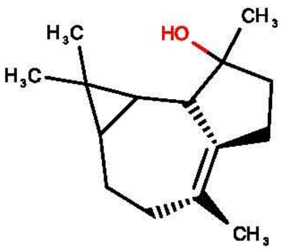

FPRYGNYXOFHMLF

107

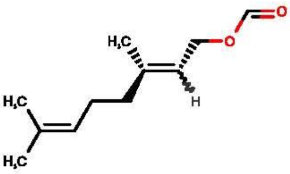

FQMZVFJYMPNUCT

108

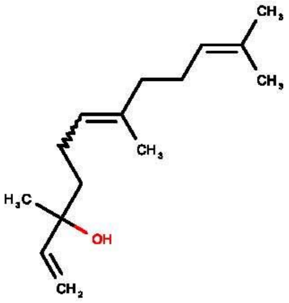

FQTLCLSUCSAZDY

109

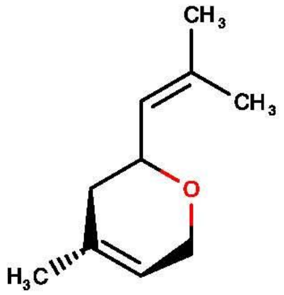

FRISMOQHTLZZRP

110

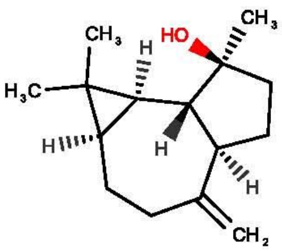

FRMCCTDTYSRUBE

111

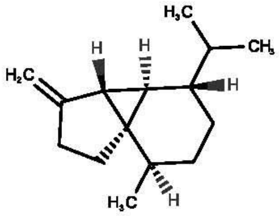

FSRZGYRCMPZNJF

112

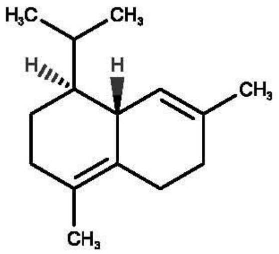

FUCYIEXQVQJBKY

113

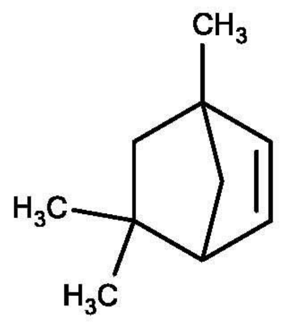

FUIDRYCKEXJNOK

114

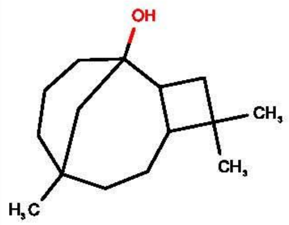

FUQAYSQLAOJBBC

115

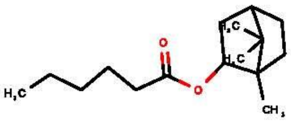

FVTTUTDGUCSLMA

116

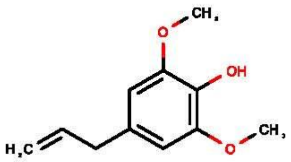

FWMPKHMKIJDEMJ

117

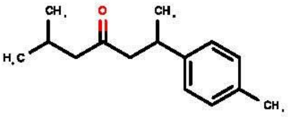

FWSUEHMNQCROMJ

118

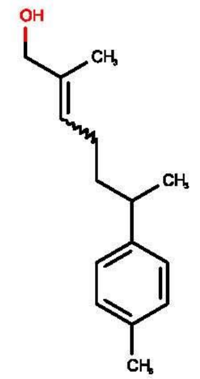

FXCIQPDJVYFUQG

119

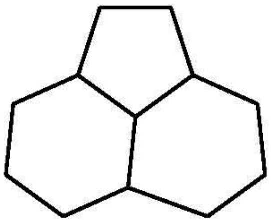

FZDZWLDRELLWNN

120

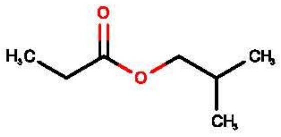

FZXRXKLUIMKDEL

121

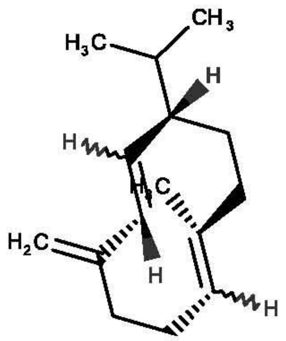

GAIBLDCXCZKKJE

122

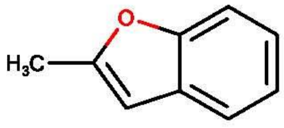

GBGPVUAOTCNZPT

123

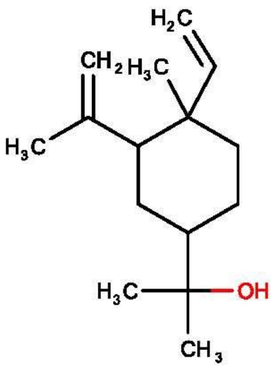

GFJIQNADMLPFOW

124

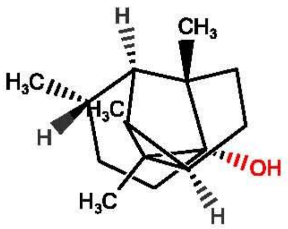

GGHMUJBZYLPWFD

125

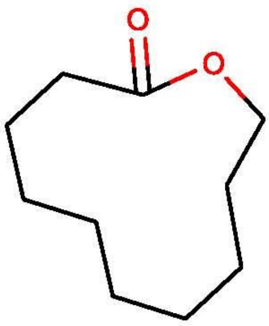

GHZRKQCHJFHJPX

126

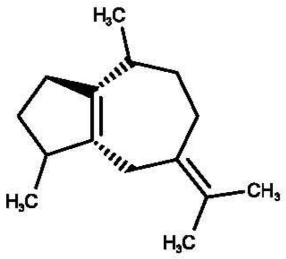

GIBQERSGRNPMEH

127

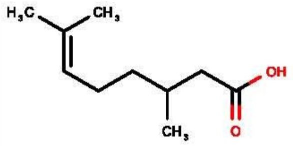

GJWSUKYXUMVMGX

128

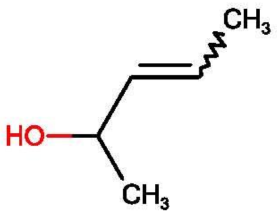

GJYMQFMQRRNLCY

129	130	131	132
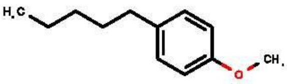	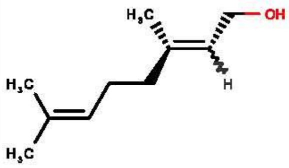	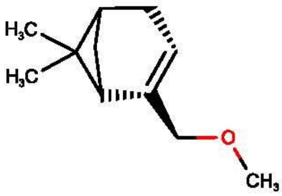	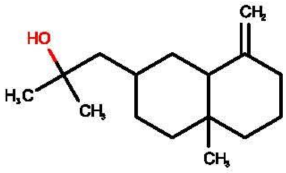
GLWHNBIQKCPVTP	GLZPCOQZEFWAFX	GMYAZJHVKQKHSD	GMZKBWZWDAAWPI
**133**	**134**	**135**	**136**
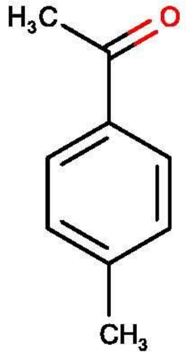	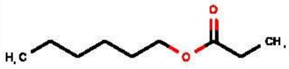	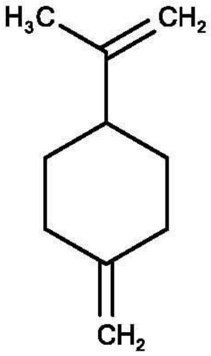	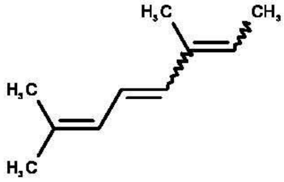
GNKZMNRKLCTJAY	GOKKOFHHJFGZHW	GOQRXDTWKVYHJH	GQVMHMFBVWSSPF
**137**	**138**	**139**	**140**
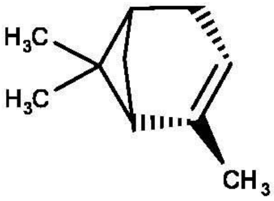	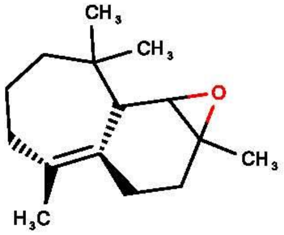	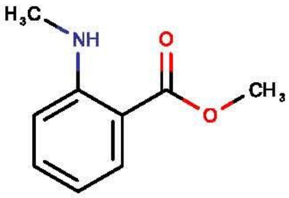	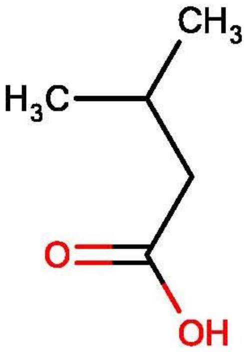
GRWFGVWFFZKLTI	GUMYGCGJGNRHGS	GVOWHGSUZUUUDR	GWYFCOCPABKNJV
**141**	**142**	**143**	**144**
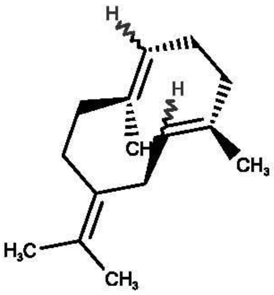	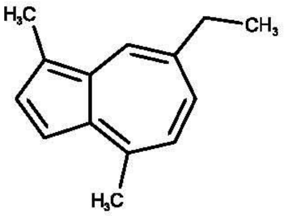	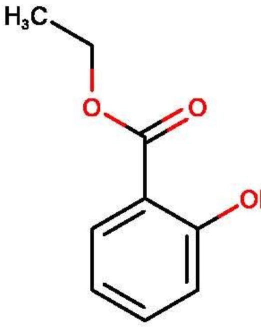	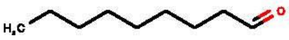
GXEGJTGWYVZSNR	GXGJIOMUZAGVEH	GYCKQBWUSACYIF	GYHFUZHODSMOHU

145	146	147	148
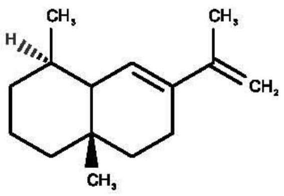	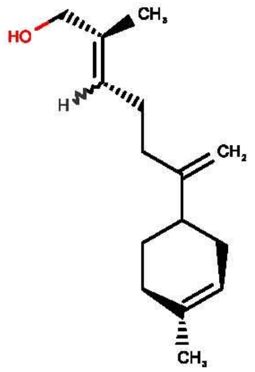	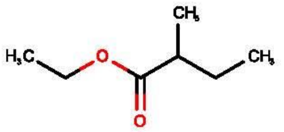	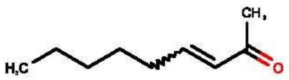
GZTVOICLUQHEMR	HBVOEGGRCJCMLG	HCRBXQFHJMCTLF	HDKLIZDXVUCLHQ
**149**	**150**	**151**	**152**
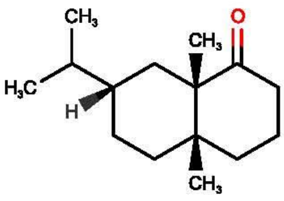	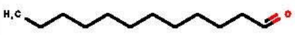	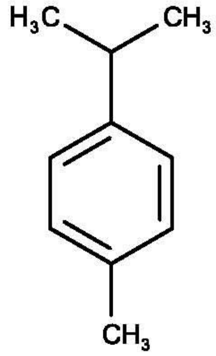	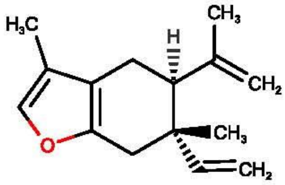
HDVXJTYHXDVWQO	HFJRKMMYBMWEAD	HFPZCAJZSCWRBC	HICAMHOOTMOHPA
**153**	**154**	**155**	**156**
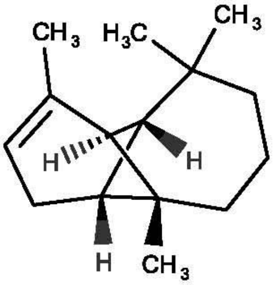	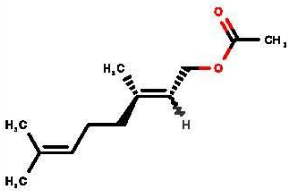	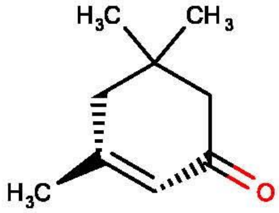	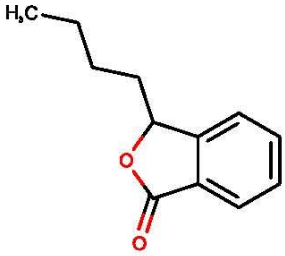
HICYDYJTCDBHMZ	HIGQPQRQIQDZMP	HJOVHMDZYOCNQW	HJXMNVQARNZTEE
**157**	**158**	**159**	**160**
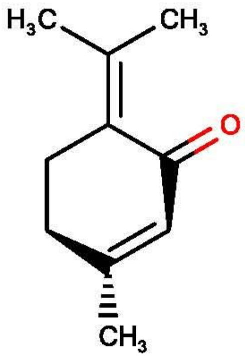	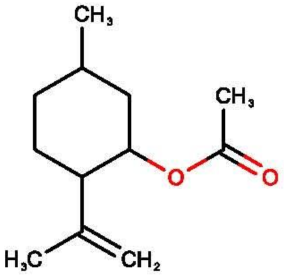	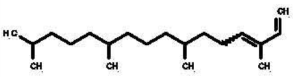	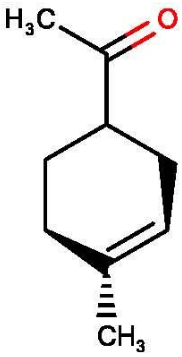
HKZQJZIFODOLFR	HLHIVJRLODSUCI	HNTNJYMWJHGCBD	HOBBEYSRFFJETF

161	162	163	164
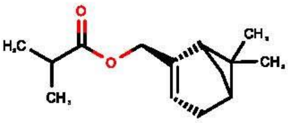	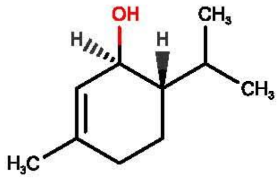	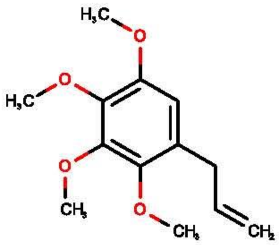	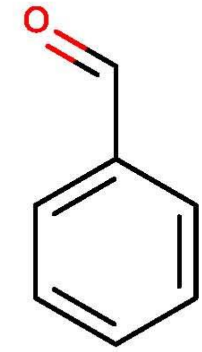
HPAQTXNDJUZELN	HPOHAUWWDDPHRS	HRAXJWRHSUTMCS	HUMNYLRZRPPJDN
**165**	**166**	**167**	**168**
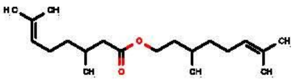	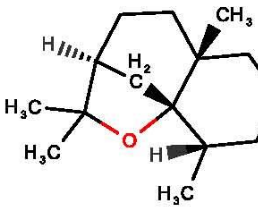	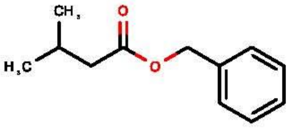	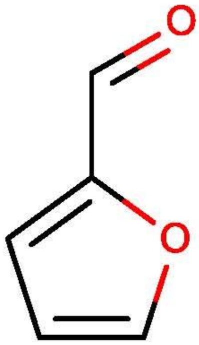
HUZXZYWMBWQTNX	HVAVUZLEYSAYGE	HVJKZICIMIWFCP	HYBBIBNJHNGZAN
**169**	**170**	**171**	**172**
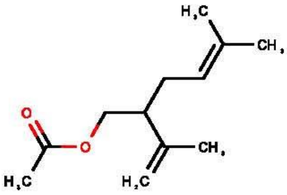	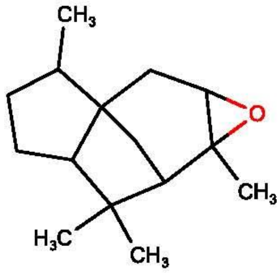	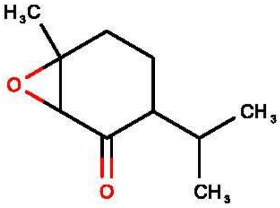	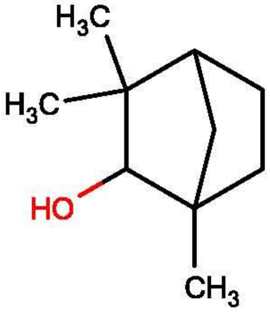
HYNGAVZPWWXQIU	HZRFVTRTTXBHSE	IAFONZHDZMCORS	IAIHUHQCLTYTSF
**173**	**174**	**175**	**176**
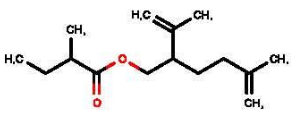	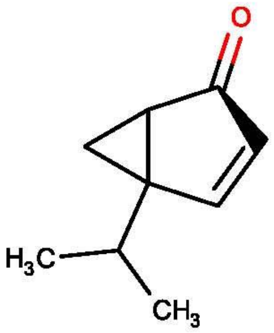	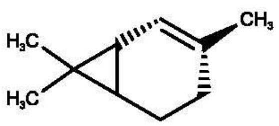	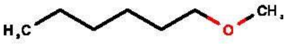
IBKFLDWCDDVSDC	IBMZINAPWMATGM	IBVJWOMJGCHRRW	ICBJCVRQDSQPGI

177

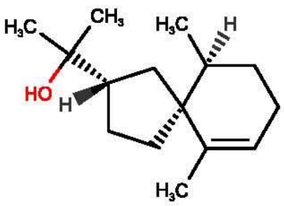

ICWHTQRTTHCUHW

178

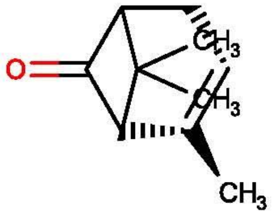

IECBDTGWSQNQID

179

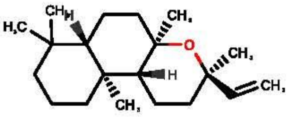

IGGWKHQYMAJOHK

180

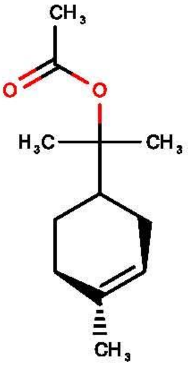

IGODOXYLBBXFDW

181

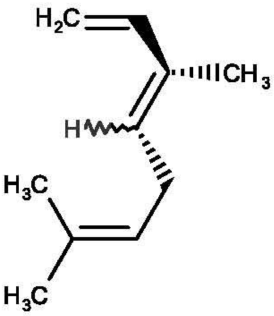

IHPKGUQCSIINRJ

182

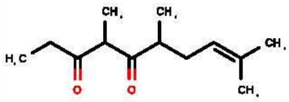

IJGCQHHZHDMDOD

183

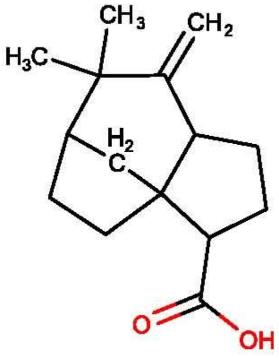

IJGMVUXEZUEDJR

184

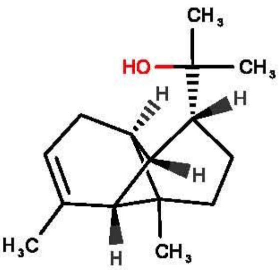

IKIHFZGZEWTHEQ

185

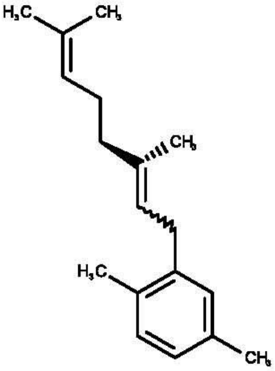

IKVVTAAICQTCAL

186

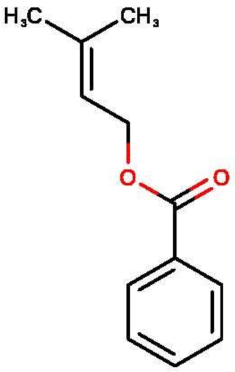

INVWRXWYYVMFQC

187

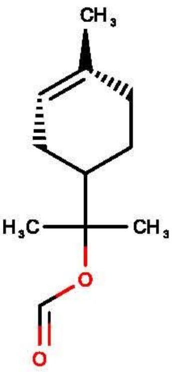

IPYLQIQMGUZFCK

188

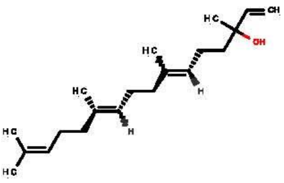

IQDXAJNQKSIPGB

189

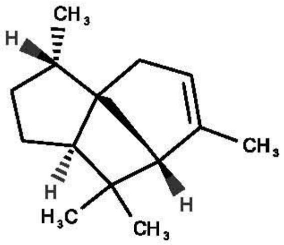

IRAQOCYXUMOFCW

190

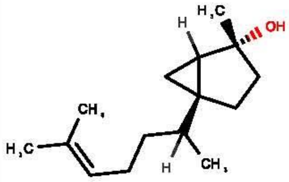

IRDFGGRWKUKANK

191

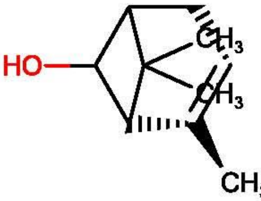

IRZWAJHUWGZMMT

192

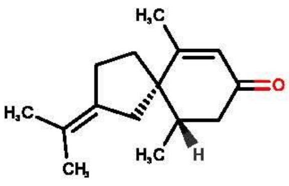

ISLOGSAEQNKPGG

193

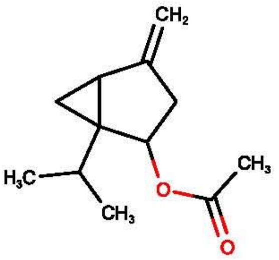

ITQLVLWNPZVVMT

194

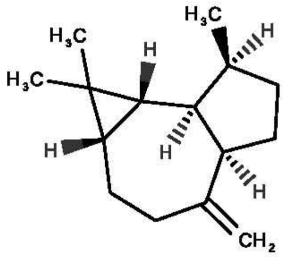

ITYNGVSTWVVPIC

195

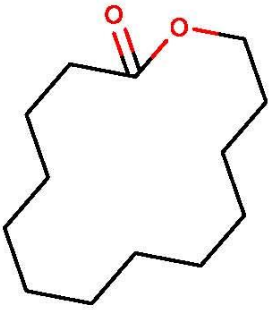

IUDIJIVSWGWJNV

196

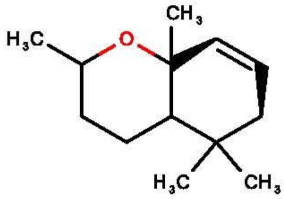

IVTQSEFLDHBCDZ

197

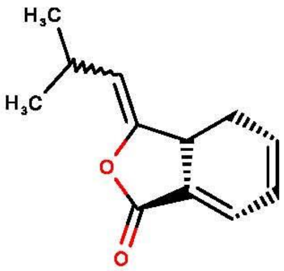

IVZVUDRUEPCTTL

198

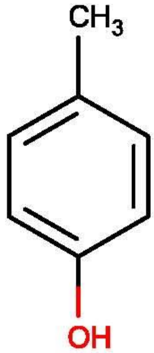

IWDCLRJOBJJRNH

199

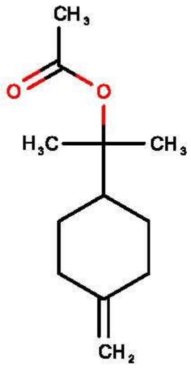

IWKXKWUCSZHJEK

200

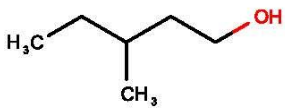

IWTBVKIGCDZRPL

201

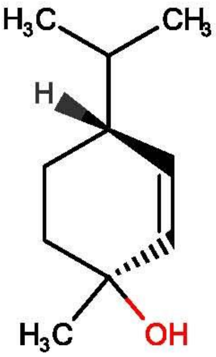

IZXYHAXVIZHGJV

202

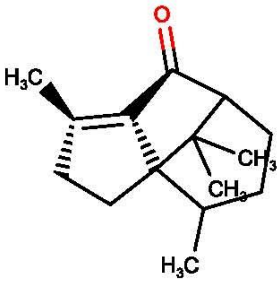

JAWSHISYWRRQQQ

203

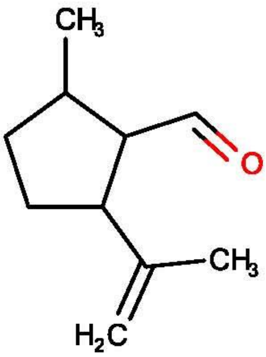

JCDLXWAYWSJVTP

204

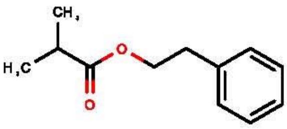

JDQVBGQWADMTAM

205

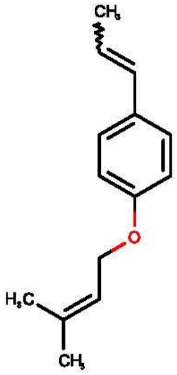

JGELFJUQMIUNOO

206

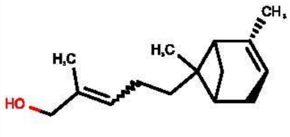

JGINTSAQGRHGMG

207

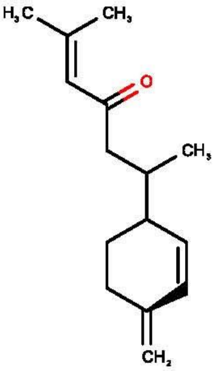

JIJQKFPGBBEJNF

208

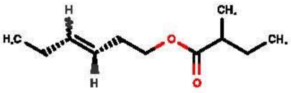

JKKGTSUICJWEKB

209

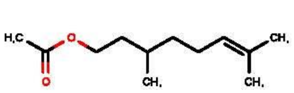

JOZKFWLRHCDGJA

210

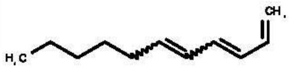

JQQDKNVOSLONRS

211

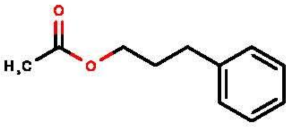

JRJGKUTZNBZHNK

212

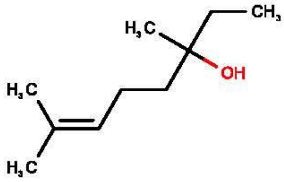

JRTBBCBDKSRRCY

213

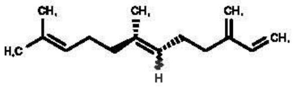

JSNRRGGBADWTMC

214

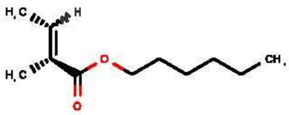

JTCIUOKKVACNCK

215

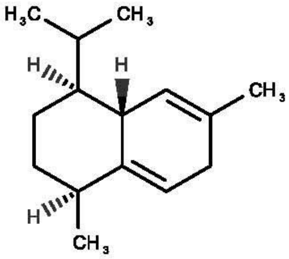

JUQGWBAOQUBVFP

216

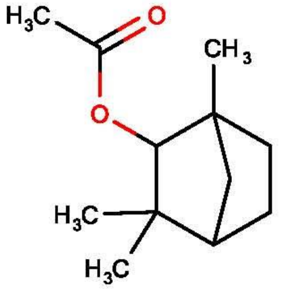

JUWUWIGZUVEFQB

217

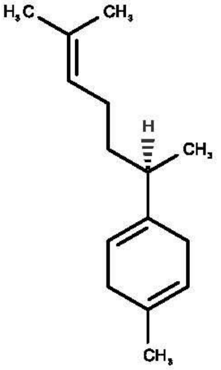

JXZQZARENYGJMK

218

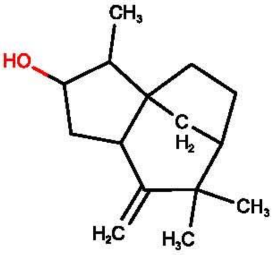

JZLOTPMXLYBVOH

219

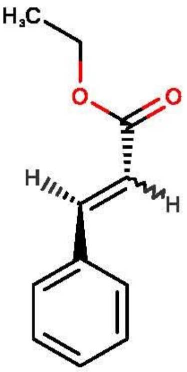

KBEBGUQPQBELIU

220

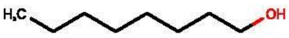

KBPLFHHGFOOTCA

221

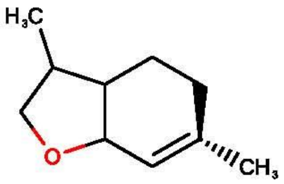

KBPPPUZMFQKLNP

222

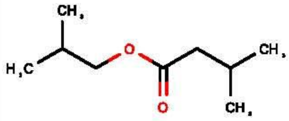

KEBDNKNVCHQIJU

223

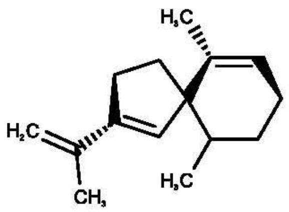

KEVTZKPBXQTBSV

224

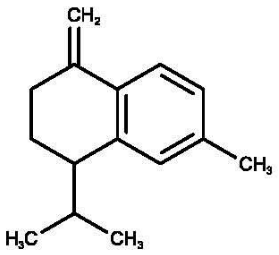

KFYISTOZYAKAPV

225	226	227	228
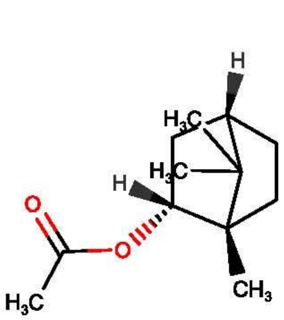	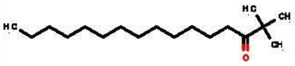	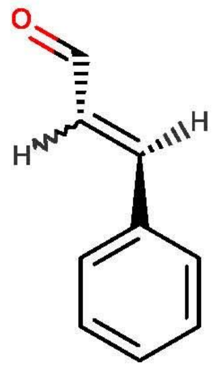	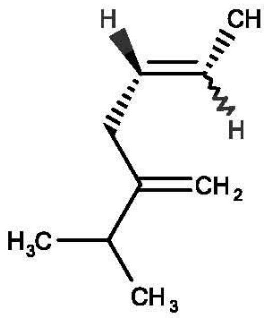
KGEKLUUHTZCSIP	KHQOKXNPFOPAFV	KJPRLNWUNMBNBZ	KKKHJDOOIQCWIL
**229**	**230**	**231**	**232**
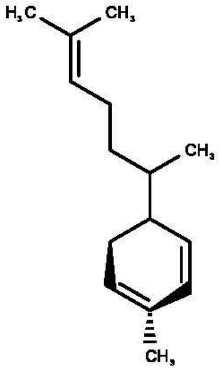	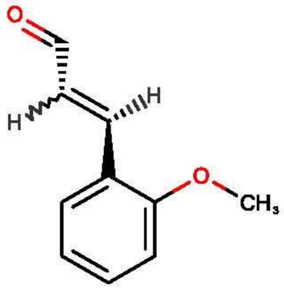	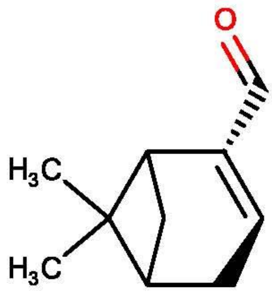	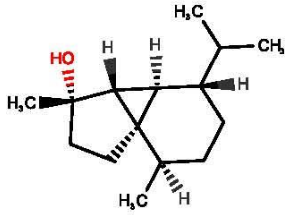
KKOXKGNSUHTUBV	KKVZAVRSVHUSPL	KMRMUZKLFIEVAO	KONGRWVLXLWGDV
**233**	**234**	**235**	**236**
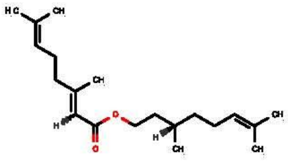	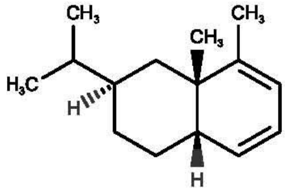	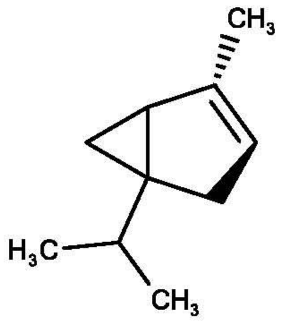	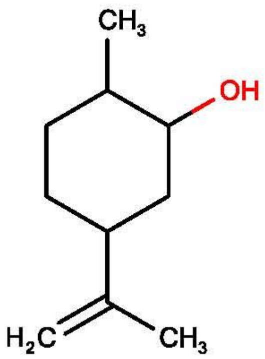
KOTJSBCCZGEHBD	KOTQBSLPXLZGON	KQAZVFVOEIRWHN	KRCZYMFUWVJCLI
**237**	**238**	**239**	**240**
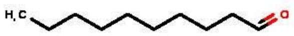	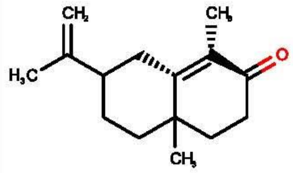	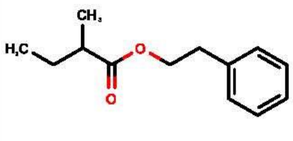	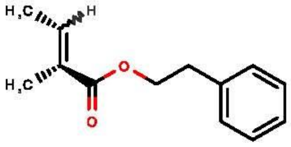
KSMVZQYAVGTKIV	KUFXJZXMWHNCEH	KVKKTLBBYFABAZ	KVMWYGAYARXPOL

241

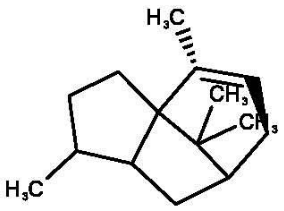

KVQOADNSNSUAJT

242

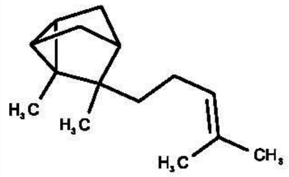

KWFJIXPIFLVMPM

243

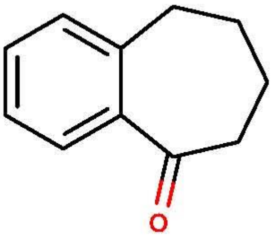

KWHUHTFXMNQHAA

244

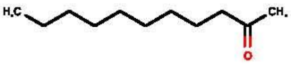

KYWIYKKSMDLRDC

245

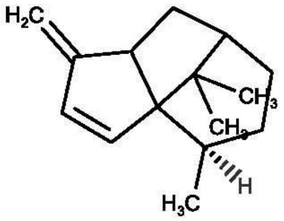

KZABVHBACHSSNR

246

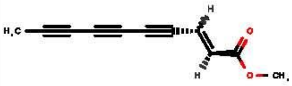

LBAVIXQTLKRIGP

247

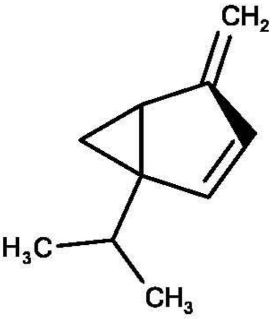

LBVRQJWOZIMWNY

248

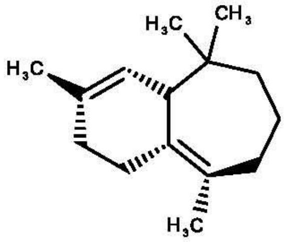

LCOSCMLXPAQCLQ

249

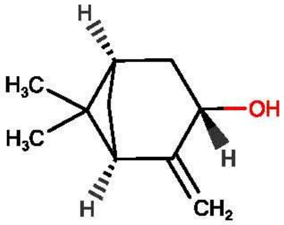

LCYXQUJDODZYIJ

250

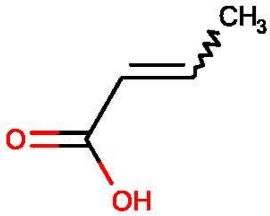

LDHQCZJRKDOVOX

251

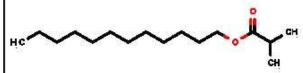

LDPLLPONRGVDGK

252

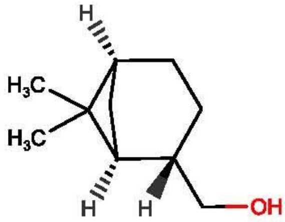

LDWAIHWGMRVEFR

253

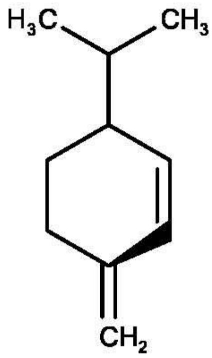

LFJQCDVYDGGFCH

254

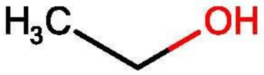

LFQSCWFLJHTTHZ

255

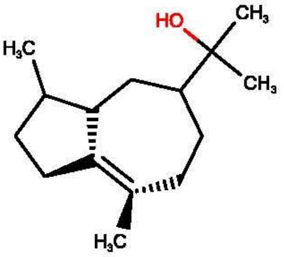

LGOFSGDSFQNIAT

256

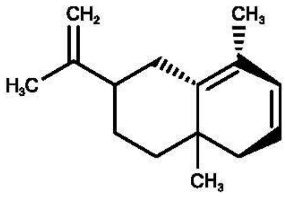

LGRISOGTKBAJPA

257	258	259	260
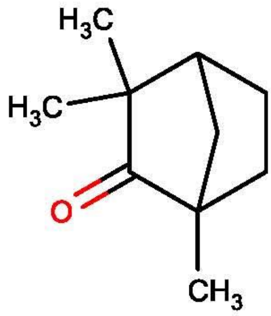	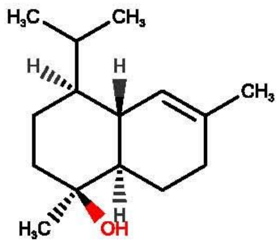	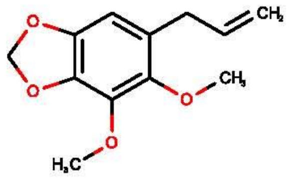	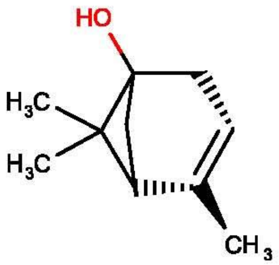
LHXDLQBQYFFVNW	LHYHMMRYTDARSZ	LIKYNOPXHGPMIH	LKGUYHHEOJFHPV
**261**	**262**	**263**	**264**
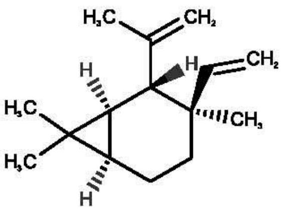	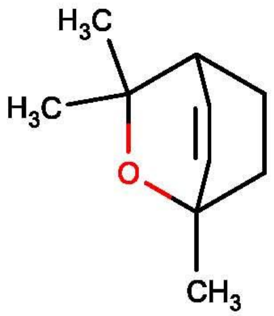	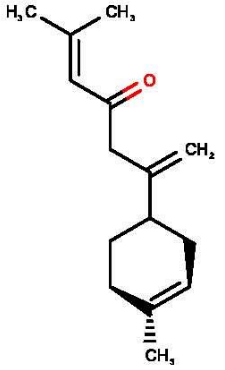	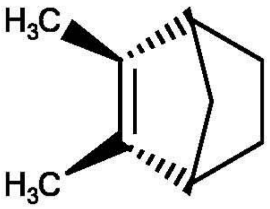
LKQMMFFQYMYQOJ	LOOYOTLEOHYYOV	LRDZCBICVMXPBB	LSIXBBPOJBJQHN
**265**	**266**	**267**	**268**
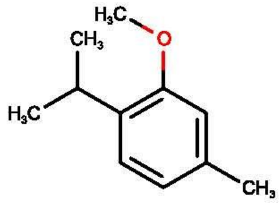	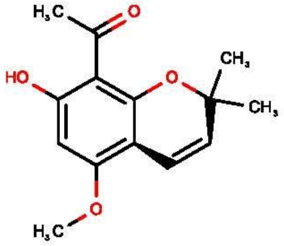	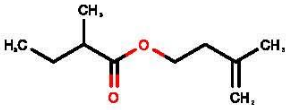	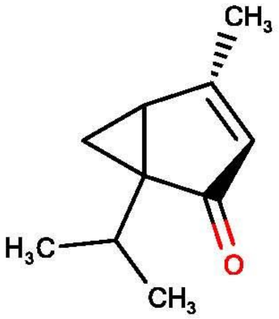
LSQXNMXDFRRDSJ	LTDSNAYFLFUPPT	LTOLHPXUSLBUHD	LTTVJAQLCIHAFV
**269**	**270**	**271**	**272**
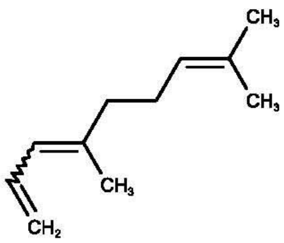	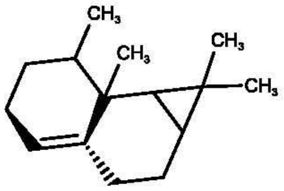	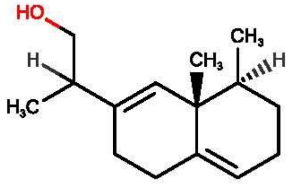	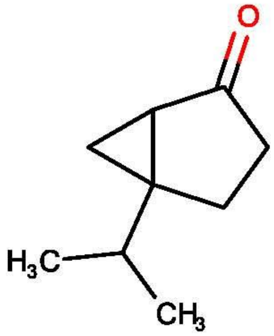
LUKZREJJLWEWQM	MBIPADCEHSKJDQ	MCDRFHDZJOGPFL	MDDYCNAAAZKNAJ

273	274	275	276
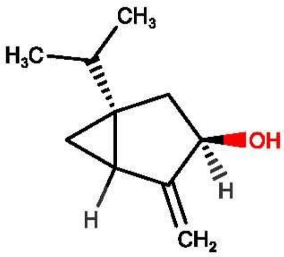	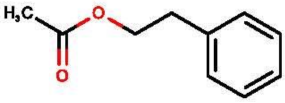	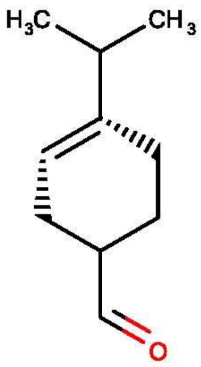	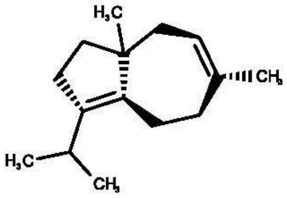
MDFQXBNVOAKNAY	MDHYEMXUFSJLGV	MGJVBPPEWVKZTN	MGMBZNCFUFRSSP
**277**	**278**	**279**	**280**
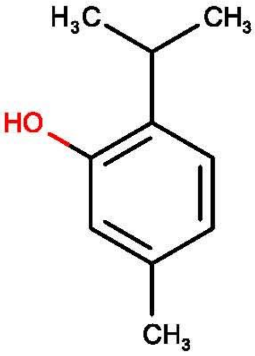	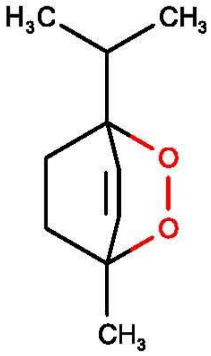	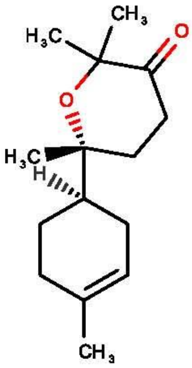	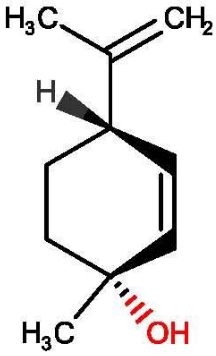
MGSRCZKZVOBKFT	MGYMHQJELJYRQS	MJWZYBQLHJQQJJ	MKPMHJQMNACGDI
**281**	**282**	**283**	**284**
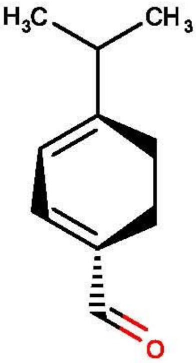	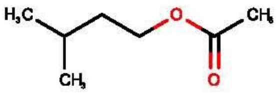	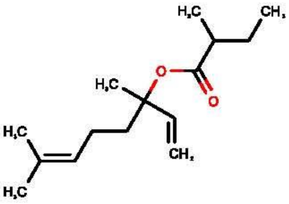	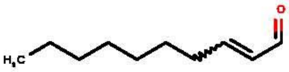
MKVBITWQDIIUMF	MLFHJEHSLIIPHL	MLJNGBGMEMFTPV	MMFCJPPRCYDLLZ
**285**	**286**	**287**	**288**
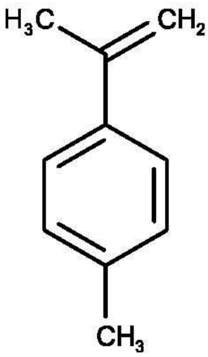	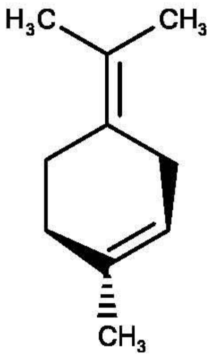	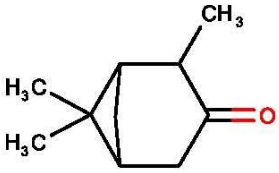	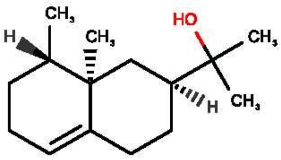
MMSLOZQEMPDGPI	MOYAFQVGZZPNRA	MQPHVIPKLRXGDJ	MQWIFDHBNGIVPO

289	290	291	292
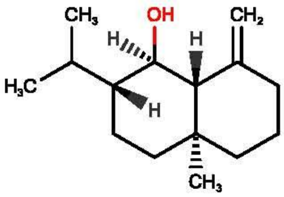	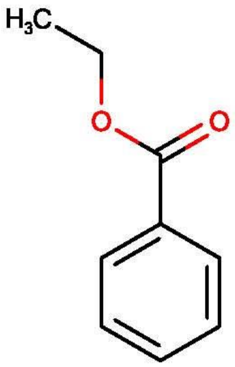	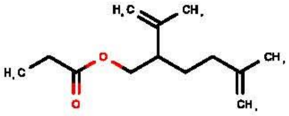	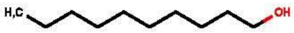
MSJJKJCIFIGTJY	MTZQAGJQAFMTAQ	MUZPKOFBDLINTJ	MWKFXSUHUHTGQN
**293**	**294**	**295**	**296**
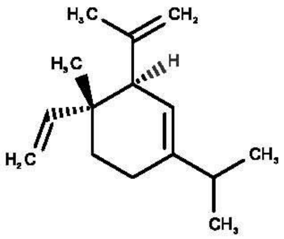	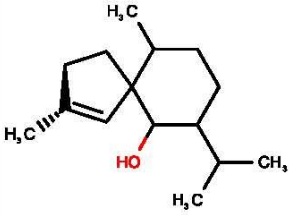	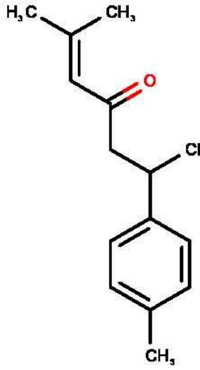	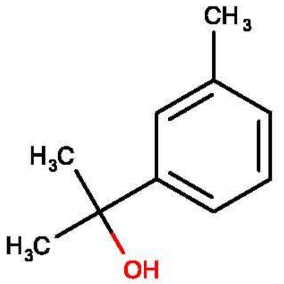
MXDMETWAEGIFOE	MYLXGCVCCZCOHU	NAAJVHHFAXWBOK	NARIBLVZTLPQJB
**297**	**298**	**299**	**300**
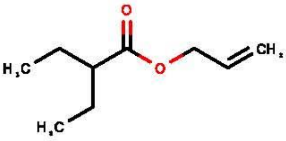	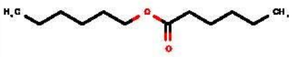	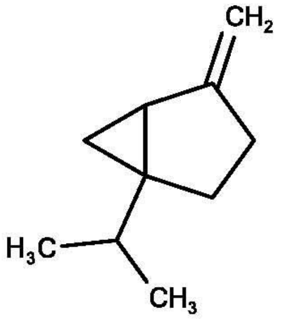	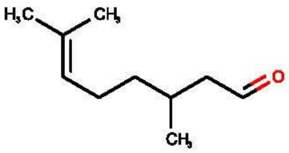
NBKXNUWCFMZFMM	NCDCLPBOMHPFCV	NDVASEGYNIMXJL	NEHNMFOYXAPHSD
**301**	**302**	**303**	**304**
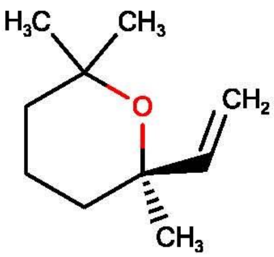	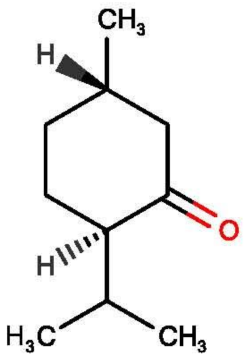	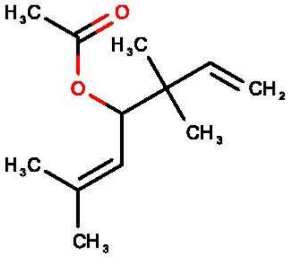	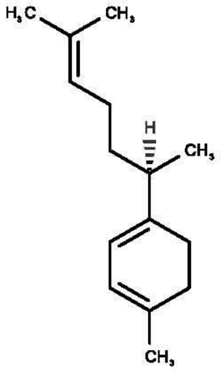
NETOHYFTCONTDT	NFLGAXVYCFJBMK	NGIKFWJEQGLTBM	NGIVKZGKEPRIGG

305	306	307	308
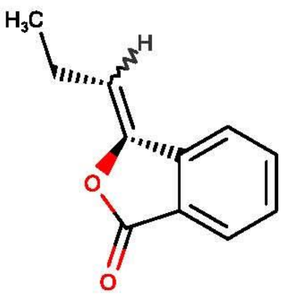	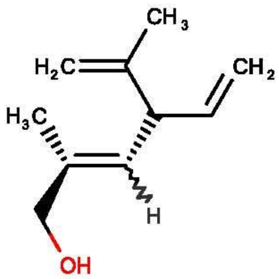	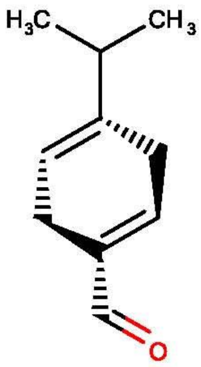	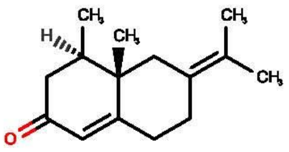
NGSZDVVHIGAMOJ	NHJXCMQPMLBAMK	NIBUJHLMVJZODW	NIIPDXITZPFFTE

309	310	311	312
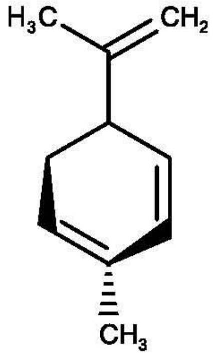	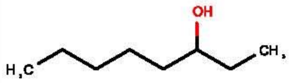	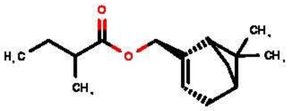	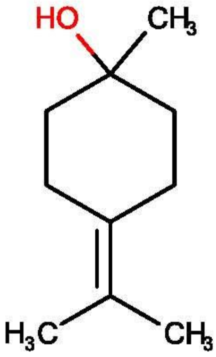
NJLNIOKPXKKALD	NMRPBPVERJPACX	NMVOOLZURJHGKQ	NNRLDGQZIVUQTE

313	314	315	316
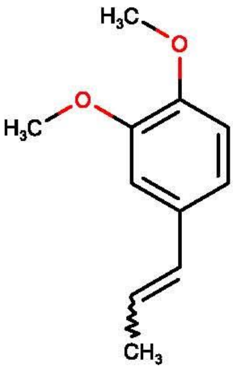	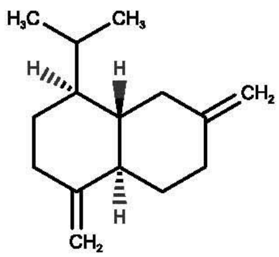	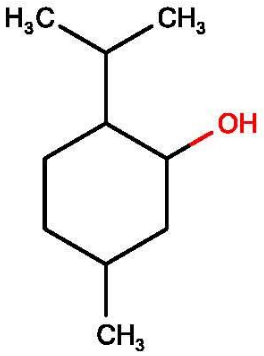	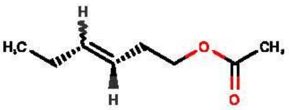
NNWHUJCUHAELCL	NOLWRMQDWRAODO	NOOLISFMXDJSKH	NPFVOOAXDOBMCE

317	318	319	320
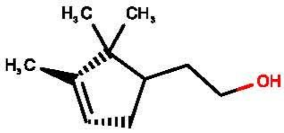	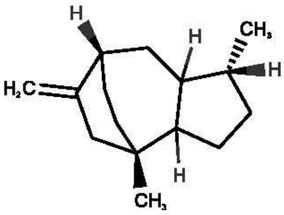	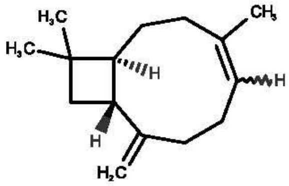	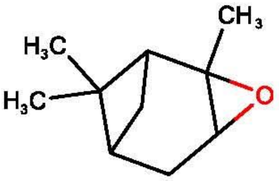
NPGPPCSBEMHHCR	NPHFULIVCUBDDN	NPNUFJAVOOONJE	NQFUSWIGRKFAHK

321

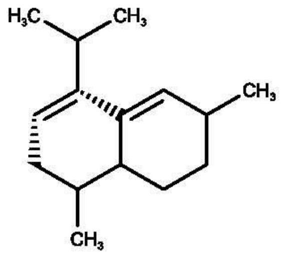

NTWBICNIZKFDJV

322

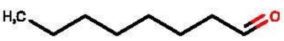

NUJGJRNETVAIRJ

323

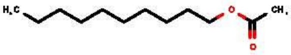

NUPSHWCALHZGOV

324

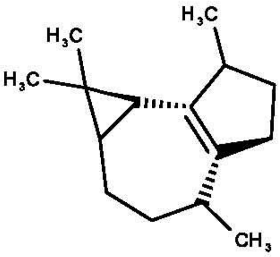

NUQDPKOFUKFKFD

325

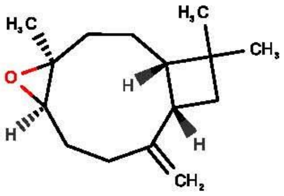

NVEQFIOZRFFVFW

326

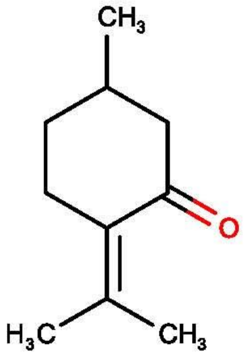

NZGWDASTMWDZIW

327

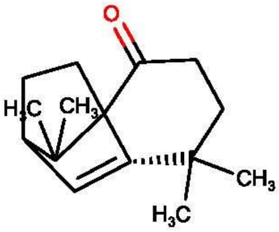

OAUCHXPHYIWYEW

328

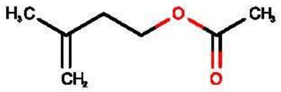

OCUAPVNNQFAQSM

329

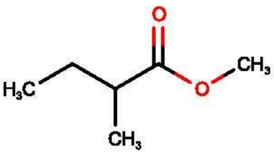

OCWLYWIFNDCWRZ

330

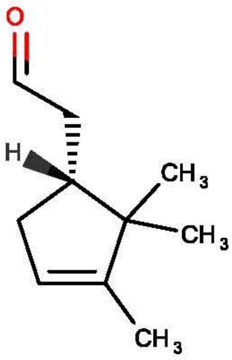

OGCGGWYLHSJRFY

331

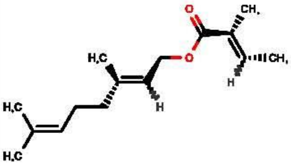

OGHBUHJLMHQMHS

332

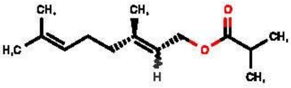

OGJYXQFXLSCKTP

333

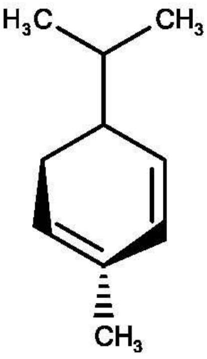

OGLDWXZKYODSOB

334

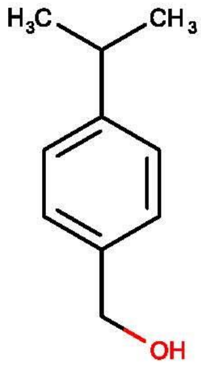

OIGWAXDAPKFNCQ

335

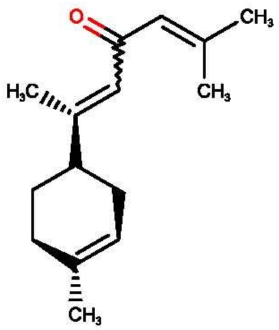

OJEFBZMKKJTKKK

336

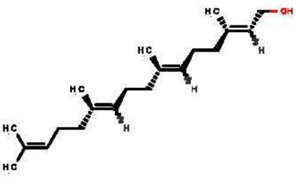

OJISWRZIEWCUBN

337

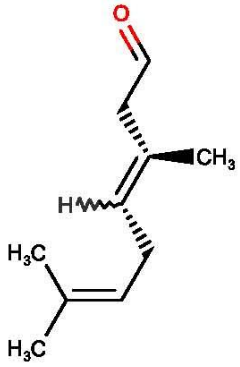

OJLMARCQPSGYNE

338

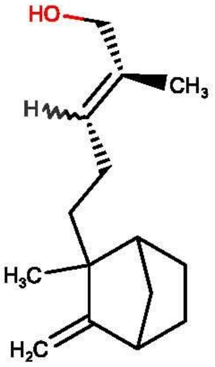

OJYKYCDSGQGTRJ

339

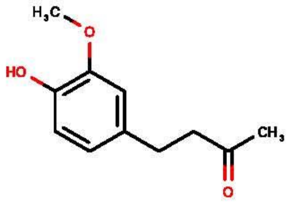

OJYLAHXKWMRDGS

340

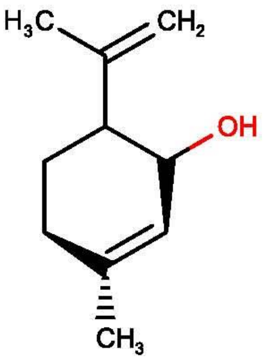

OLAKPNFIICOONC

341

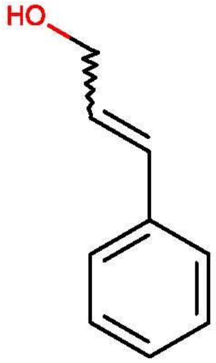

OOCCDEMITAIZTP

342

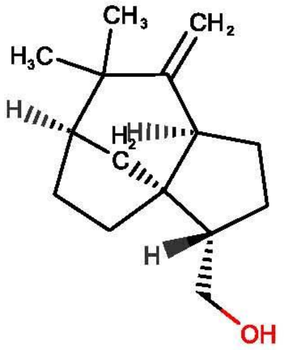

OOYRHNIVDZZGQV

343

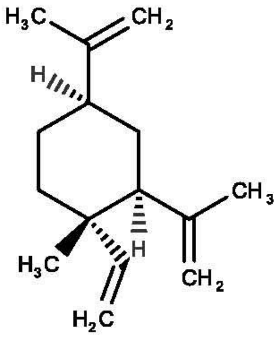

OPFTUNCRGUEPRZ

344

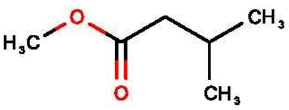

OQAGVSWESNCJJT

345

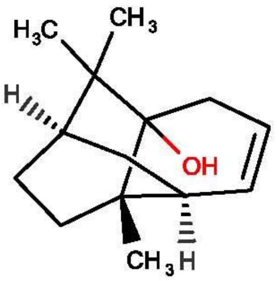

OSQSDJNIURJARY

346

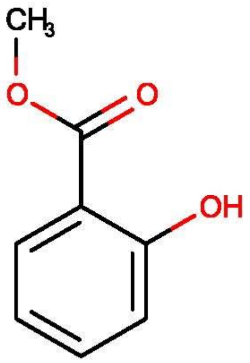

OSWPMRLSEDHDFF

347

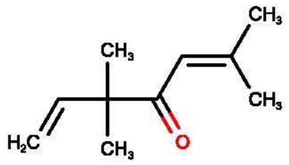

OTYVBQZXUNBRTK

348

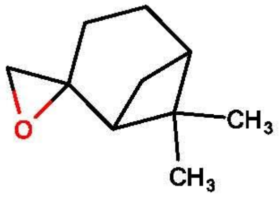

OUXAABAEPHHZPC

349

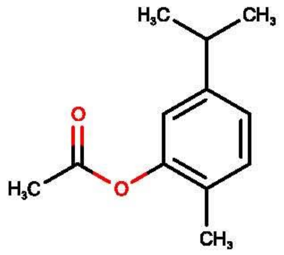

OXZSUQJHKQOGOK

350

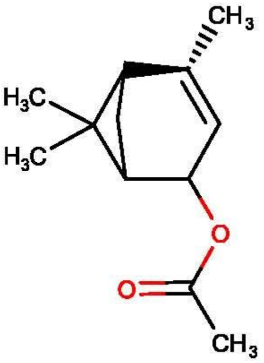

OZBFUQLOVFXDNK

351

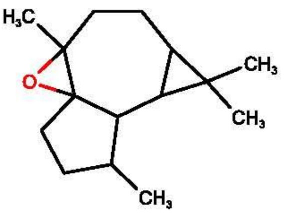

OZNHATCGPKOFBH

352

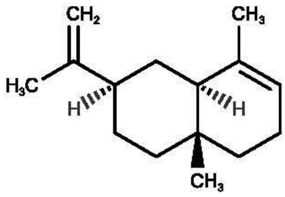

OZQAPQSEYFAMCY

353

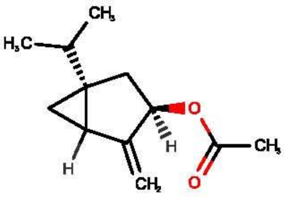

PBWRFXQNNGSAQG

354

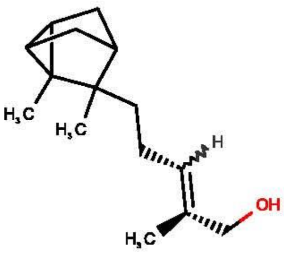

PDEQKAVEYSOLJX

355

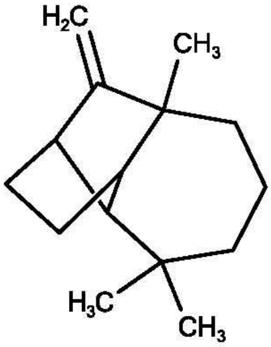

PDSNLYSELAIEBU

356

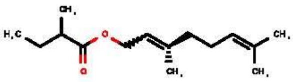

PEQMAZJTEUEQJP

357

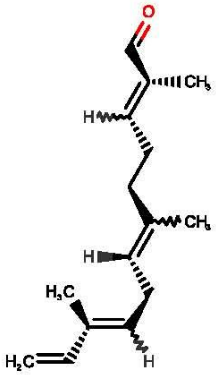

PFSTYGCNVAVZBK

358

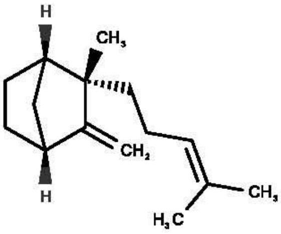

PGBNIHXXFQBCPU

359

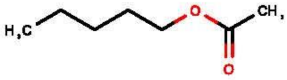

PGMYKACGEOXYJE

360

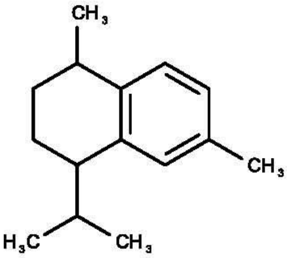

PGTJIOWQJWHTJJ

361

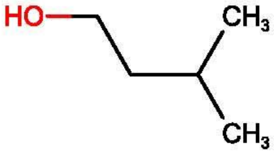

PHTQWCKDNZKARW

362

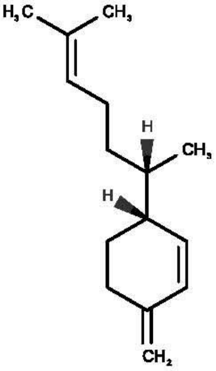

PHWISBHSBNDZDX

363

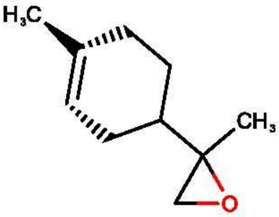

PJGRMBOWSWHGDV

364

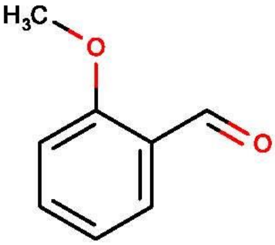

PKZJLOCLABXVMC

365

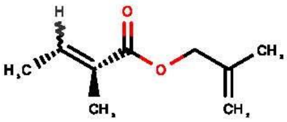

PNRCWIZNCBKLMH

366

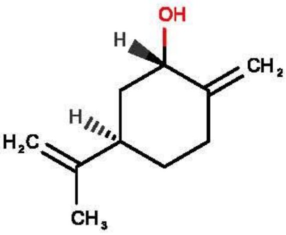

PNVTXOFNJFHXOK

367

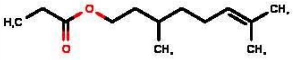

POPNTVRHTZDEBW

368

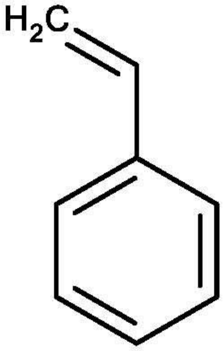

PPBRXRYQALVLMV

369	370	371	372
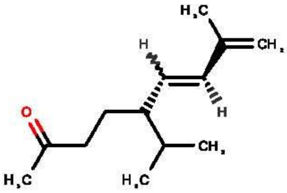	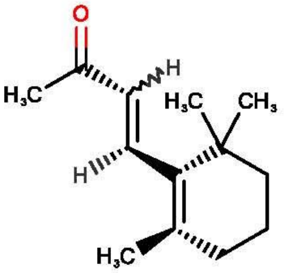	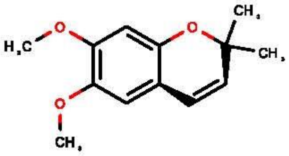	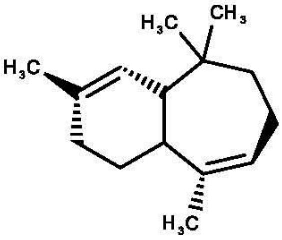
PQDRXUSSKFWCFA	PSQYTAPXSHCGMF	PTIDGSWTMLSGAH	PUWNTRHCKNHSAT
**373**	**374**	**375**	**376**
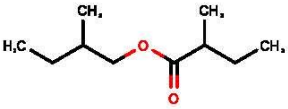	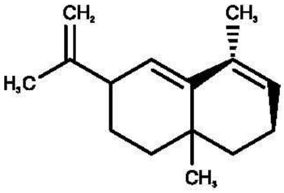	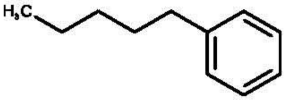	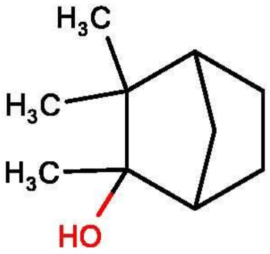
PVYFCGRBIREQLL	PWAPUSDWDZRECY	PWATWSYOIIXYMA	PXRCIOIWVGAZEP
**377**	**378**	**379**	**380**
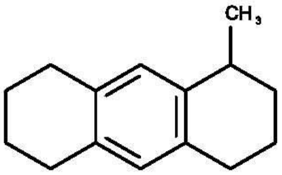	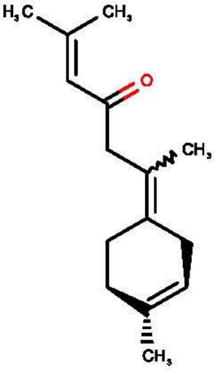	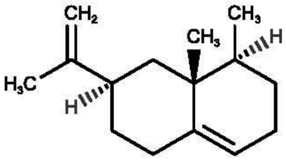	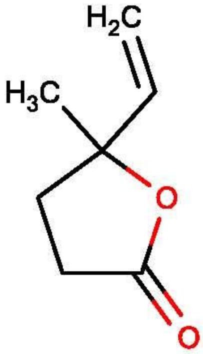
PZMUIEZEAWSHNE	QEAHSEZXAQIWSW	QEBNYNLSCGVZOH	QESPSAHXYXIGBG
**381**	**382**	**383**	**384**
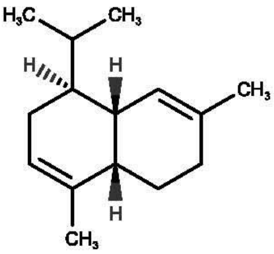	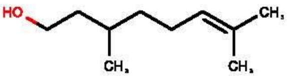	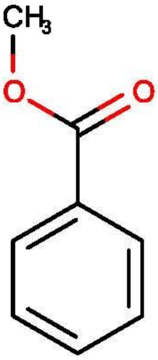	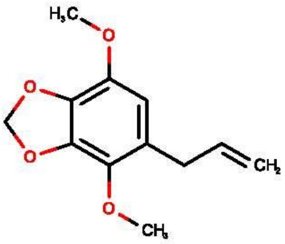
QMAYBMKBYCGXDH	QMVPMAAFGQKVCJ	QPJVMBTYPHYUOC	QQRSPHJOOXUALR

385	386	387	388
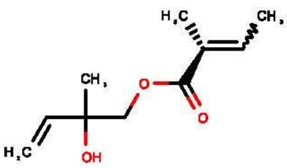	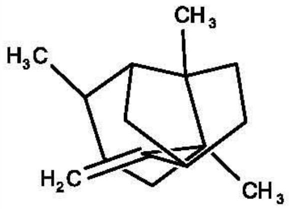	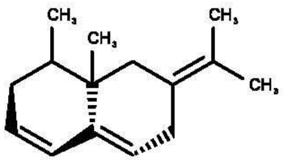	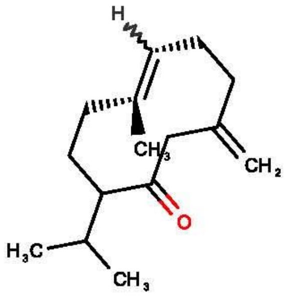
QQSQGJPTALGCLH	QQWUXXGYAQMTAT	QSUQBXKPPUWLTH	QTFJNWQFKJITEE
**389**	**390**	**391**	**392**
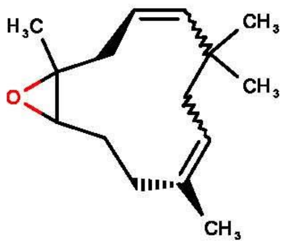	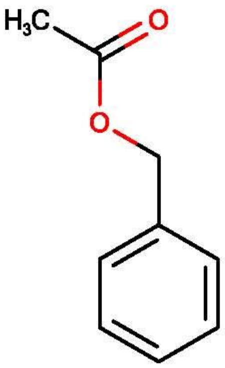	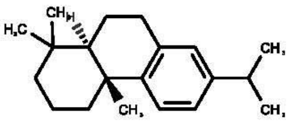	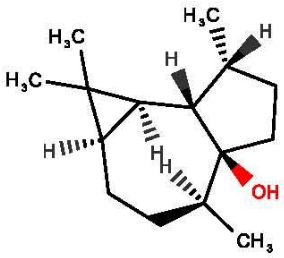
QTGAEXCCAPTGLB	QUKGYYKBILRGFE	QUUCYKKMFLJLFS	QWRTXOOFEHOROQ
**393**	**394**	**395**	**396**
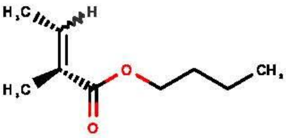	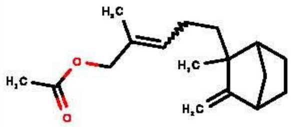	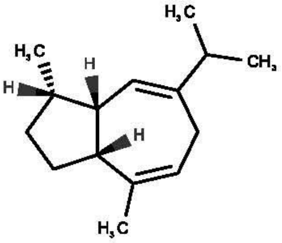	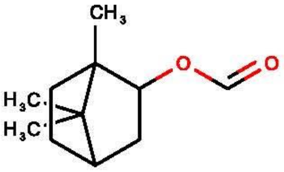
RBGFLIOXJWFKKX	RCFGRZLLBGMERD	RCMUGHFHXFHKNW	RDWUNORUTVEHJF
**397**	**398**	**399**	**400**
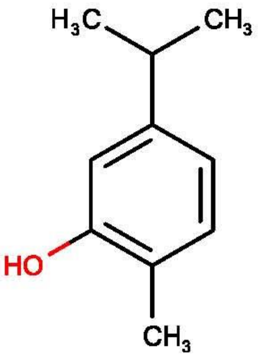	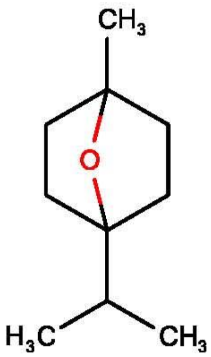	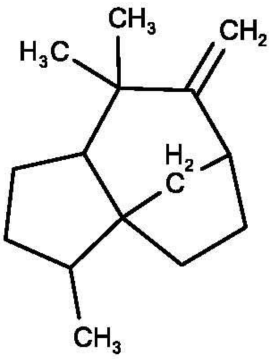	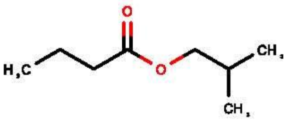
RECUKUPTGUEGMW	RFFOTVCVTJUTAD	RFSYBMDOYOBTCL	RGFNRWTWDWVHDD

401	402	403	404
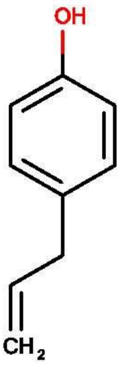	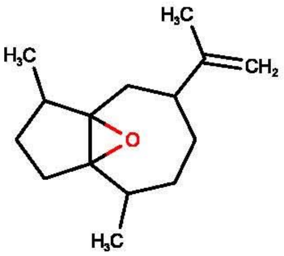	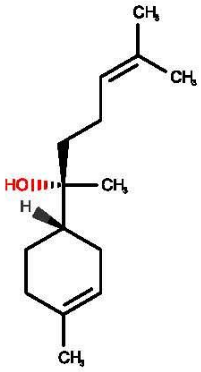	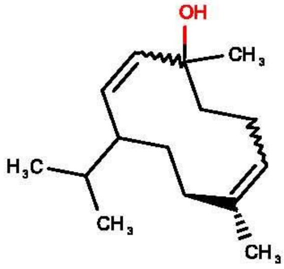
RGIBXDHONMXTLI	RGRYQUWGSJPMMK	RGZSQWQPBWRIAQ	RHCTXHCNRLCYBN
**405**	**406**	**407**	**408**
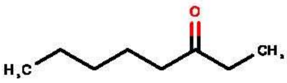	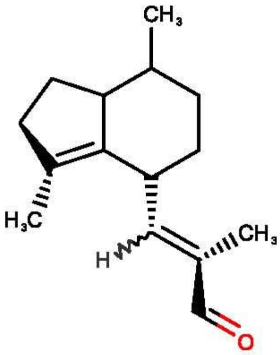	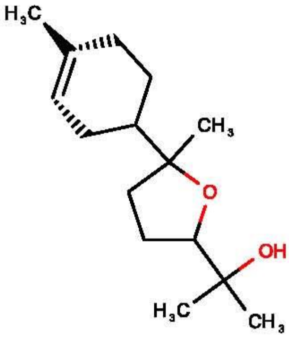	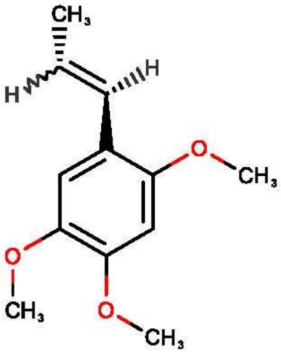
RHLVCLIPMVJYKS	RJZWGDPBGWGJNU	RKBAYVATPNYHLW	RKFAZBXYICVSKP
**409**	**410**	**411**	**412**
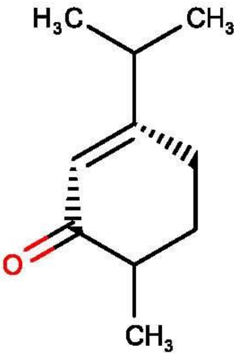	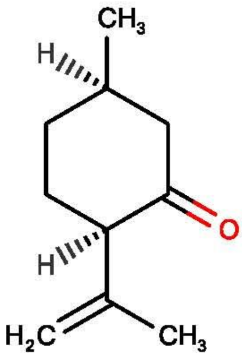	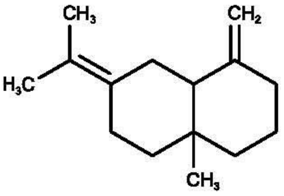	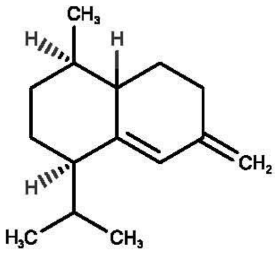
RLYSXAZAJUMULG	RMIANEGNSBUGDJ	RMZHSBMIZBMVMN	RNDFUOKDULDZPR
**413**	**414**	**415**	**416**
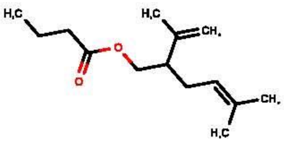	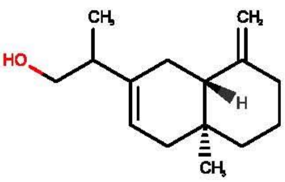	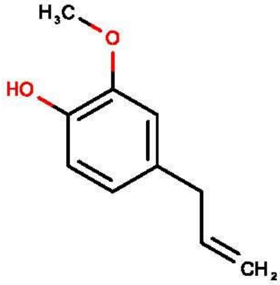	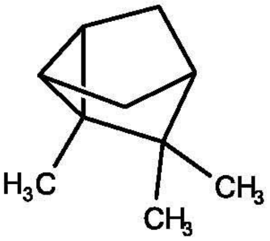
ROJSUZRYGHOLJL	RQWRGJGCTMAFBS	RRAFCDWBNXTKKO	RRBYUSWBLVXTQN

417	418	419	420
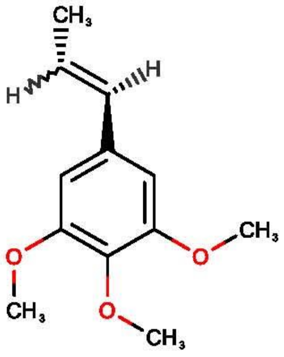	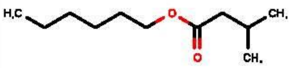	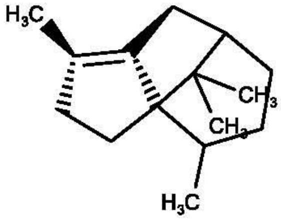	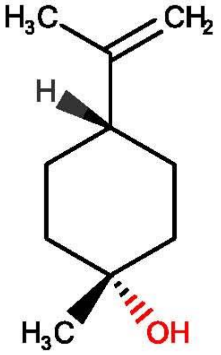
RRXOQHQFJOQLQR	RSDDTPVXLMVLQE	RTBLDXVIGWSICW	RUJPNZNXGCHGID

421	422	423	424
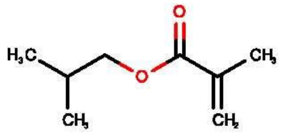	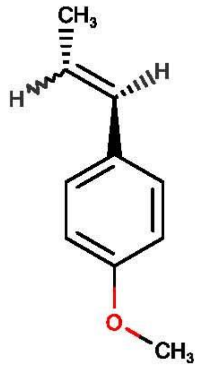	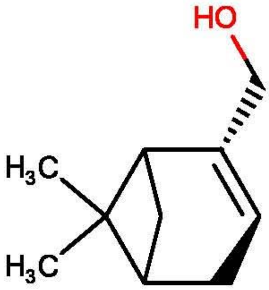	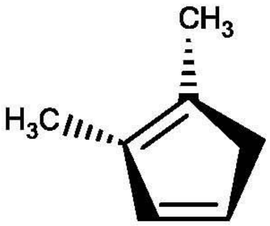
RUMACXVDVNRZJZ	RUVINXPYWBROJD	RXBQNMWIQKOSCS	RYILSJIMFKKICJ

425	426	427	428
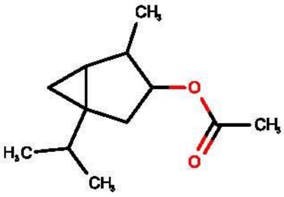	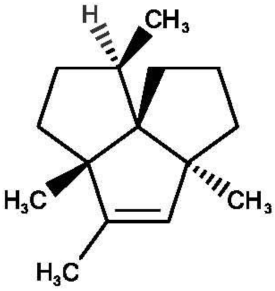	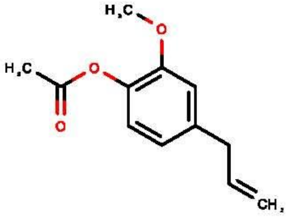	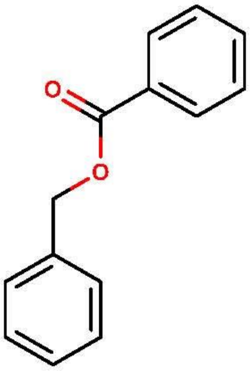
RYMWIDNPMDLHRP	SAOJPWFHRMUCFN	SCCDQYPEOIRVGX	SESFRYSPDFLNCH

429	430	431	432
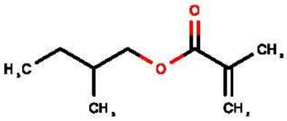	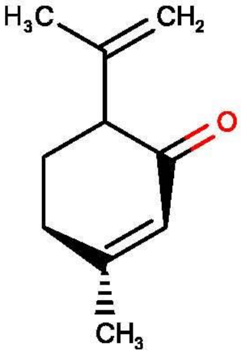	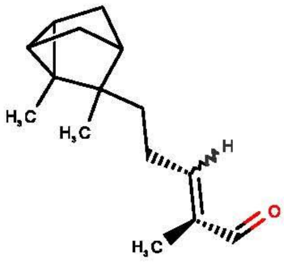	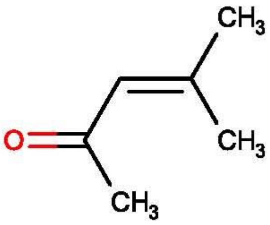
SESQOVINGFJWQN	SEZLYIWMVRUIKT	SHEUEEZGXLCROM	SHOJXDKTYKFBRD

433	434	435	436
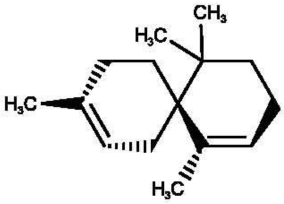	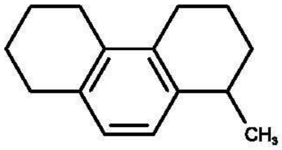	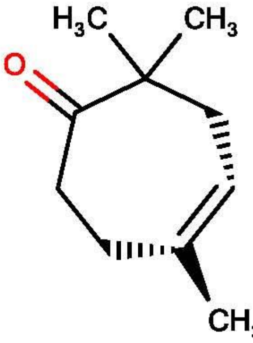	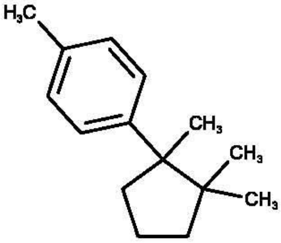
SIBCECUUMHIAAM	SKABIAPYJDXFMJ	SKKTZNHVYFHGDC	SLKPBCXNFNIJSV
**437**	**438**	**439**	**440**
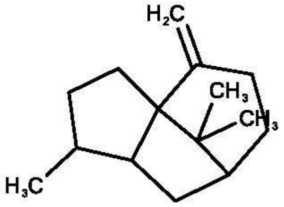	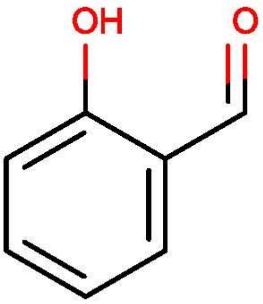	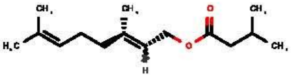	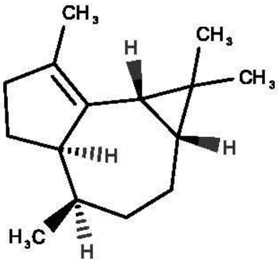
SLTLKLCDQWGISZ	SMQUZDBALVYZAC	SOUKTGNMIRUIQN	SPCXZDDGSGTVAW
**441**	**442**	**443**	**444**
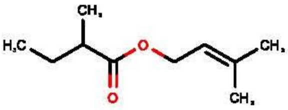	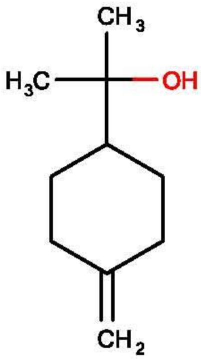	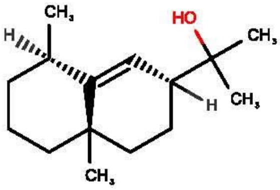	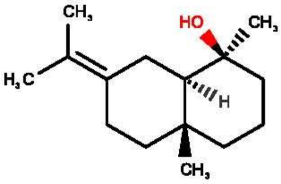
SPZJCVIOUQJWGX	SQIFACVGCPWBQZ	SRHDLIDOZXPROB	STRABSCAWZINIF
**445**	**446**	**447**	**448**
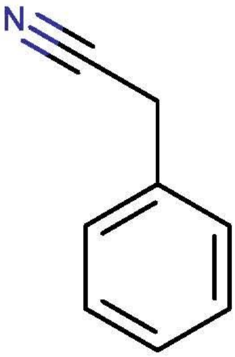	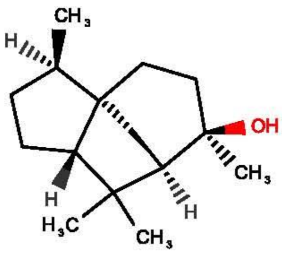	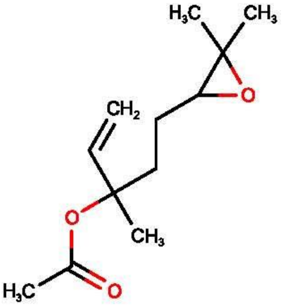	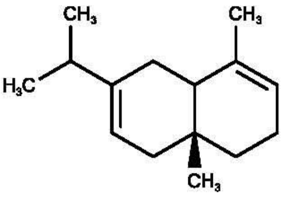
SUSQOBVLVYHIEX	SVURIXNDRWRAFU	SVXOLLYBCSDBKK	SWCOCRTVQQHGKB

449	450	451	452
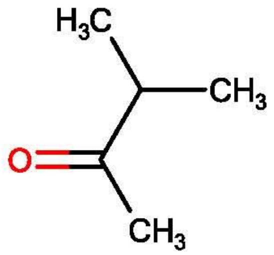	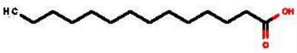	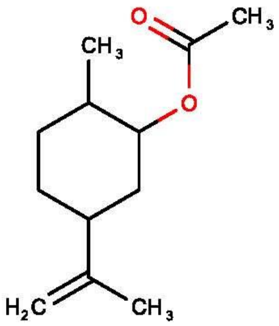	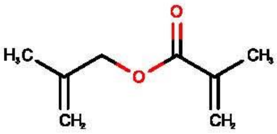
SYBYTAAJFKOIEJ	TUNFSRHWOTWDNC	TUSIZTVSUSBSQI	TVBLIEXOPWWREN
**453**	**454**	**455**	**456**
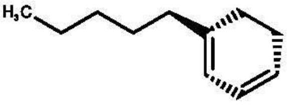	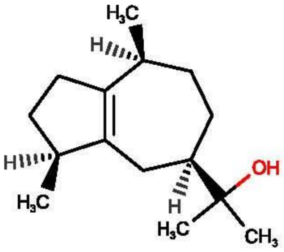	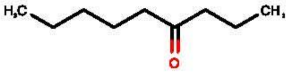	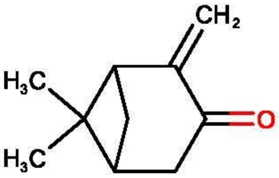
TWGRYUWSJLSKAL	TWVJWDMOZJXUID	TYBCSQFBSWACAA	TZDMGBLPGZXHJI
**457**	**458**	**459**	**460**
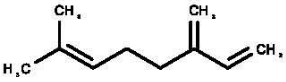	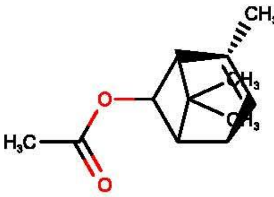	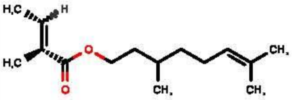	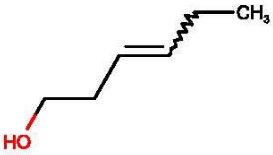
UAHWPYUMFXYFJY	UASZOTVHPVEMQR	UCFQYMKLDPWFHZ	UFLHIIWVXFIJGU
**461**	**462**	**463**	**464**
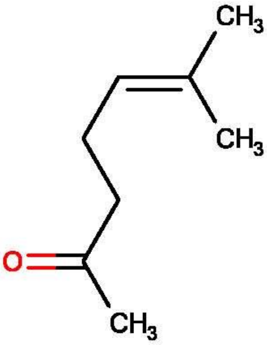	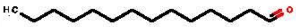	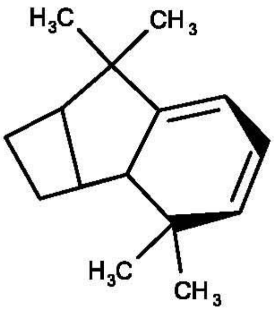	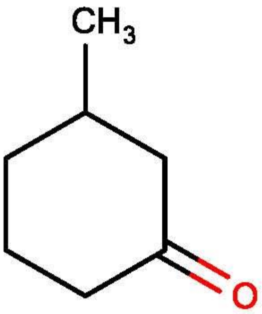
UHEPJGULSIKKTP	UHUFTBALEZWWIH	UIPKEVNEOFKIRG	UJBOOUHRTQVGRU

465

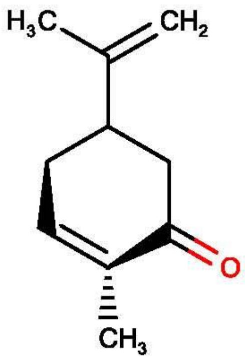

ULDHMXUKGWMISQ

466

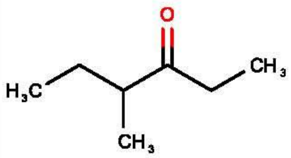

ULPMRIXXHGUZFA

467

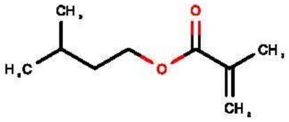

ULYIFEQRRINMJQ

468

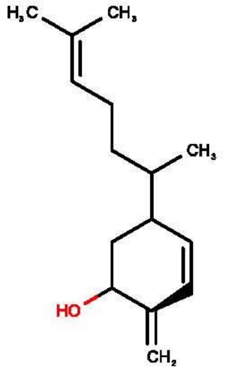

UNSGLJWOHGSVLW

469

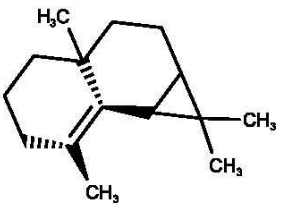

UPGLJTCDRBIZKP

470

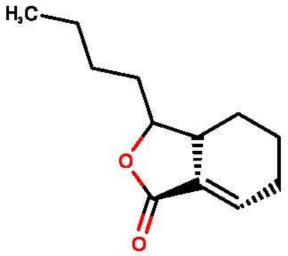

UPJFTVFLSIQQAV

471

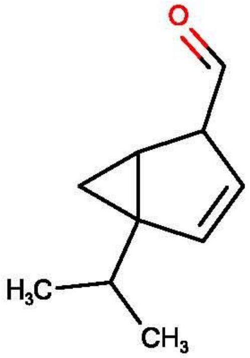

UQLNHDYBGCTZNJ

472

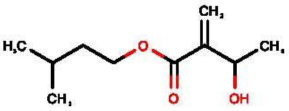

URBTVTZTLQVLCO

473

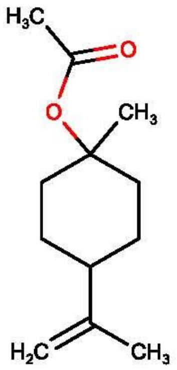

URVNHQCLMBMWIW

474

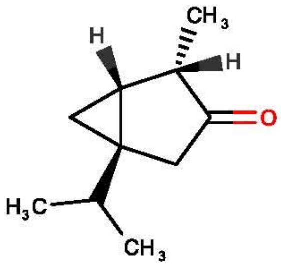

USMNOWBWPHYOEA

475

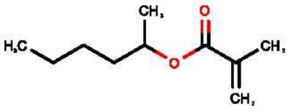

USPLKTKSLPLUJG

476

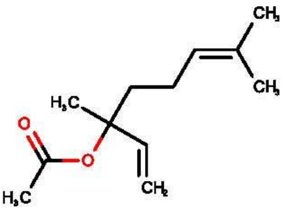

UWKAYLJWKGQEPM

477

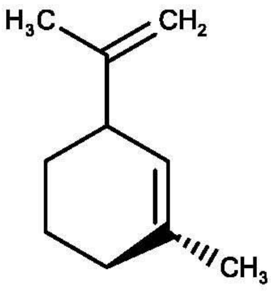

UXZIDIYMFIBDKT

478

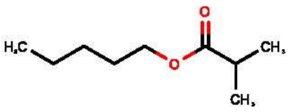

UYGGIIOLYXRSQY

479

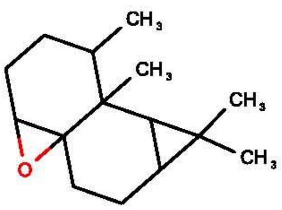

UYPPHUAQDGUVKN

480

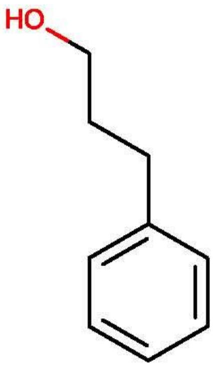

VAJVDSVGBWFCLW

481	482	483	484
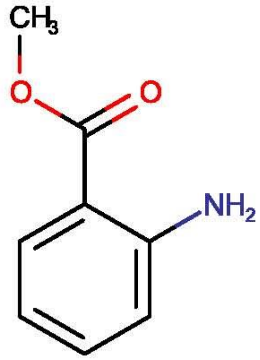	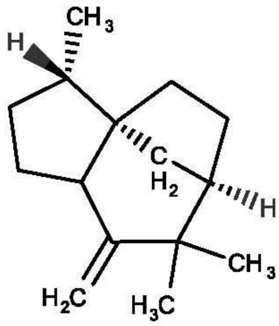	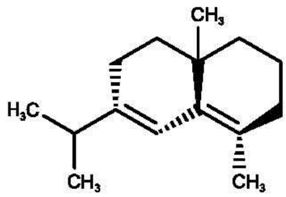	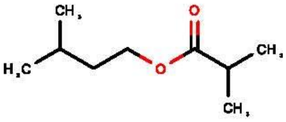
VAMXMNNIEUEQDV	VBZRHXLPRWBPEH	VEGYMPQCXPVQJY	VFTGLSWXJMRZNB
**485**	**486**	**487**	**488**
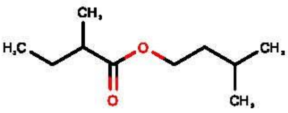	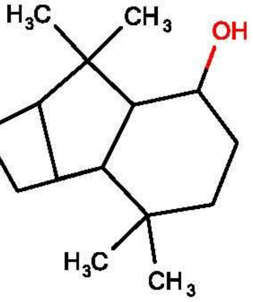	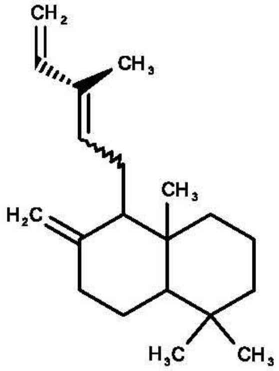	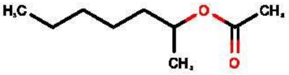
VGIRHYHLQKDEPP	VJCRIDPUJYUCNH	VJVMMXUPZGOBSN	VJYWBLDDQZIGJI
**489**	**490**	**491**	**492**
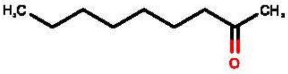	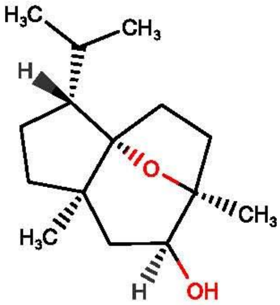	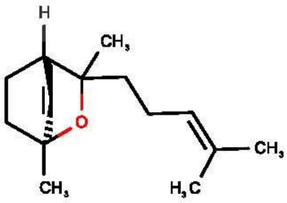	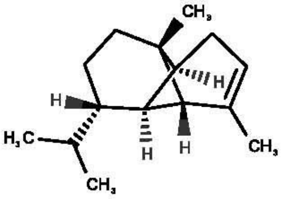
VKCYHJWLYTUGCC	VLIUMVVQGMLOJG	VLUGOADEEDGFLB	VLXDPFLIRFYIME
**493**	**494**	**495**	**496**
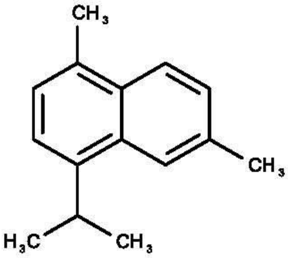	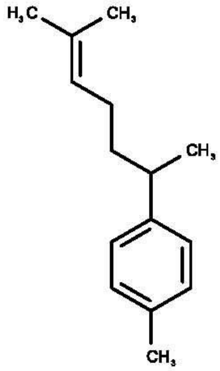	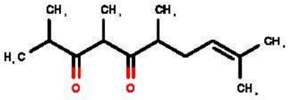	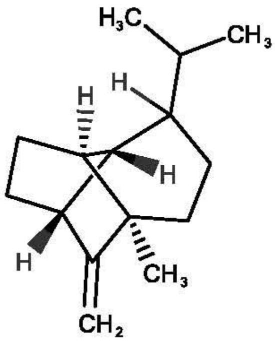
VMOJIHDTVZTGDO	VMYXUZSZMNBRCN	VNEFXHWAHYWLHU	VOBBUADSYROGAT

497	498	499	500
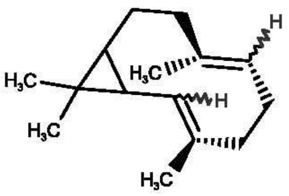	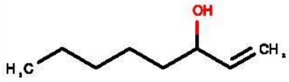	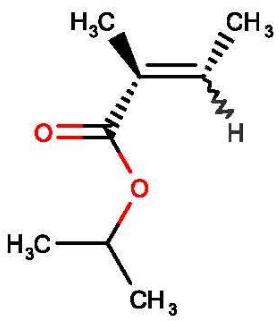	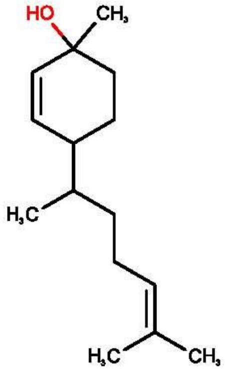
VPDZRSSKICPUEY	VSMOENVRRABVKN	VUPBIVVRPJDWNW	VVCHIOKYQRUBED
**501**	**502**	**503**	**504**
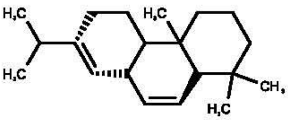	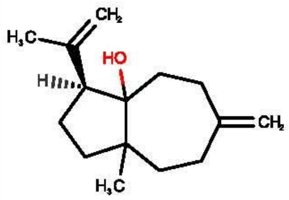	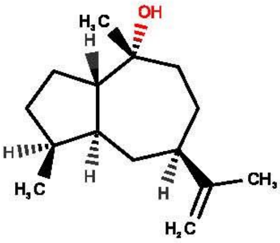	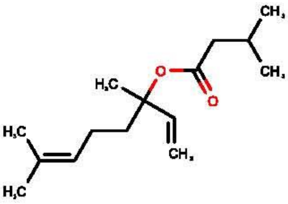
VVFXXDIPJNSMPW	VWRBCQWXFAGZPC	VYOZKWKETGHHDW	WCDGWAIZRYMVOW
**505**	**506**	**507**	**508**
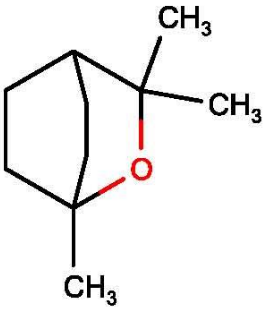	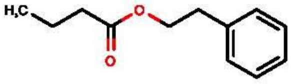	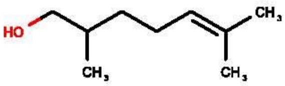	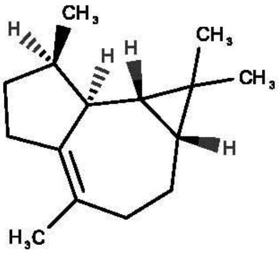
WEEGYLXZBRQIMU	WFNDDSQUKATKNX	WFZFXUZFKAOTRR	WGTRJVCFDUCKCM
**509**	**510**	**511**	**512**
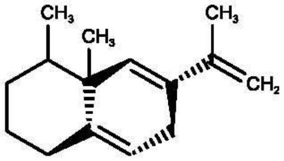	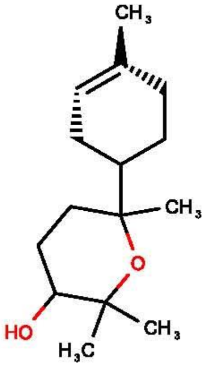	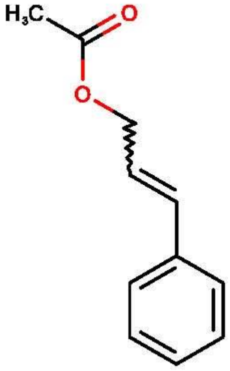	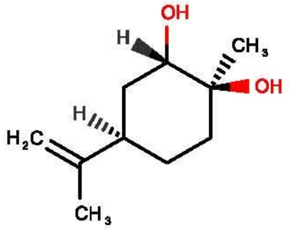
WHNNPKNATREGBK	WJHRAVIQWFQMKF	WJSDHUCWMSHDCR	WKZWTZTZWGWEGE

513

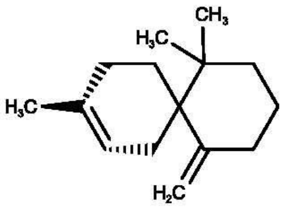

WLNGPDPILFYWKF

514

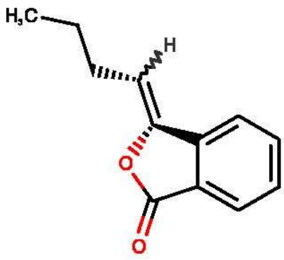

WMBOCUXXNSOQHM

515

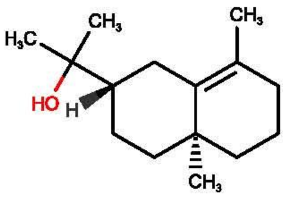

WMOPMQRJLLIEJV

516

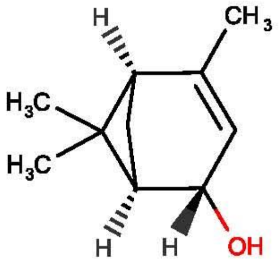

WONIGEXYPVIKFS

517

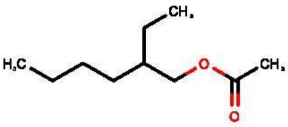

WOYWLLHHWAMFCB

518

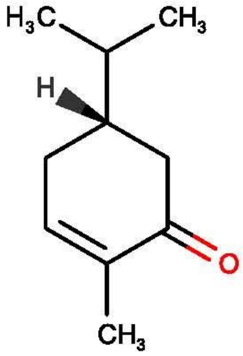

WPGPCDVQHXOMQP

519

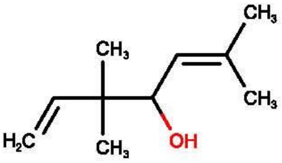

WPPVSYVQAKQNJK

520

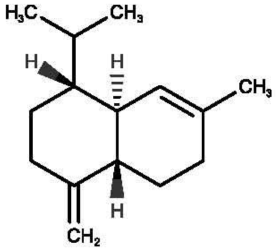

WRHGORWNJGOVQY

521

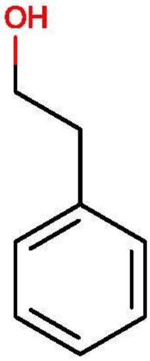

WRMNZCZEMHIOCP

522

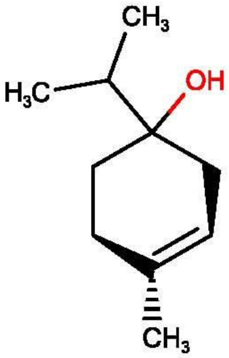

WRYLYDPHFGVWKC

523

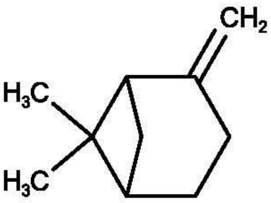

WTARULDDTDQWMU

524

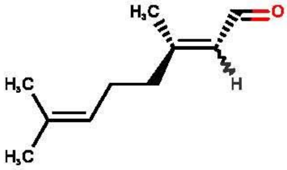

WTEVQBCEXWBHNA

525

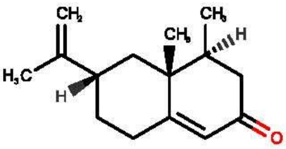

WTOYNNBCKUYIKC

526

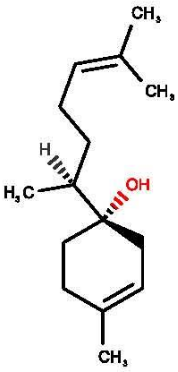

WTVHAMTYZJGJLJ

527

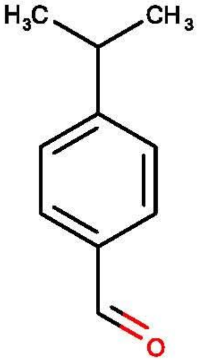

WTWBUQJHJGUZCY

528

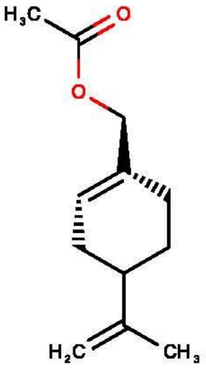

WTXBCFKGCNWPLS

529	530	531	532
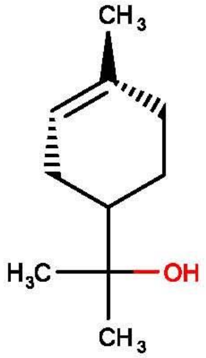	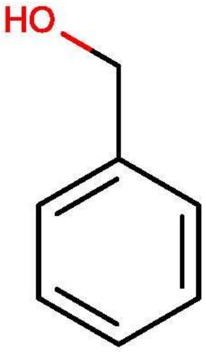	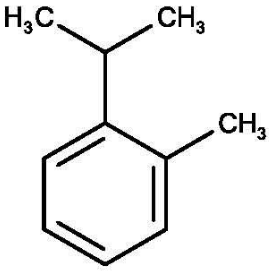	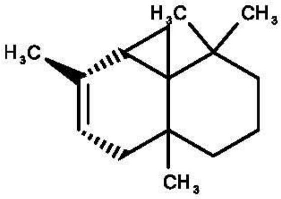
WUOACPNHFRMFPN	WVDDGKGOMKODPV	WWRCMNKATXZARA	WXQGPFZDVCRBME
**533**	**534**	**535**	**536**
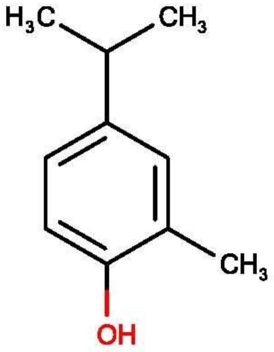	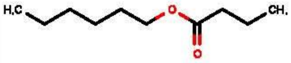	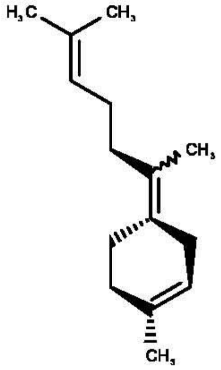	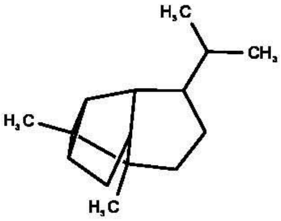
WYXXLXHHWYNKJF	XAPCMTMQBXLDBB	XBGUIVFBMBVUEG	XBWACJDEQIZTPR
**537**	**538**	**539**	**540**
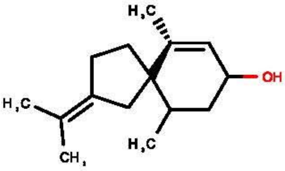	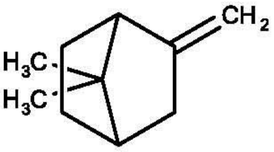	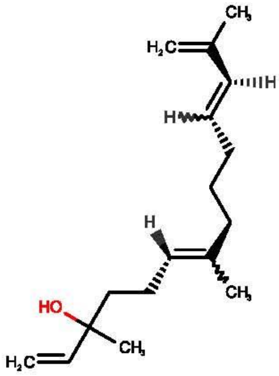	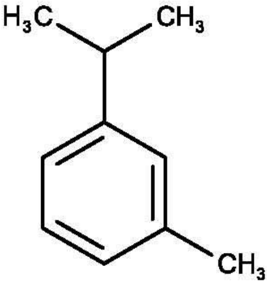
XCEXBRKEGXBUJE	XCPQUQHBVVXMRQ	XCTSDLUOCRRDLZ	XCYJPXQACVEIOS
**541**	**542**	**543**	**544**
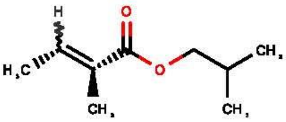	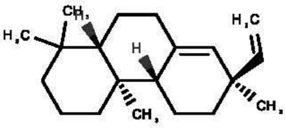	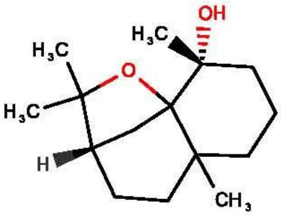	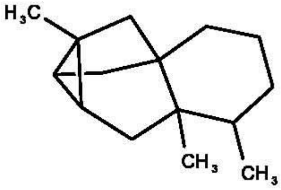
XDEGQMQKHFPBEW	XDSYKASBVOZOAG	XEAXSPJWIVZRTF	XGEWXQPYPMTSBD

545	546	547	548
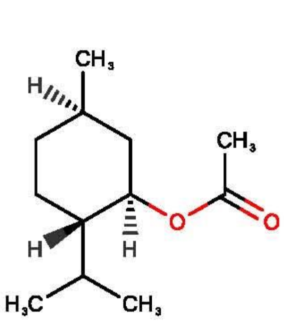	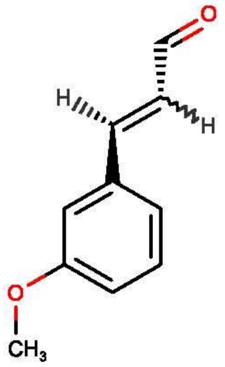	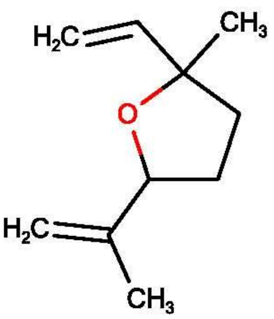	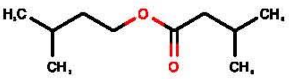
XHXUANMFYXWVNG	XHYAQFCRAQUBTD	XIGFNCYVSHOLIF	XINCECQTMHSORG
**549**	**550**	**551**	**552**
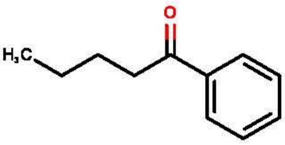	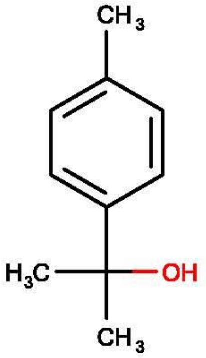	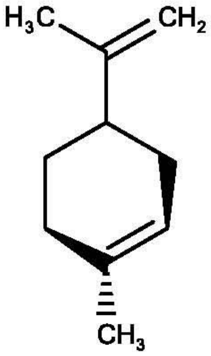	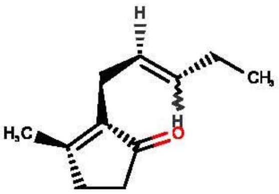
XKGLSKVNOSHTAD	XLPDVYGDNRIQFV	XMGQYMWWDOXHJM	XMLSXPIVAXONDL
**553**	**554**	**555**	**556**
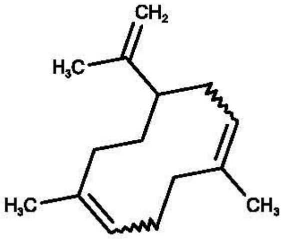	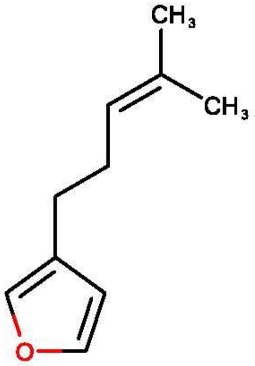	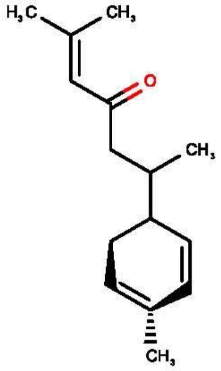	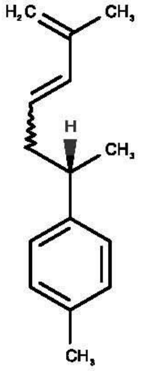
XMRKUJJDDKYUHV	XNGKCOFXDHYSGR	XOCANRBEOZQNAQ	XPCMQNXXPOZEHX
**557**	**558**	**559**	**560**
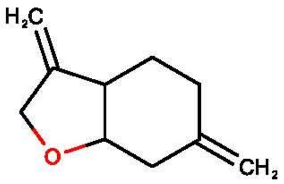	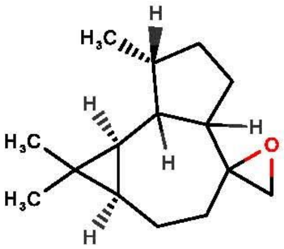	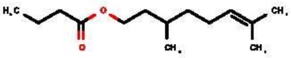	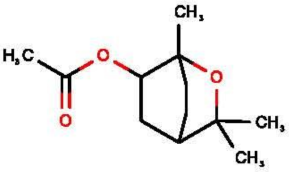
XPDXSIYURCKZBY	XPGWKKLDFXNBPJ	XQPZQXTWYZAXAK	XRKZFZWIYZDOQO

561	562	563	564
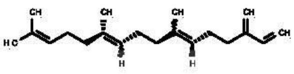	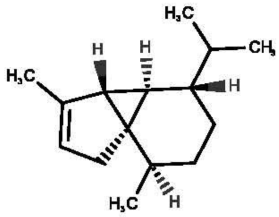	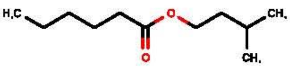	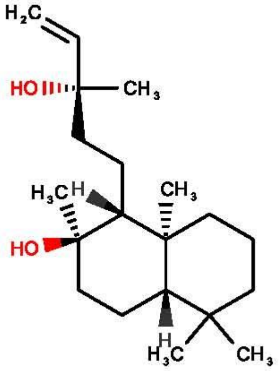
XSIVJVJUIXOEPW	XUEHVOLRMXNRKQ	XVSZRAWFCDHCBP	XVULBTBTFGYVRC
**565**	**566**	**567**	**568**
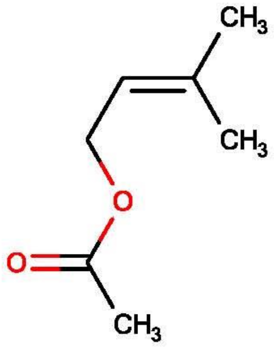	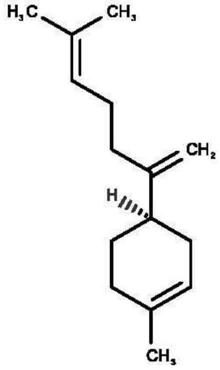	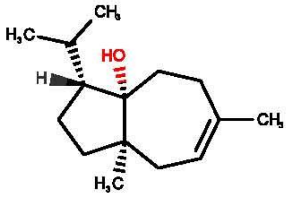	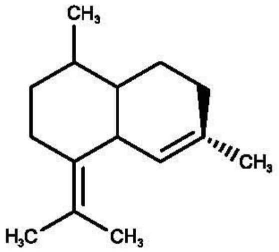
XXIKYCPRDXIMQM	XZRVRYFILCSYSP	XZYQCFABZDVOPN	YBEONGKDMARZSS
**569**	**570**	**571**	**572**
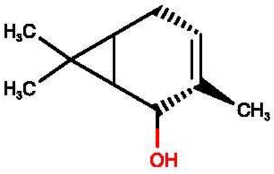	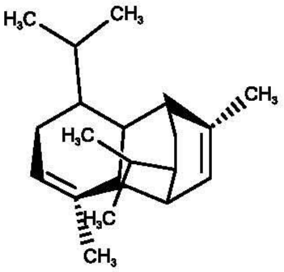	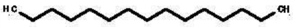	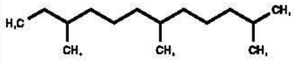
YCAQPZXDWPHYBD	YCGKKSPKOFEYNJ	YCOZIPAWZNQLMR	YFHFHLSMISYUAQ
**573**	**574**	**575**	**576**
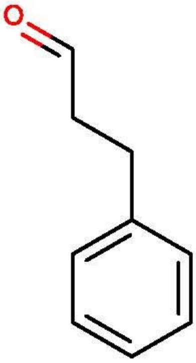	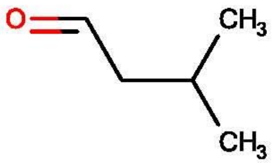	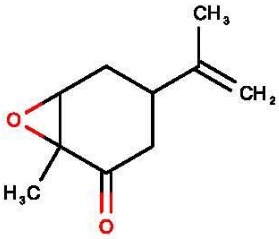	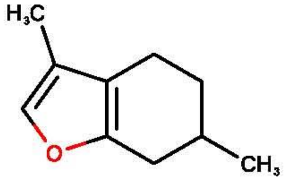
YGCZTXZTJXYWCO	YGHRJJRRZDOVPD	YGMNGQDLUQECTO	YGWKXXYGDYYFJU

577

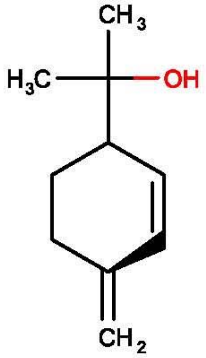

YGYSWSPXSCQPRC

578

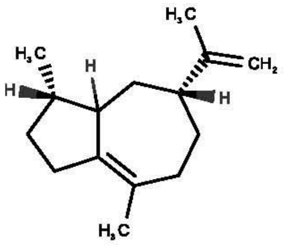

YHAJBLWYOIUHHM

579

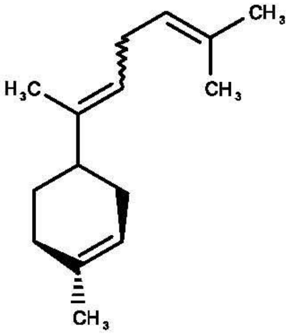

YHBUQBJHSRGZNF

580

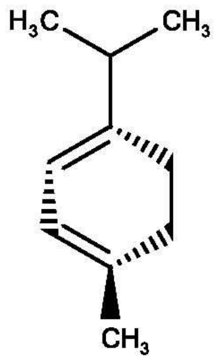

YHQGMYUVUMAZJR

581

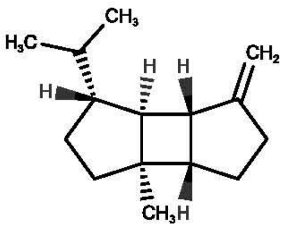

YIRAHEODBQONHI

582

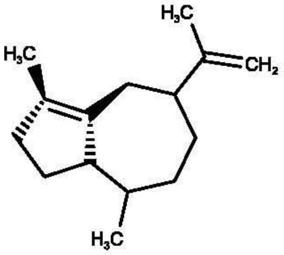

YIWKBKBHKZAWQV

583

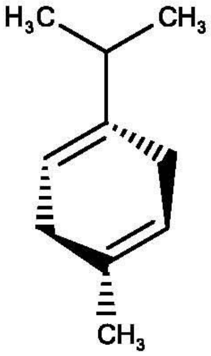

YKFLAYDHMOASIY

584

YKNWIILGEFFOPE

585

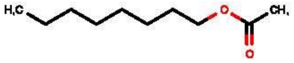

YLYBTZIQSIBWLI

586

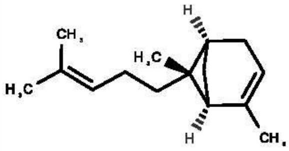

YMBFCQPIMVLNIU

587

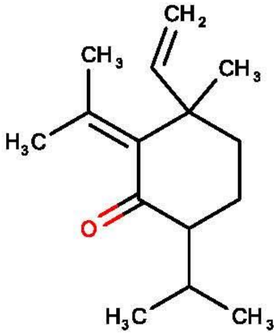

YMIHAYABXZORPU

588

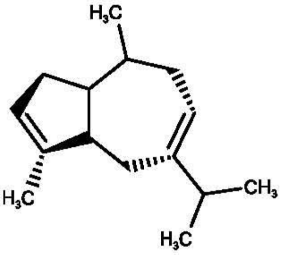

YOIKPWLIIDGZLM

589

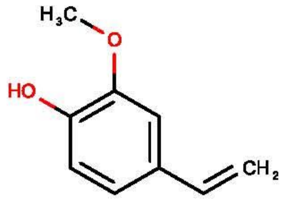

YOMSJEATGXXYPX

590

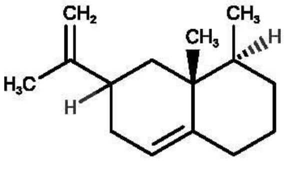

YONHOSLUBQJXPR

591

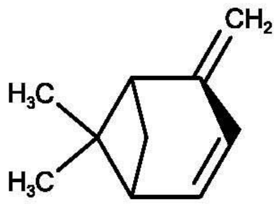

YOQFOABVDRBYCG

592

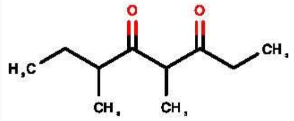

YOTJIUKJWGJMEV

593

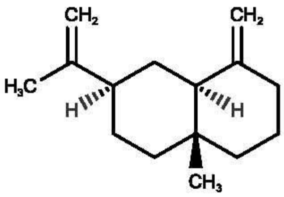

YOVSPTNQHMDJAG

594

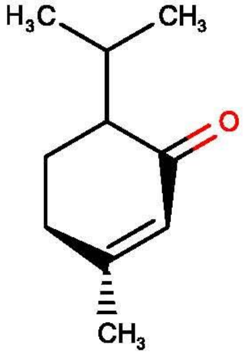

YSTPAHQEHQSRJD

595

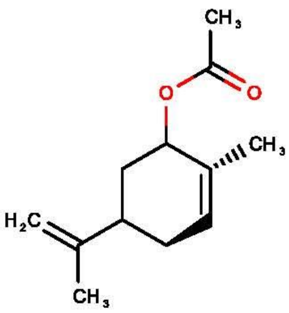

YTHRBOFHFYZBRJ

596

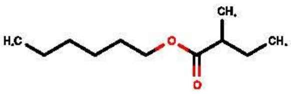

YUECNVSODFDKOQ

597

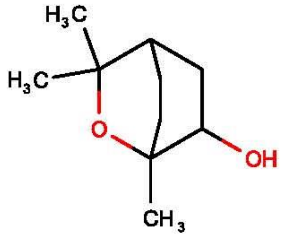

YVCUGZBVCHODNB

598

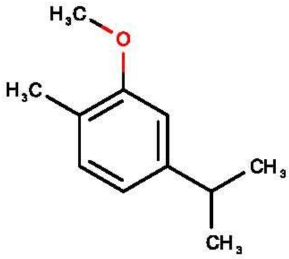

YVLHTQPPMZOCOW

599

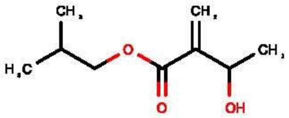

YWLLUDSCDSOEDO

600

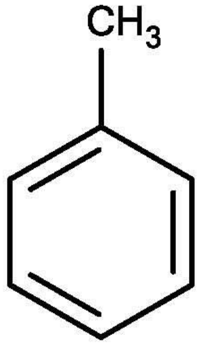

YXFVVABEGXRONW

601

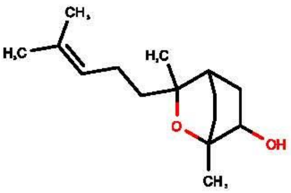

YXKYEDPZIRLAKN

602

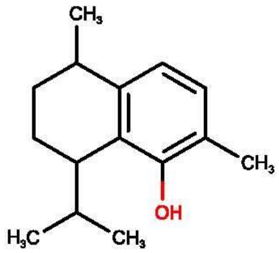

YXYMGKMWKSMRAB

603

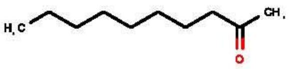

ZAJNGDIORYACQU

604

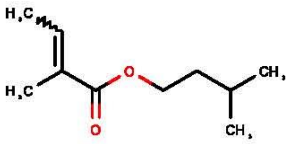

ZARFDQHJMNVNLE

605

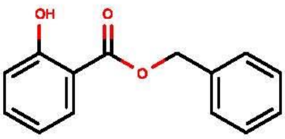

ZCTQGTTXIYCGGC

606

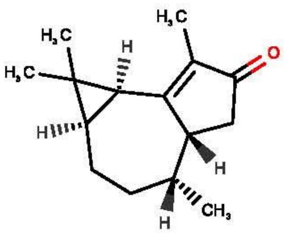

ZEEUIOBUKGZKPS

607

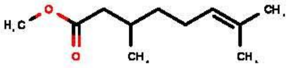

ZFLPOPCZMXGUOJ

608

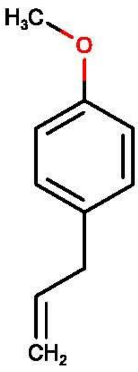

ZFMSMUAANRJZFM

609	610	611	612
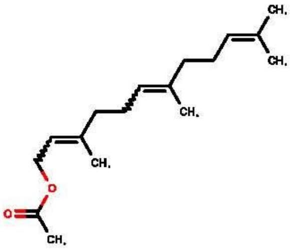	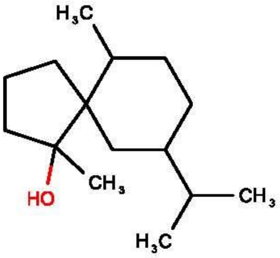	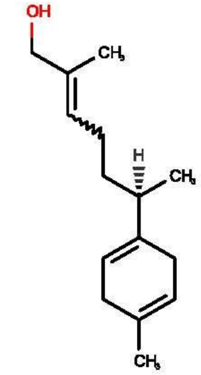	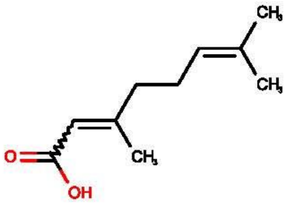
ZGIGZINMAOQWLX	ZGZJEDMRXISZCH	ZHWZEHFYKZGQFR	ZHYZQXUYZJNEHD
**613**	**614**	**615**	**616**
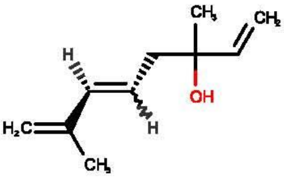	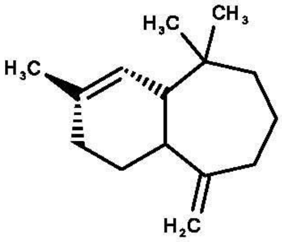	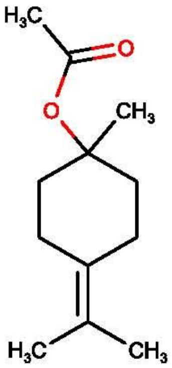	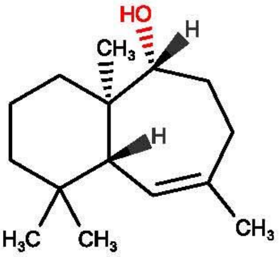
ZJIQIJIQBTVTDY	ZJSIKVDEOWWVEH	ZKKBZSOYCMSYRW	ZLJPQFLGGAYZAN
**617**	**618**	**619**	**620**
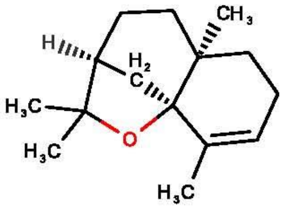	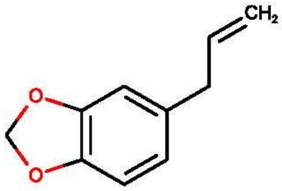	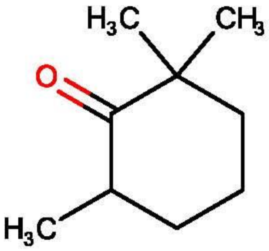	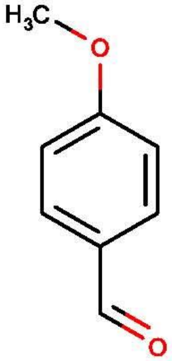
ZLQADKTVJQXDIG	ZMQAAUBTXCXRIC	ZPVOLGVTNLDBFI	ZRSNZINYAWTAHE
**621**	**622**	**623**	**624**
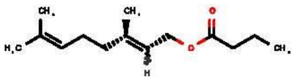	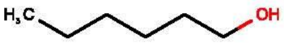	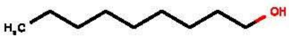	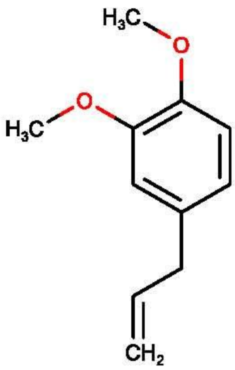
ZSBOMYJPSRFZAL	ZSIAUFGUXNUGDI	ZWRUINPWMLAQRD	ZYEMGPIYFIJGTP

625	626	627
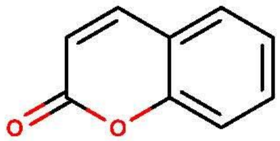	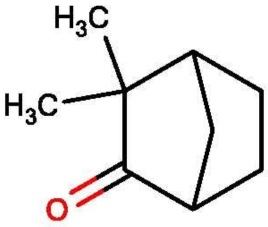	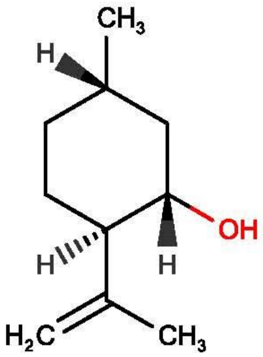
ZYGHJZDHTFUPRJ	ZYPYEBYNXWUCEA	ZYTMANIQRDEHIO

SI 3. Frequency rank order of u-cmcEOC in EO (sub)sets. Subset of (**A**) EOs from conventional cultivation (n_{EO}=101); (**B**) EOs from certified-organic cultivation (n_{EO}=74); (**C**) all EOs of conventional cultivation, complemented with those EOs of certified-organic cultivation that originated from other plant species, the same plant species but from other plant parts, or the same plant species but a different chemotype (n_{EO}=141). **(D)** complete EO sample (n_{EO}=175). See SI 1 for list with EOs

	**u-cmcEOC rank order in (sub)set X of the EO collection according to frequency of occurrence**			
	**A ($n_{u\text{-}cmcEOC}$=518)**	**B ($n_{u\text{-}cmcEOC}$=447)**	**C ($n_{u\text{-}cmcEOC}$=616)**	**D ($n_{u\text{-}cmcEOC}$=627)**
**limonene**	1	1	1	1
**alpha-pinene**	2	2	2	2
**beta-myrcene**	3	3	3	3
**beta-caryophyllene**	4	4	4	4
**beta-pinene**	5	5	5	5

SI 4: Correlations between the Drug Discovery Parameters used in the Drug Discovery Filters. (**a**) Table with Spearman correlations and their corresponding (**b**) p-values. ns = not significant (p-value > 0.05)

a.

Spearman correlation	molecular mass (Da)	log P	H-donor atoms (#)	H-acceptor atoms (#)	log D (pH 7.4)	molecular rings (#)	rotatable bonds (#)	atoms (#)	molar refractivity (m3 mol−1)	C atoms (#)	Muegge's atoms (#)	polar surface area (10^{-10} m)	fused aromatic rings (#)
**molecular mass (Da)**		0.68	0.10	0.01	0.67	0.34	0.10	0.94	0.94	0.92	0.03	0.07	-0.05
**log P**	0.68		-0.24	-0.53	0.99	0.16	0.09	0.75	0.80	0.81	-0.44	-0.44	0.01
**H-donor atoms (#)**	0.10	-0.24		0.37	-0.26	0.03	-0.08	0.11	0.04	-0.01	0.11	0.33	-0.05
**H-acceptor atoms (#)**	0.01	-0.53	0.37		-0.54	-0.26	0.30	-0.17	-0.19	-0.30	0.85	0.84	-0.08
**log D (pH 7.4)**	0.67	0.99	-0.26	-0.54		0.16	0.09	0.74	0.79	0.80	-0.45	-0.46	0.01
**molecular rings (#)**	0.34	0.16	0.03	-0.26	0.16		-0.74	0.32	0.27	0.48	-0.39	-0.37	0.06
**rotatable bonds (#)**	0.10	0.09	-0.08	0.30	0.09	-0.74		0.05	0.11	-0.07	0.49	0.46	-0.08
**atoms (#)**	0.94	0.75	0.11	-0.17	0.74	0.32	0.05		0.92	0.92	-0.17	-0.11	-0.07
**molar refractivity (m3 mol−1)**	0.94	0.80	0.04	-0.19	0.79	0.27	0.11	0.92		0.96	-0.18	-0.13	-0.04
**C atoms (#)**	0.92	0.81	-0.01	-0.30	0.80	0.48	-0.07	0.92	0.96		-0.30	-0.26	-0.03
**Muegge's atoms (#)**	0.03	-0.44	0.11	0.85	-0.45	-0.39	0.49	-0.17	-0.18	-0.30		0.95	-0.03
**polar surface area (10^{-10} m)**	0.07	-0.44	0.33	0.84	-0.46	-0.37	0.46	-0.11	-0.13	-0.26	0.95		-0.05
**fused aromatic rings (#)**	-0.05	0.01	-0.05	-0.08	0.01	0.06	-0.08	-0.07	-0.04	-0.03	-0.03	-0.05	

b.

p-values	molecular mass (Da)	log P	H-donor atoms (#)	H-acceptor atoms (#)	log D (pH 7.4)	molecular rings (#)	rotatable bonds (#)	atoms (#)	molar refractivity (m3 mol−1)	C atoms (#)	Muegge's atoms (#)	polar surface area (10^{-10} m)	fused aromatic rings (#)
**molecular mass (Da)**		0.0000	0.0118	n	0.0000	0.0000	0.0143	0.0000	0.0000	0.0000	ns	0.0902	ns
**log P**	0.0000		0.0000	0.0000	0.0000	0.0001	0.0185	0.0000	0.0000	0.0000	0.0000	0.0000	ns
**H-donor atoms (#)**	0.0118	0.0000		0.0000	0.0000	ns	ns	0.0076	ns	ns	0.0081	0.0000	ns
**H-acceptor atoms (#)**	ns	0.0000	0.0000		0.0000	0.0000	0.0000	0.0000	0.0000	0.0000	0.0000	0.0000	0.0352
**log D (pH 7.4)**	0.0000	0.0000	0.0000	0.0000		0.0001	0.0292	0.0000	0.0000	0.0000	0.0000	0.0000	ns
**molecular rings (#)**	0.0000	0.0001	ns	0.0000	0.0001		0.0000	0.0000	0.0000	0.0000	0.0000	0.0000	ns
**rotatable bonds (#)**	0.0143	0.0185	ns	0.0000	0.0292	0.0000		ns	0.0074	ns	0.0000	0.0000	ns
**atoms (#)**	0.0000	0.0000	0.0076	0.0000	0.0000	0.0000	ns		0.0000	0.0000	0.0000	0.0085	ns
**molar refractivity (m3 mol−1)**	0.0000	0.0000	ns	0.0000	0.0000	0.0000	0.0074	0.0000		0.0000	0.0000	0.0008	ns
**C atoms (#)**	0.0000	0.0000	ns	0.0000	0.0000	0.0000	ns	0.0000	0.0000		0.0000	0.0000	ns
**Muegge's atoms (#)**	ns	0.0000	0.0081	0.0000	0.0000	0.0000	0.0000	0.0000	0.0000	0.0000		0.0000	ns
**polar surface area (10^{-10} m)**	ns	0.0000	0.0000	0.0000	0.0000	0.0000	0.0000	0.0085	0.0008	0.0000	0.0000		ns
**fused aromatic rings (#)**	ns	ns	ns	0.0352	ns	ns	ns	ns	ns	ns	ns	ns	

SI 5. Summary of u-cmcEOCs in EO set (n=175) that correspond with u-cmcADs in DrugBank (n=2,359) including their status [64].

	InChIKey-14	u-cmcEOC code in SI 2	u-cmcAD code in DrugBase	status in DrugBase	
				approved [§]	withdrawn [£]
1	BJIOGJUNALELMI	29	DB14188	yes	no
2	DSSYKIVIOFKYAU	77	DB14156	yes	no
3	GLZPCOQZEFWAFX	130	DB14183	yes	no
4	KJPRLNWUNMBNBZ [&]	227	DB14184	yes	no
5	LFQSCWFLJHTTHZ	254	DB00898	yes	no
6	MGSRCZKZVOBKFT	277	DB02413	yes	no
7	NOOLISFMXDJSKH	315	DB00825	yes	no
8	OOCCDEMITAIZTP	341	DB14186	yes	no
9	OSWPMRLSEDHDFF	346	DB09543	yes	no
10	RGZSQWQPBWRIAQ	403	DB13153	yes	no
11	RRAFCDWBNXTKKO	415	DB09086	yes	no
12	SESFRYSPDFLNCH	428	DB00676	yes	no
13	WVDDGKGOMKODPV	530	DB06770	yes	no

[&] This InChIKey-14 corresponds with ethanol; for more details, see the discussion section. [§] approved: "In DrugBank the status of approved is given to the drugs that have been officially accepted for commercialization in at least one jurisdiction at a given time. Due to the presence of different regulatory agencies around the world, once a drug is approved, this status will remain in the drug card even if the drug is later [withdrawn] from the market in a particular jurisdiction or under investigation for a different indication." [£] Withdrawn: "In DrugBank this state is given to the drugs that have been discontinued. Although the reason as to why such drugs are withdrawn can range from patient safety and toxicity issues to limited commercial viability, the formal rationale behind such decisions lies typically with the regional or national public health administration that issued the withdrawal" https://dev.drugbankplus.com/guides/terms/approved-drug

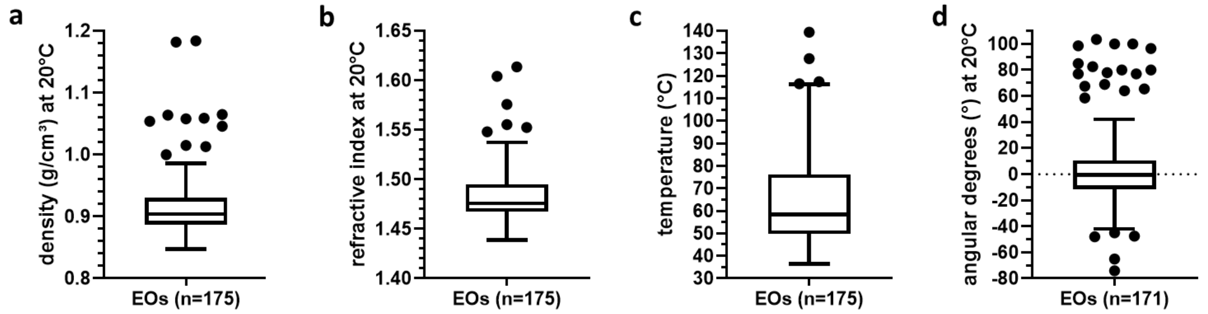

SI 6: Summary of selected characteristics related to quality control of the EOs we used. Tukey boxplot [minimum; 25th percentile; median; 75th percentile; maximum] (unit of measurement) of: (**a**) density [0.85; 0.89; 0.90; 0.93; 1.18] (g/cm²) , (**b**) refractive index [1.439; 1.467; 1.476; 1.495; 1.614], (**c**) flashpoint [36.4; 49.6; 58.4; 76.3; 139.4] (°C), and (**d**) optical rotation [-74.0; -11.5; -0.5; 10.5; 103.5] (°).

a.

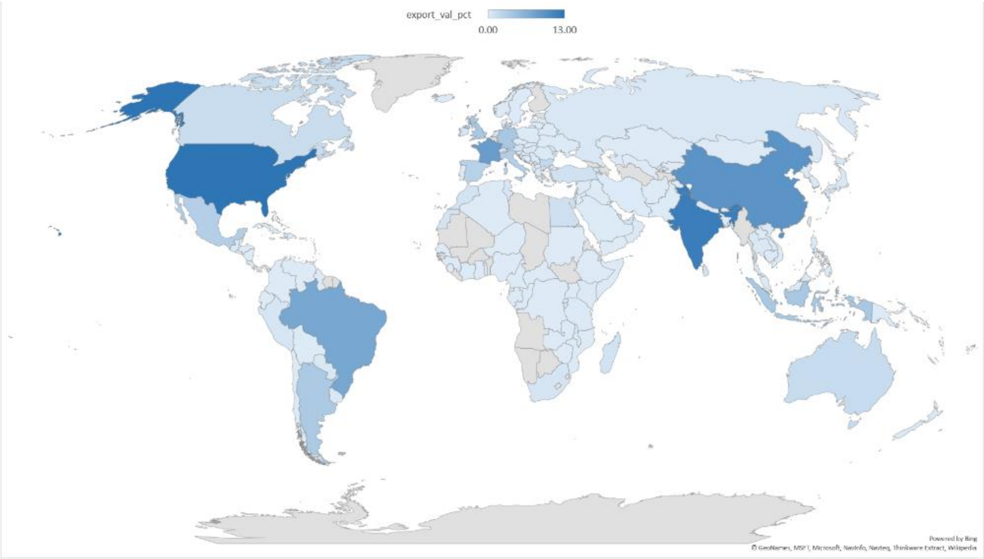

b.

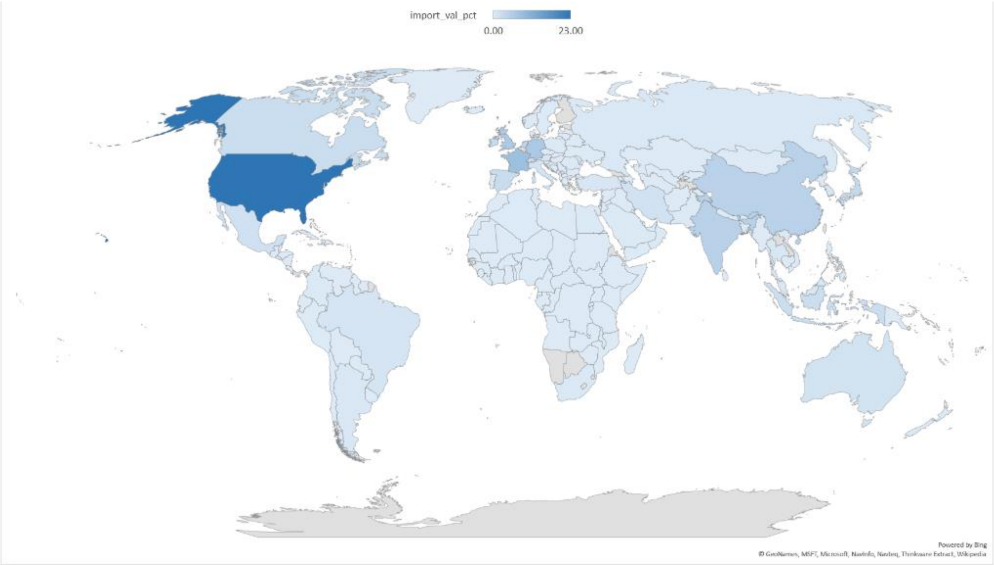

SI 7: World map visualizing percentage world (a) export and (b) import value of EOs per country (2017) [69].
